# Organization and differential expression of the GACA/GATA tagged somatic and spermatozoal transcriptomes in Buffalo *Bubalus bubalis*

**DOI:** 10.1186/1471-2164-9-132

**Published:** 2008-03-20

**Authors:** Jyoti Srivastava, Sanjay Premi, Sudhir Kumar, Sher Ali

**Affiliations:** 1 Molecular Genetics Laboratory, National Institute of Immunology, Aruna Asaf Ali Marg, New Delhi-110 067, India

## Abstract

**Background:**

Simple sequence repeats (SSRs) of GACA/GATA have been implicated with differentiation of sex-chromosomes and speciation. However, the organization of these repeats within genomes and transcriptomes, even in the best characterized organisms including human, remains unclear. The main objective of this study was to explore the buffalo transcriptome for its association with GACA/GATA repeats, and study the structural organization and differential expression of the GACA/GATA repeat tagged transcripts. Moreover, the distribution of GACA and GATA repeats in the prokaryotic and eukaryotic genomes was studied to highlight their significance in genome evolution.

**Results:**

We explored several genomes and transcriptomes, and observed total absence of these repeats in the prokaryotes, with their gradual accumulation in higher eukaryotes. Further, employing novel microsatellite associated sequence amplification (MASA) approach using varying length oligos based on GACA and GATA repeats; we identified and characterized 44 types of known and novel mRNA transcripts tagged with these repeats from different somatic tissues, gonads and spermatozoa of water buffalo *Bubalus bubalis*. GACA was found to be associated with higher number of transcripts compared to that with GATA. Exclusive presence of several GACA-tagged transcripts in a tissue or spermatozoa, and absence of the GATA-tagged ones in lung/heart highlights their tissue-specific significance. Of all the GACA/GATA tagged transcripts, ~30% demonstrated inter-tissue and/or tissue-spermatozoal sequence polymorphisms. Significantly, ~60% of the GACA-tagged and all the GATA-tagged transcripts showed highest or unique expression in the testis and/or spermatozoa. Moreover, ~75% GACA-tagged and all the GATA-tagged transcripts were found to be conserved across the species.

**Conclusion:**

Present study is a pioneer attempt exploring GACA/GATA tagged transcriptome in any mammalian species highlighting their tissue, stage and species-specific expression profiles. Comparative analysis suggests the gradual accumulation of these repeats in the higher eukaryotes, and establishes the GACA richness of the buffalo transcriptome. This is envisaged to establish the roles of integral simple sequence repeats and tagged transcripts in gene expression or regulation.

## Background

A predominant portion of the eukaryotic genome harbors different repetitive sequences while a small portion (2–3%) is transcribed and processed into mature transcripts [1-3]. Repetitive sequences are dynamic genome components encompassing transposable elements, major satellites and simple sequence repeats (SSRs) [4,5]. The highly polymorphic and multiallelic SSRs [6] are potentially involved in genome evolution by creating and maintaining genetic variability [2,7,8]. Most of these SSRs are found in non-coding regions of the genomes while a small fraction is retained in the transcriptome [2,3] participating in gene regulation through transcription, translation or gene silencing [9,10]. The expansion and contraction of SSRs within the protein-coding sequences are recognized to modulate disease risks such as Huntington's disease, Myotonic dystrophy and fragile X Syndrome [11-15]. However, the distribution of SSRs within non-coding and coding regions of the genomes, even in the best characterized ones such that of human, remains unclear. To explore the organization and expression of such repeat-tagged genes, we targeted the transcriptome of water buffalo *Bubalus bubalis *as a model system, an important player in the agriculture, dairy and meat industries in the Indian sub-continent. Novelty also lie in the fact that buffalo genome is unexplored in terms of genes present and its association with the SSRs.

Simple repeats, GATA and GACA, were identified from the satellite DNA of Banded krait in snakes and thus named as Banded krait minor (*Bkm*). Upon subsequent characterization, this was found to be conserved across the species including humans showing specific organization to the heterogametic (XY/ZW) sex chromosomes [16-18]. High condensation of these repeats in somatic cells and decondensation in germ cells during early stages of development, sex-/tissue-specific expression in higher eukaryotes were all thought to be involved in sex differentiation [19-21]. However, the organization of GACA/GATA repeats within the mRNA transcripts from both somatic tissues and spermatozoa remains largely unabsolved.

Ejaculated spermatozoa are terminally differentiated cells in which transcription and/or translation of nuclear encoded mRNAs are unlikely. Therefore, until recently, the male genome was the only cargo the spermatozoa were thought to carry. The discovery of many soluble signaling molecules, transcription factors and structures such as centriole being introduced by spermatozoan into the zygotic cytoplasm upon fertilization has changed this perception [22-24]. Despite transcriptionally dormant state, the spermatozoa retain an entourage of transcripts, encoding transcription factors and proteins involved in signal transduction, cell proliferation, DNA condensation, regulation of sperm motility, capacitation and acrosome reaction [24-28].

Owing to the tissue- and sex-specific organization of the GACA/GATA repeats and participation of the spermatozoal RNA during and post-syngamy, we studied the GACA/GATA tagged transcriptomes from the somatic/gonadal tissues and spermatozoa of buffalo *Bubalus bubalis*. The mRNA transcripts so uncovered were further characterized for their sequence organization, homology status, expressional variation, copy number and evolutionary status. Moreover, chromosomal mapping was done for the candidate genes tagged with GACA/GATA repeats. In addition, distribution of the GACA/GATA repeats within the genomes across the species was also studied.

## Results

### Genomic/Transcriptomic distribution of GACA/GATA across the species

**Figure 1:**
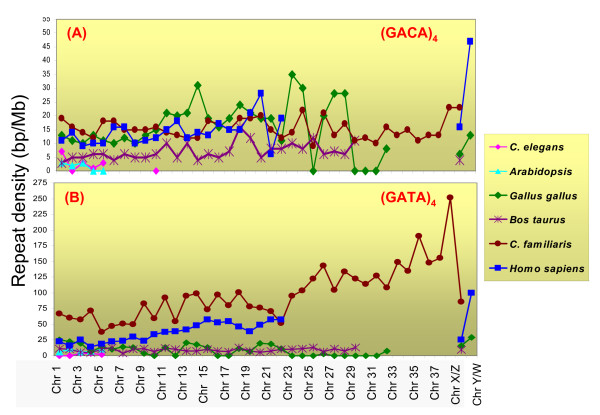
Chromosomal distribution of GACA **(A) **and GATA **(B) **repeats across the six eukaryotes based on *in-silico *analysis. The repeat density of the GACA/GATA tetramers across the chromosomes sets in different species is expressed in base-pairs per megabase of each chromosome. Note the differential occurrence of these repeats along different chromosomes. The human and dog genomes were found to be GATA rich. The GATA repeats were predominant on the human and chicken Y chromosomes. Status of these repeats on the Y chromosomes in other species remained unclear due to their unfinished genomes.

The *in-silico *analyses of the available complete or incomplete genomes of Archeas, Eubacteria and 17 eukaryotes including human revealed total absence of the GACA/GATA repeats in the prokaryotes and lower eukaryotes such as *Saccharomyces cerevisae and Dictyostelium discoideum *(Additional file 1). However, a gradual accumulation of these repeats was observed in the higher eukaryotes (Additional file 1). Detailed analysis of 6 species showed differential occurrence of the tetramers of GACA/GATA repeats among different chromosomes and species (Figure 1). Of these, the human, dog and *Arabidopsis *genomes were found to be GATA rich whereas chicken genome showed similar occurrence of the GACA/GATA tetramers. The cattle remained indecipherable due to its unfinished genome. The *C. elegans *genome was found to harbor only 13 regions containing tetramer of GACA and 12, GATA repeats. When considered individually, the highest occurrence of GACA was detected in chicken and that of GATA in dog. However, both GACA and GATA tetramers were concentrated on the Y chromosome in the humans. In case of dogs, the (GACA)_4 _was predominant on the chromosomes 38 and X and (GATA)_4 _on the chromosome 38. Distribution of these repeats on the Y chromosome of dogs could not be studied since their sequences have not been fully explored. The *Gallus gallus *showed maximum occurrence of GACA tetramer on the chromosome 23 and that of GATA on the chromosome Y (Figure 1).

Moreover, the analyses of the transcriptomes of the above mentioned species (Additional file 1) revealed the association of these *Bkm *derived repeats with several mRNA transcripts across the species. Comparative analysis showed that more number of transcripts was tagged with GACA repeat (Additional file 2) compared to that with GATA (Additional file 3). However, the GACA repeat was abundant in the mouse transcriptome, while GATA, in the human. Thus, a differential distribution of GACA/GATA repeats was observed in both the non-coding and coding regions of the genomes within and across the species.

### Identification and characterization of GACA/GATA tagged transcripts

**Figure 2:**
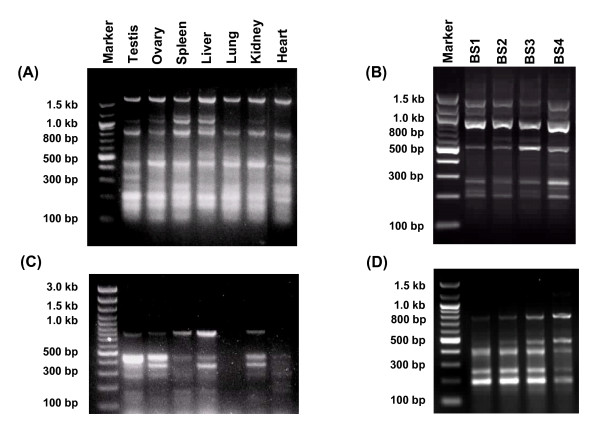
Microsatellite associated sequence amplification (MASA) performed using oligos based on varying lengths of GACA/GATA repeats and cDNA from different sources **(A-D)**. The amplified transcripts ranged from 0.15 kb to 1.8 kb. MASA using GACA repeat with cDNA from different somatic and gonadal tissues is given in **(A) **and cDNA from spermatozoa from 4 animals in **(B)**. Similarly, MASA using GATA repeats and cDNA from different somatic tissues **(C) **and spermatozoa is shown in **(D)**. Note the tissue and spermatozoa-specific transcript profiles generated by GACA and GATA repeats. GATA did not detect any transcripts in lung and heart.

After divulgence of GACA/GATA repeats in the mammalian transcriptomes, we pursued with the isolation, cloning and characterization of the transcripts tagged with these repeats in water buffalo *Bubalus bubalis *using varying length of oligos (Additional file 4) to conduct Microsatellite associated sequence amplification (MASA) with cDNA from somatic tissues, gonads and spermatozoa. Briefly, a total of 332 amplicons encompassing 57 from somatic tissues/gonads and 26 from spermatozoa, each from 4 animals were uncovered with GACA repeat (Figure 2A–B and Table 1) and 136 amplicons encompassing 96 from different tissues and 40 from spermatozoa were uncovered with GATA repeat (Figure 2C–D and Table 2).

**Table 1:** Detailed analysis for the MASA identified somatic and spermatozoal transcripts tagged with the GATA repeat motif from water buffalo *Bubalus bubalis*^#^

**(i) mRNA transcripts uncovered from different tissues**								
**Clone ID**	**Accession no.**	**Tissue origin/Size(bp)**	**Homology Status**	**Accession no. of the homologue**	**Gene length**	**Chromo-somal position**	**Position of uncovered transcripts**	**% Homology**

pJC29	DQ289479	Brain/1769	1. *Bos taurus *target 1 genomic scaffold	DP000008	2072671	-	109–395	90%
pJC30	DQ289480	Heart/1768	2. *Bos taurus *lactoferrin (Lf) gene, 5' flanking region exons 1, 2	AY319306	8212	22	123–385	90%
pJC31	DQ289481	Liver/1768						
pJC32	DQ289482	Lung/1812	3. *Bos taurus *T-cell receptor gamma cluster 2 (TCRG2) gene	AY644518	188109	-	109–386	89%
pJC33	DQ289483	Ovary/1767						
pJC34	DQ289484	Spleen/1772	4. *Bos taurus *prion preproprotein (PRNP) and prion-like protein doppel preproprotein gene	AY944236	207929	-	131–395	90%
pJC43	DQ494486	Testis/1767						
pJC55	NS	Kidney/1812	5. *Bos taurus *glutamate-cysteine ligase catalytic subunit (GCLC)	AY957499	447010	-	109–356	91%

pJC44	DQ534902	Kidney/1303	1. Pig DNA sequence from clone CH242-277I8	CR956634	206278	17	104–277	86%
pJC45	DQ534903	Liver/1303	2. Human DNA sequence from clone RP5-1009H6 on chromosome 20 Contains the 3' end of the NFATC2 gene for cytoplasmic calcineurin-dependent (2) nuclear factor of activated T-cells	HS1009H6	89163	20	158–245	90%
pJC56	NS	Ovary/1303					627–774	
pJC57	NS	Spleen/1303						
pJC58	NS	Testis/1303						

pJC35	DQ304116	Heart/1080	1. Human DNA sequence from clone RP11-148E14 on chromosome 10 Contains part of the BTRC gene for beta-transducin repeat	AL627144	36454	10	281–884	94%
pJC36	DQ304117	Liver/1080	2. *Mus musculus *BAC clone RP23-408K9 from chromosome 19	AC140332	206515	19	282–884	90%
pJC37	DQ304118	Lung/1080						
pJC38	DQ494481	Ovary/1080						
pJC39	DQ494482	Spleen/1080						
pJC41	DQ494484	Kidney/1080						
pJC59		Testis/1080						

pJC40	DQ494483	Testis/1043	1. *Bos taurus *prion protein (PRNP) and prion – like protein doppel (PRND) genes, PRNT gene, exons 1 and 2; and putative protein gene	DQ205538	104027	13q17	333–857	89%
			2. *Ovis aries *prion protein gene	U67922	31412	-	333–857	88%
			3. *Odocoileus hemion*us prion protein (prnp) gene	AY330343	65476	-	333–857	87%

pJC42	DQ494485	Kidney/1067	1. *Bos taurus *similar to ring finger protein 149 (LOC506267)	XM_582694	4148	-	398–597	97%
			2. *Canis familiaris *similar to ring finger protein 149	XM_538454	1152	10	403–446	93%

pJC46	DQ534904	Liver/848	1. *B. taurus *mRNA HBGF-1 for acidic fibroblast growth factor (5'end)	X66446	412	-	131–441	97%
pJC60	NS	Spleen/850						
pJC61	NS	Heart/848	2. *Bos taurus *fibroblast growth factor, acidic (FGF1), mRNA	NM_174055	4005	7	131–374	97%
pJC62	NS	Testis/848						
pJC63	NS	Kidney/848	3. *Bubalus bubalis *clone BBMS119 microsatellite sequence	AY779568	452	-	68–285	100%
pJC64	NS	Ovary/848						
pJC65	NS	Lung/858	4. *Homo sapiens *gene for acidic fibroblast growth factor	Z14150	1185	-	256–842	86%

pJC54	DQ834345	Testis/725	1. *Bos taurus *target 1 genomic scaffold	DP000008	2072671	-	139–261	90%
			2. *Bos taurus *bone morphogenetic protein receptor IB gene, exons 8 and 9	AY242067	1253	6	139–261	86%
			3. *Bos taurus *testis expressed sequence 10, mRNA	BC112672	2828	-	174–253	91%

pJC49	DQ534907	Ovary/635	1. Human DNA sequence from clone RP4-752I6 on chromosome 1 Contains the 5' end of the WASF2 gene for WAS protein family	BX293535	71971	1	445–485	91%
pJC66	NS	Kidney/635					555–635	
pJC67	NS	Heart/635						
pJC68	NS	Liver/635						
pJC69	NS	Testis/647						
pJC70	NS	Spleen/635						
			2. Mouse DNA sequence from clone RP23-125F21 on chromosome 4	AL627184	152069	4	555–635	90%

pJC50	DQ534908	Spleen/612	1. *Bos taurus *similar to ankyrin repeat domain 26	XM_580719	1470	21	119–368	86%
pJC71	NS	Ovary/612						

*pJC48	DQ534906	Testis/523	1. *Bos taurus *similar to ankyrin repeat domain 26	XM_580719	1470	21	156–405	86%
pJC72	NS	Ovary/523						

pJC47	DQ534905	Brain/455	1. *Bubalus bubalis *clone 2 minisatellite sequence	AY230133	419	-	43–437	100%
pJC73	NS	Heart/455	2. *Homo sapiens *12 PAC RPCI1-53O8	AC005344	153836	12	125–251	86%
pJC74	NS	Kidney/455						
pJC75	NS	Ovary/455						
pJC76	NS	Spleen/455						
pJC77	NS	Lung/455						
pJC78	NS	Testis/455						
pJC79	NS	Liver/455						

pJC53	DQ834344	Heart/412	1. *Bos taurus *DNA for SINE sequence Bov-tA	X64124	197	-	52–224	89%
			2. *Bos taurus *ABCG2 gene, PKD2 gene and SPP1 gene, clone RPCI42_5K14	AJ871176	171712	6	52–233	86%
			3. *Bos taurus *similar to ataxin-1 ubiquitin-like interacting protein, transcript variant 6	XM_882781	3406	3	54–116	87%

pJC51	DQ534909	Testis/209	1. *Mus musculus *chromosome 1, clone RP23-474A1	AC163217	184175	1	186–209	100%
pJC80	NS	Liver/209						
pJC81	NS	Lung/209	2. *Mus musculus *BAC clone RP24-114C10 from chromosome 13	AC165149	191162	13	188–209	100%
pJC82	NS	Ovary/209						
pJC83	NS	Spleen/209						
pJC84	NS	Kidney/209						
pJC85	NS	Heart/209						

*pJC52	DQ534910	Testis/217	1. *Bos taurus *similar to Ubiquitin-associated protein 1, transcript variant 2	XM_865289	4601	8	7–207	99%
			2. *Canis familiaris *similar to Ubiquitin-associated protein 1, transcript variant 1	XM_531976	2660	11	37–207	94%
			3. Macaca mulatta ubiquitin associated protein 1 (UBAP1),	XM_001089450	4100	15	9–207	90%
			4. *Homo sapiens *ubiquitin associated protein 1 (UBAP1),	NM_016525	2752	9p13.3	9–207	90%

**(ii) mRNA transcripts identified in the spermatozoa**								

**Clone ID**	**Accession no.**	**Size (bp)**	**Homology Status**	**Accession no. of the homologue**	**Gene length**	**Chromosomal position**	**Position of uncovered transcripts**	**% Homology**

pJSC1	DQ789045	1313	▪ Same as pJC44–45 and pJC56–58					
pJSC2	DQ789046	857	▪ Same as pJC46 and pJC60–61					
pJSC3	DQ789047	807	1. *Bubalus bubalis *minisatellite associated amplified segment	AY212951	757	-	16–792	96%
			2. *Bos taurus *similar to non-POU domain containing, octamer-binding	BC105532	2580	-	558–737	90%

pJSC4	DQ789048	789	1. Hippopotamus amphibius DNA, SINE-containing sequence	AB007204	311	-	582–611	100%
			2. *Bos taurus *BTA29 11629 genomic sequence contig containing highly polymorphic single nucleotide sites	DQ404153	18838	29	659–686	100%
			3. Globicephala macrorhynchus DNA, CHR-2 SINE FL type sequence	AB071578	321	-	659–742	88%

pJSC5	DQ789049	844	1. *Bos taurus *similar to zinc finger, DHHC domain	XM_869440	1676	-	217–414	91%
			2. Canis familiaris similar to zinc finger, DHHC domain	XM_846705	1470	-	290–397	83%

pJSC6	DQ834346	797	1. *Homo sapiens *BAC clone RP11-703G6 from 4	AC074349	176467	4	95–401	85%

pJSC7	DQ834347	840	▪ Same as pJC48 and pJC50			-		

pJSC8	DQ845141	635	▪ Same as pJC49 and pJC66–70			-		

pJSC9	DQ845142	507	1. *Bos taurus *prion preproprotein (PRNP) and prion-like protein doppel preproprotein (PRND	AY944236	207929	-	52–339	88%
			2. *Bos taurus *T cell receptor gamma cluster 2 (TCRG2) gene	AY644518	188109	-	52–339	87%
			3. *Capra hircus *sex-specific gonadal PISRT1 mRNA	AF404302	48420	1q43	52–337	87%

pJSC10	DQ845143	516	1. *Bos taurus *similar to Disabled homolog 2	BC111684	805	-	272–443	97%
			2. *Homo sapiens *disabled-2 gene	AF218839S1	2196	5p12–p13	356–507	91%
			3. Pan troglodytes similar to disabled 2 p93	XM_517792	5113	5	356–435	91%

pJSC11	DQ845144	523	▪ Same as pJC48, pJC50 and pJSC6			-		

pJSC12	DQ845145	532	1. Human DNA sequence from clone RP11-790G19 on chromosome 10 Contains the 5' end of the gene for transmembrane receptor Unc5H2, the 3'end of a novel gene and two CpG islands	AL359832	195130	10	394–431	97%

pJSC13	DQ845146	531	1. *Mus musculus *chromosome 15, clone RP24-236A19	AC158973	187091	15	46–327	83%
			2. *Homo sapiens *chromosome 8, clone RP11-1077K19	AC104247	118230	8	133–377	84%

pJSC27		522	▪ Same as pJC48, 50, 71 & 72			-		

pJSC14	DQ904036	455	▪ Same as pJC47 and pJC73–79			-		

pJSC15	DQ904037	392	1. *Mus musculus *BAC clone RP23-136L14 from chromosome 16	AC166171	199601	16	362–398	100%

pJSC16	DQ904038	387	1. *B. taurus *micosatellite DNA, clone BOV1.1.2	Y07736	826	-	160–335	89%
			2. *Bos taurus *BAC CH240-275I24 (Children's Hospital Oakland Research Institute Bovine BAC Library (male)	AC150707	153353	-	120–261	90%

pJSC17	DQ904039	354	1. *Bos taurus *similar to Potassium voltage-gated channel subfamily C member 4 (Voltage-gated potassium channel subunit Kv3.4) (Raw3)	XM_613047	2561	3	33–346	97%

pJSC18	DQ913640	267	1. Zebrafish DNA sequence from clone CH211-222O4 in linkage group 3	BX004760	190220	-	2–28	96%

pJSC19	DQ913641	277	1. *Mus musculus *BAC clone RP23-111N9 from chromosome 7	AC147502	202934	7	165–191	96%

pJSC20	DQ913642	291	1. *Bos taurus *partial ed1 gene for Ectodysplasin 1	BTA300468	9596	Xq22–q24	97–220	91%
			2. *Bos taurus *HIV-1 Tat interactive protein 2 HTATIP2	BC104577	1645	-	97–218	90%
			3. *Bos taurus *similar to C4b-binding protein alpha chain precursor (Proline-rich protein) (PRP)	XM_583188	2960	-	97–216	90%

pJSC21	DQ913643	301	▪ Same as pJC48, pJC50, pJSC6 and pJSc11			-		

pJSC22	DQ913644	273	1. *Ovis aries *5' flanking region of the Jaagsiekte Sheep Retrovirus integration site	AY322397	466	-	91–203	89%
			2. *Bos taurus *similar to NipSnap1 protein	XM_866639	2458	17	100–203	90%
			3. *Bos taurus *lysozyme (LZ) gene	U25810	12039	5q23	118–205	91%

pJSC23	DQ913645	274	1. Human DNA sequence from clone RP11-541N10 on chromosome 10 Contains the 5' end of the SH3MD1 gene for SH3 multiple domains 1, a novel gene and two CpG islands	AL133355	190882	10	103–254	89%

pJC24	DQ913646	269	NA			-		

pJSC25	DQ916743	229	1. *Mus musculus *BAC clone RP23-476B3 from chromosome 7	AC121827	183470	7	1–26	100%

pJSC26	DQ916744	209	▪ Same as pJC51, pJC80–85			-		

^# ^The transcripts uncovered from somatic and gonadal tissues are given in **(i) **whereas spermatozoal transcripts in **(ii)**. All of the GACA-tagged transcripts were submitted to the GenBank and the accession numbers were obtained for each transcript. The analysis carried out for their homologues, size and chromosomal positions is also given. Blast search showed homology of these transcripts with several genes/gene fragments across the species. Notably, only few of them represented by '*' had homology along the length while others showed partial homology.

**Table 2:** Analysis of the MASA uncovered somatic and spermatozoal transcripts tagged with the GATA repeat motifs from water buffalo *Bubalus bubalis*^#^

**(i) Identified from somatic tissues and gonads**				**(ii) Identified from spermatozoa**			
**S.No.**	**Clone ID**	**Accession numbers**	**Origin/Size (in bp)**	**S.No.**	**Clone ID**	**Accession numbers**	**Size (in bp)**

1.	pJC86	EF051520	Kidney/807	1.	pJSC28	EF050082	808
2.	pJC95	NS	Testis/807	2.	pJSC31	EF051516	425
3.	pJC94	NS	Ovary/807	3.	pJSC30	EF050084	414
4.	pJC93	NS	Spleen/821	4.	pJSC32	EF051517	417
5.	pJC96	NS	Liver/807	5.	pJSC33	EF051518	367
6.	pJSC29	EF050083	Spleen/425	6.	pJSC34	EF051519	367
7.	pJC97	NS	Testis/425	7.	pJSC35	NS	277
8.	pJC98	NS	Ovary/425	8.	PJSC36	NS	282
9.	pJC99	NS	Kidney/425	9.	pJSC37	NS	150
10.	pJC100	NS	Liver/425	10.	pJSC38	NS	125
11.	pJC101	NS	Testis/414				
12.	pJC102	NS	Testis/417				
13.	pJC89	EF592585	Testis/376				
14.	pJC103	NS	Ovary/367				
15.	pJC104	NS	Liver/367				
16.	pJC105	NS	Kidney/367				
17.	pJC106	NS	Testis/367				
18.	pJC87	EF592582	Testis/277				
19.	pJC88	EF592583	Testis/282				
20.	pJC107	NS	Ovary/282				
21.	pJC108	NS	Spleen/282				
22.	pJC109	NS	Liver/282				
23.	pJC90	EF592585	Testis/150				
24.	pJC91	EF592586	Testis/125				

^# ^The mRNA transcripts detected in somatic tissues are described in **(i) **whereas spermatozoal transcripts in **(ii)**. Note that these transcripts did not show any homology with genes present in databank.

Cloning and sequencing of the GACA uncovered amplicons identified a total of 14 different transcripts in the somatic tissues and gonads whereas 26 types of transcripts were detected in the spermatozoa of buffaloes (Table 1). Upon subsequent sequence analyses and characterization, we observed that of the 14 tissue-originated transcripts, only 5 were common to all the tissues studied while remaining ones showed tissue-specificity (for details, see Table 1). Of these tissue-specific transcripts, 3 were exclusive to the testis, 1 each for kidney and heart, 1 common for testis and ovary while 9 were absent in the lung. Of the 26 spermatozoal transcripts uncovered, only 6 were shared with somatic tissues whereas remaining 20 were exclusive to the spermatozoal RNA pool (Table 1). Database search revealed that ~80% of the somatic and ~60% of spermatozoal transcripts have significant homologies (>85%) with various coding genes across the species. However, only two of them showed similarity along their entire length (Accession no. DQ534910 and DQ534906), whereas remaining ones were homologous either to the 5'/3' regions or intervening sequences of the characterized genes. Remaining fragments were found to be novel as they showed non-substantial or no homology with the genes present in the Databank. Interestingly, >80% of the homologous genes were found to be involved either in signal transduction or cell-cell interaction pathways whereas remaining ~20% were implicated with several diseases reported in the human. Details of the uncovered GACA-tagged transcripts, their homologous genes and corresponding accession numbers are given in the Table 1.

**Figure 3:**
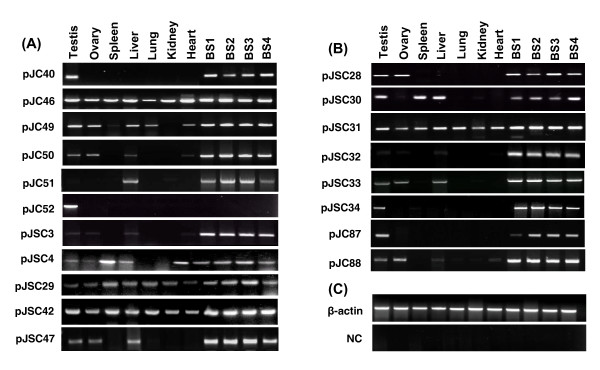
RT-PCR analyses for representative GACA- **(A) **and GATA- **(B) **tagged transcripts using internal primers and cDNA from different somatic tissues, gonads and spermatozoa as templates. The transcript IDs are given on the left and names of the tissues on the top. Quality and quantity of the cDNA samples was normalized **(C) **and genomic contamination in the RNA checked by PCR with β-actin derived primers. Tissue specificities of the transcripts were ascertained on the basis of presence or absence of amplicons using the respective cDNA templates which were further confirmed by real time PCR and Southern blotting.

In contrast to GACA, GATA repeat uncovered fewer transcripts but showed well-defined tissue-specific profiles (Figure 2C). Briefly, a total of 10 types of mRNA transcripts were isolated and characterized from the somatic and gonadal tissues barring lung and heart which were conspicuously devoid of any amplicon (Table 2). These transcripts further exhibited tissue-specificities such that 6 were exclusive to the testis, while remaining 4 common to all the tissues. Also, we identified 10 types of transcripts from the spermatozoa (Figure 2D) which upon characterization were found to be identical to that uncovered from the testis (Table 2). However, other tissues shared only 4 out of 10 spermatozoal transcripts. Further, >90% of these somatic and spermatozoal transcripts showed no homology with any of the genes. The remaining ones were similar to Bovid specific BAC clones, but none of the GATA-tagged transcripts established homology along its entire length. Details of the GATA tagged somatic and spermatozoal transcripts including their accession numbers, origin and size are given in the Table 2. The observed tissue-specific nature of these GACA/GATA tagged transcripts was confirmed by RNA slot-blot hybridizations (not shown) and RT-PCR analyses (Figure 3).

### Sequence polymorphisms detected in GACA/GATA tagged transcripts

Following homology search, we analyzed the sequence organization of these mRNA transcripts at inter-tissue or tissue-spermatozoal levels. The possibility of interclonal sequence variations was ruled out by analyzing 5 recombinant clones each of the GACA/GATA uncovered amplicons.

Our study demonstrated several single nucleotide variations and INDELs in most of the GACA-tagged transcripts. As mentioned above, only 9 transcripts were common amongst tissues and spermatozoa, and the remaining ones restricted to a single tissue or sperm. Of the transcripts detected exclusively in the somatic tissues, a 1.8 kb one (GenBank Accession no. DQ289479–DQ289486) showed insertions of 36 and 4 nucleotides exclusively in the lung, several point nucleotide changes specific to lung/heart or testis/ovary besides a few randomly distributed ones across the tissues (Additional file 5). The transcripts shared by spermatozoa and tissues also brought out some interesting features. For instance, a 1.3 kb transcript (GenBank Accession numbers DQ534902 and DQ534903) showing homology with NFATC2 gene demonstrated the insertion of 10 bp and several single-nucleotide variations exclusively in the spermatozoa (Additional file 6). Next, the point nucleotide changes detected in the transcript similar to HBGF-1 gene (GenBank Accession no. DQ534904) were either common to the tissues, or to spermatozoa (Additional file 7). Similar random deletions, insertions, transversion and transition at various points of 635 bp transcript of WASF2 gene, were detected only in the testis (Additional file 8). Interestingly, Ankyrin repeat domain of 550 bp (GenBank Accession no. DQ534906) showed identical nucleotide sequences both in the testis and sperm, but polymorphism at several points in the ovary. This transcript was not detected in any of the somatic tissues (Additional file 9). Remaining transcripts such as β-transducin repeat and novel 450/209 bp ones showed similar sequences amongst the tissues except few point nucleotide changes (not shown).

Next, we analyzed the GATA-tagged transcripts to explore possible sequence alterations. Though, only 10 GATA-tagged transcripts were uncovered, 4 common across the tissues and 6 restricted to testis/spermatozoa. Sequencing of 5 recombinants of each of the 6 transcripts demonstrated their identical sequences in both the testis and spermatozoa. However, remaining 4 transcripts evinced several single nucleotide deletions, insertions and/or substitutions at many places. Among them was a novel 800 bp transcript (GenBank accession no. EF051520 and EF050082) harboring an insertion of 18 bp at one place exclusively in the spleen, and several point nucleotide changes in sperm/kidney (Additional file 10). Yet another 425 bp transcript (GenBank accession no. EF050083 and EF051516) demonstrated variations such that the point nucleotide changes were either shared between the spermatozoa/gonads or spermatozoa/somatic tissues (Additional file 11). Remaining novel 367 and 282 bp transcripts (Table 2) showed conserved sequences across the tissues and spermatozoa (not shown).

### Copy number status of the uncovered genes

Following the sequence analyses, the copy number of GACA/GATA-tagged gene/gene fragments was calculated by extrapolation of the straight curves obtained in the Real Time PCR assays using 10 fold dilution series of the respective recombinant plasmids. Extrapolation of these standard curves demonstrated the copy number status of the identified gene/gene fragments (data not shown) which varied from 1 to 65 per haploid genome in buffalo.

Out of 32 GACA-tagged transcripts studied, nineteen had single copy; eleven, 2–3; one each, 8–13 and 25–65 copies, respectively (Table 3). Similarly, of the 8 GATA-tagged transcripts, three were single copy and five had 2–5 copies each. Briefly, the copy number varied from 1 for 50%, 2–5 for 45% and 8–65 for remaining 5% for all the GACA/GATA tagged genes/gene fragments.

**Table 3:** Relative quantitative expression and Copy number status of the genes/gene fragments tagged with GACA & GATA repeat motifs, originating from different somatic/gonadal tissues and spermatozoa^#^

**S.N.**	**Clone ID**	**Accession Numbers**	**Relative expression in different tissues (in folds)**							**Relative expression in spermatozoa from four buffaloes**				**Copy number status per haploid genome**	
					
			**Testis**	**Ovary**	**Spleen**	**Liver**	**Lung**	**Kidney**	**Heart**	**SP1**	**SP2**	**SP3**	**SP4**		
**A. For transcripts tagged with GACA repeat motif**														**In blood**	**In germline**

1.	pJC40	DQ494483	194	21	23	17	2	*Cb*	3	274	181	147	239		
2.	pJC42	DQ494485	32	8	2	30	7	51	*Cb*	29	17	21	27	2–3	2–3
3.	pJC52	DQ534910	512	32	34	83	24	60	*Cb*	6	9	5	7	3	3
4.	pJC54	DQ834345	208	28	15	69	3	*Cb*	1	107	119	97	157	1	1
5.	pJC29	DQ289479	15	10	13	45	*Cb*	20	22	49	52	32	45	1	1
6.	pJC35	DQ304116	147	24	51	45	3	*Cb*	3	39	32	51	39	1–2	1–2
7.	pJC44	DQ534902	44	21	17	34	11	25	*Cb*	97	111	97	128	1	1
8.	pJC46	DQ534904	7	6	2	18	3	*Cb*	*3*	14	22	11	12	2	2
9.	pJC47	DQ534905	34	11	14	91	*Cb*	14	2	73	97	87	84	1	1
10.	pJC49	DQ534907	3521	891	330	637	238	*Cb*	630	2896	4792	2702	3326	1	1
11.	pJC51	DQ534909	1663	157	338	2521	3	5	*Cb*	362	239	512	676	1	1
12.	pJC53	DQ834344	17	13	5	29	4	*Cb*	45	18	14	12	16	2	2
13.	pJSC11	DQ845144	4390	1176	664	1097	*Cb*	2	1195	6616	5120	8526	7342	25–65	30–65
14.	pJSC1	DQ789045	46	35	40	36	12	15	*Cb*	36	21	23	27	1	1
16.	pJSC3	DQ789047	156	45	12	87	*Cb*	2	37	1176	724	776	1440	1	1
17.	pJSC4	DQ789048	149	222	376	34	*Cb*	10	6	675	630	608	588	2	2
18.	pJSC5	DQ789049	128	*2*	2	9	2	*Cb*	2	62	47	41	38	2	2
19.	pJSC6	DQ834346	31	21	30	14	*Cb*	13	51	52	97	84	55	1	1
20.	pJSC9	DQ845142	53	4	3	4	3	*Cb*	3	15	14	15	14	2	2
21.	pJSC10	DQ845143	3	3	12	4	*Cb*	14	3	6	4	9	3	3	3
22.	pJSC12	DQ845145	91	6	28	52	2	6	*Cb*	138	97	119	97	1	1
23.	pJSC13	DQ845146	228	181	246	34	2	74	*Cb*	1782	1910	1097	1351	1	1
24.	pJSC15	DQ904037	39	4	26	13	*Cb*	5	2	49	35	45	39	8–13	8–10
25.	pJSC16	DQ904038	31	*Cb*	14	9	2	2	1	117	112	127	118	1	1
26.	pJSC17	DQ904039	27	22	19	15	9	16	*Cb*	14	29	18	20	1	1
27.	pJSC18	DQ913640	18	7	42	28	*Cb*	9	2	34	23	42	23	1	1
28.	pJSC19	DQ913641	85	24	35	28	2	13	*Cb*	69	68	83	52	2	2
29.	pJSC20	DQ913642	89	74	88	81	*Cb*	65	74	81	88	71	82	1	1
30.	pJSC22	DQ913644	*Cb*	4	4	2	2	8	3	75	69	54	61	2–3	2
31.	pJSC23	DQ913645	2	2	12	4	10	2	*Cb*	55	73	41	67	1	1
32.	pJSC24	DQ913646	2	1	12	2	*Cb*	2	1	48	27	42	32	1	1
33.	pJSC25	DQ916743	149	127	104	21	109	64	*Cb*	239	194	195	256	1	1

**B. For transcripts tagged with GATA repeat motif**															

1.	pJSC28	EF050082	114	58	16	5	*Cb*	2	3	51	48	34	42	2–4	2–4
2.	pJSC30	EF050084	169	20	65	48	*Cb*	1	1	168	128	113	137	1	1
3.	pJSC31	EF051516	65	30	23	35	*Cb*	10	5	59	48	53	43	2	2
4.	pJSC32	EF051517	326	33	28	52	*Cb*	8	3	1351	1261	1351	1261	1	1
5.	pJSC33	EF051518	239	57	14	68	2	44	*Cb*	42	68	55	73	3–5	3–5
6.	pJSC34	EF051519	490	78	19	14	*Cb*	37	3	589	510	465	610	2	2
7.	pJC87	EF592582	386	39	14	9	*Cb*	2	3	314	296	357	260	1	1
8.	pJC88	EF592583	134	87	93	102	4	15	*Cb*	201	174	124	145	1–2	1–2

^# ^The expression for gene fragments tagged with GACA repeat is described in **(A) **whereas for GATA-tagged ones in **(B)**. Note the highest expression of most of the GACA-tagged and all GATA-tagged genes in testis and/or spermatozoa.

### Differential expression of the GACA/GATA tagged transcripts

After ascertaining the tissue-specific organizational variations in the GACA/GATA tagged transcripts, their comparative expression profiles were studied to determine possible functional status in the somatic tissues, gonads and spermatozoa. The quantitative expressional analysis was performed for individual mRNA transcript with β-actin as an internal control using SYBR Green assay in Real Time PCR. The results so obtained were substantiated further by expression data from the five additional animals.

**Figure 4:**
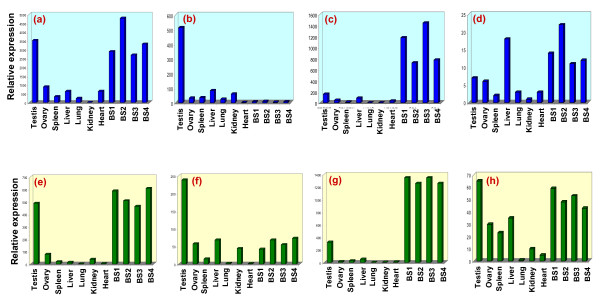
Quantitative expression of representative GACA/GATA-tagged transcripts demonstrating variations among somatic/gonadal tissues and spermatozoa. Four types of expressional profiles were uncovered with GACA; some transcripts with highest expression in testis and spermatozoa e.g. Ankyrin repeat domain **(a)**, few in testis only e.g. Ubap1 **(b)**, few in spermatozoa only e.g. novel pJSC3 **(c)**, and others distributed almost uniformly in all the tissues e.g. HBGF-1 **(d)**. Three types of expressional profiles were observed for GATA-tagged transcripts; some showed highest expression both in testis and spermatozoa e.g. novel pJSC34 **(e)**, few in testis only e.g. novel pJSC33 **(f)**, few others in spermatozoa only e.g. novel pJSC32 **(g)**, and others highest in testis and spermatozoa but with minimal variation in comparison to somatic tissues e.g. novel pJSC31 **(h)**. For details, see table 3 and text.

A total of 32 GACA-tagged transcripts were studied (Table 3) showing differential expression amongst tissues and spermatozoa. The comparative expression of the transcripts detected in the somatic tissues and gonads, evidenced highest expression of ~50% transcripts in the testis and spermatozoa, ~20% in spleen/liver, and remaining ones with uniform expression in all the tissues. Further, the relative expressional studies for the spermatozoal transcripts demonstrated highest expression of ~65% transcripts in testis and/or spermatozoa, 15% in liver/spleen/heart and 20% carrying uniform expression in all the tissues (Table 3). In conclusion, 18 GACA-tagged transcripts demonstrated high or exclusive expression in the testis and/or spermatozoa, encompassing 13 in the spermatozoa followed by testis, 3 in spermatozoa, and 2 specific to the testis. Similarly, 4 transcripts demonstrated highest expression in liver/spleen and 9 showed consistent expression in all of the sources studied. Among all the uncovered transcripts, the highest expression observed was of Ankyrin repeat domain (3400–4390 folds in the testis and 5120–8526 folds in the spermatozoa), followed by of WASF2 gene (3521 folds in testis and 2896 to 4792 folds in spermatozoa) (Figure 4a). The testis specific expression was observed only for 2 transcripts namely Ubap1 and β-transducin repeat (Figure 4b). Others showed either highest/exclusive expression in the spermatozoa (Figure 4c) or uniform expression in all the tissues (Figure 4d).

Following, we pursued the expressional analyses of the GATA-tagged transcripts which demonstrated their highest expression either in testis or spermatozoa or both, compared to that in other tissues (Table 3 & Figure 4e–h). Lung and heart showed almost negligible expression which substantiated the absence of GATA-tagged transcripts in these tissues. Thus, most of the GACA-tagged and all the GATA-tagged transcripts were found to be specific either to the testis or spermatozoa. Details of the expressional analysis of all GACA/GATA tagged transcripts including their accession numbers and relative expression (in folds) have been given in the Table 3.

### Evolutionary status of the entrapped genes

To determine the evolutionary significance of the GACA/GATA tagged transcripts, we studied their conservation across the species by cross-hybridization with genomic DNA from 13 different species (Additional file 12). Among the GACA-tagged transcripts, ~75% were found to be conserved across the 8 species whereas the remaining ones were exclusively detected in the buffaloes or other Bovids. Contrary to this, all the GATA-tagged transcripts showed their cross-hybridization across the species showing differential signal intensities (Additional file 12) suggesting their wider distribution than that of the GACA-tagged ones.

### Chromosomal mapping

**Figure 5:**
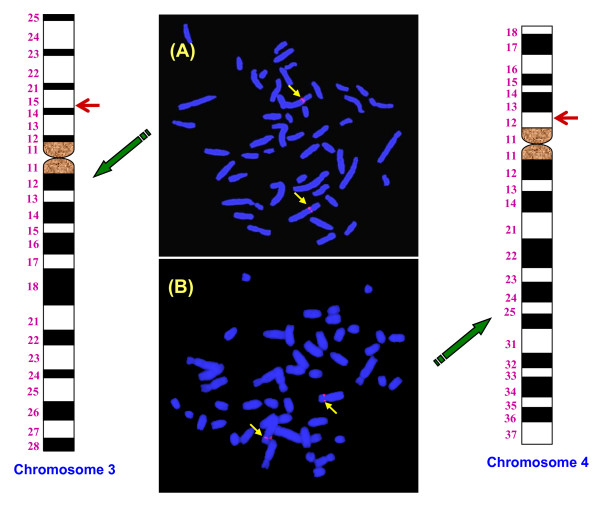
Chromosomal mapping for the candidate Ubap1 gene onto the short arm of metacentric chromosome 3 **(A) **and Ankyrin repeat domain onto the proximal end of the short arm of sub-metacentric chromosome 4 **(B)**. Detailed mapping for these genes with respect to its position on the G-banded ideogram following ISCNDB 2000 is shown in the figure.

Chromosomal mapping employing Fluorescence *in situ *hybridization (FISH) was conducted for two GACA tagged mRNA transcripts, Ankyrin repeat domain-26 and Ubiquitin associated protein 1 (Ubap1). The Ubap1 was mapped onto the short arm of metacentric chromosome 3 (Figure 5A) whereas Ankyrin repeat domain-26 onto the proximal end of the short arm of sub-metacentric chromosome 4 (Figure 5B).

## Discussion

Simple sequence repeats (SSRs) though present ubiquitously, are abundant in the non-coding regions [7,29] which possibly counteract or minimize the ill effects of their frequent shrinkage and expansion causing genetic instability in the coding regions. Presence of such repeats within the transcripts suggests their possible involvement in gene regulation [10,30]. In present study, we established the association of GACA and GATA repeats with the buffalo transcriptome and detected sequence polymorphisms and differential gene expression in several uncovered genes. Moreover, highest expression of GACA/GATA tagged transcripts in testis and/or spermatozoa indicates their crucial roles in male gametogenesis.

Extensive *in silico *analyses demonstrating absence of GACA/GATA repeats in prokaryotes, and presence of a few or no repeats in *S. cerevisiae*, *C. elegans*, *Arabidopsis thaliana *and *Drosophila melanogaster *suggests the accumulation of these repeats in higher eukaryotes during the course of evolution. Further, exploration of GACA/GATA tagged transcriptomes from the lower to higher eukaryotes showing absence of GACA in *Arabidopsis thaliana, Dictyostelium discoideum*, *Drosophila melanogaster *and *C. elegans*, and GATA in *Sus scrofa*, *C. elegans *and *D. discoideum*, and their presence in the respective non-coding regions established their species-specific distribution. These repeats seem to have been acquired in the transcriptomes alongwith the increased genetic complexities in higher eukaryotes. Further, the highlighted sex-chromosomal occurrence and diversity of tagged transcripts suggested the involution of GACA/GATA repeats in regulation of sex-differentiation.

Tandem repeats residing within the coding regions mostly involved in transcription/translation, can also mediate phase variation, and alter the functions and antigenecity of the proteins encoded [31,32]. In the present study, 44 different mRNA transcripts (34 tagged with GACA and 10 with GATA), 23 known and 21 novel ones, were identified using SSRs of GACA/GATA, which can be used as a milestone for contemplating other repeats to establish their combined conclusive significance within and adjacent to the coding regions. However, GACA/GATA tagged transcripts are particularly more important since these are detected in the buffalo spermatozoa as well. Many signaling molecules and transcription factors have been reported in the spermatozoa which pass into the zygotic cytoplasm on fertilization yet ~3000–5000 transcripts remains to be characterized [24,27,28]. The existence of GACA/GATA tagged transcripts in buffalo spermatozoa is the first finding which brightens the involvements of these repeats and tagged transcripts during pre- and post-fertilization events. It also opens up newer vistas offering an opportunity to undertake functional characterization of individual mRNA transcripts during fertilization and embryonic development.

Interestingly, the buffalo transcriptome was found to be enriched with GACA repeat while other species including human were observed to be GATA rich. The primates and cetartiodactyls' genomes are relatively GC poor [33], the GC richness of buffalo genome and transcriptome seem to be unique for its organization and thus for replication timings, genetic recombination, methylation and gene expression [33]. The differential transcript profile uncovered may be explained either towards their diverse functions in somatic tissues, gonads (testis/ovary), and spermatozoa, or specific functions at various stages of development. Absence of GATA-tagged transcripts in lung/heart is anticipated to be their transcriptional quiescence whereas tissue-specific transcripts entailed their exclusive requirement in the respective tissues. Moreover, there are two possible explanations for the detection of 20 of 34 GACA-tagged and 6 of 10 GATA-tagged transcripts in testis or spermatozoa. First, the transcripts could not be picked up in other tissues due to either polymorphic nature of SSRs or much lower number of transcripts, and second, they are transcriptionally dormant in other tissues barring testis/spermatozoa.

DNA sequence variation can contribute to phenotypic variation by affecting the steady-level of mRNA molecules of a particular gene in a given cell or tissue [34]. The tissue- and spermatozoa-specific sequence organizations in transcripts tagged with GACA/GATA repeats substantiated this hypothesis. Some transcripts showed nucleotide changes exclusively in the spermatozoa, few in the testis, whereas other variations were shared only between testis and spermatozoa. These findings may be explicated by silenced state of the representative transcripts in somatic tissues which are active in testis/spermatozoa or vice versa.

Sequence polymorphisms have been shown to regulate the differences in gene expressions, and inter- and intraspecific phenotypic variations in various organisms [35]. The observed sequence polymorphism and expressional variation for the uncovered genes can be explained by this hypothesis. The uniform expression of ~30% GACA-tagged transcripts suggested their consistent necessitate in all the tissues and sperm, whereas ~10% with highest expression in liver or spleen indicated their involvement in hepatocellular and immunological activities, respectively. Similarly, the highest expression of most of the GACA- and GATA-tagged transcripts was observed in testis and/or spermatozoa. Thus, male-specific expression observed herein corroborated with the earlier studies suggesting the involvement of GACA/GATA repeats in sex-differentiation and their predominant roles in spermatogenesis and fertilization.

## Conclusion

Present study suggests that GACA/GATA repeats have been gradually accumulated in the transcriptomes of higher eukaryotes with an increase of their genetic complexities. This work also established the GACA richness of buffalo transcriptome and the existence of GACA/GATA tagged transcripts in spermatozoa. Most interestingly, the exclusive expression of the GACA/GATA-tagged transcripts in the testis and/or spermatozoa substantiated their involvement in various testicular functions. This is a pioneer study exploring the GACA/GATA repeats in buffalo transcriptomes which highlight the possible key functions of these repeats and tagged transcripts in pre- and post-fertilization events. Following this approach, other repeats can be used to excavate further the tagged transcripts in different species for their comparative organization and expression, which would assist resolving the enigma of such simple sequence repeats in the mammalian genome.

## Methods

### Sperm purification and RNA isolation

Fresh ejaculates of buffaloes were obtained from the local dairy farm. Samples were subjected to percoll gradient method to select only motile sperms as described earlier [36]. Total RNA was isolated as described earlier [37]. The RNA was then treated with RNase-free DNase-1 (10 U in 50 mM Tris-HCl, 10 mM MgCl_2_, pH 7.5) and then re-extracted. Final RNA preparations were tested for residual DNA contamination by PCR using primers against β-actin following standard procedures [38].

### Isolation of genomic DNA, total RNA from different tissues and cDNA synthesis

Blood and tissue samples of both the sexes of water buffalo were collected from local slaughterhouse, following the guidelines of Institute's Ethical and Biosafety Committee. Details of the genomic DNA isolation from buffalo and other species used in this study for cross hybridization have been given [39,40]. Total RNA was isolated from all tissues and blood samples from buffaloes using standard protocols [38,40]. The cDNA synthesis was conducted using a commercially available kit (ABI, USA) and confirmed by PCR amplification using a set of bubaline derived β-actin (forward 5' CAGATCATGTTCGAGACCTTCAA 3' and reverse 5'GATGATCTT GATCTTCATTGTGCTG 3') primers.

### Microsatellite associated sequence amplification (MASA)

For conducting microsatellite associated sequence amplification (MASA), 6 sets of oligos based on the GACA and GATA repeats (Additional file 4), were purchased from Microsynth GmbH (Balgach, Switzerland). MASA reactions were performed using cDNA samples as template from different tissues and spermatozoa following standard procedure [38,39]. Annealing temperature for each primer has been given in the Additional file 4. The resultant amplicons were resolved on 2% (w/v) agarose gel using 0.5× TBE buffer.

### Cloning, sequencing and characterization of MASA uncovered amplicons

From the MASA reactions with GACA/GATA repeat motifs, 332 amplicons were uncovered with GACA (Table 1), and 136 amplicons, with GATA (Table 2). These amplicons resolved on the agarose gel were sliced; DNA eluted (Qiagen Gel Extraction kit, Germany) and processed independently for cloning into pGEMT-easy vector (Promega, USA). The resultant recombinant clones were sequenced and sequences were deposited in the GenBank (Table 1 and 2). The recombinant clones were characterized by restriction digestion and slot blot hybridization using labeled buffalo genomic DNA following standard methods [41]. Sequences of the two clones each from every single amplicon were independently subjected to ClustalW alignment to ascertain interclonal variation. Database search was conducted to determine homology of these sequences independently with other entries in the GenBank using default server [42] as described in previous study [41].

### Evolutionary conservation of the uncovered genes/gene fragments

For evolutionary conservation study based on cross hybridization, DNA was extracted from peripheral blood of buffalo *Bubalus bubalis*, cattle *Bos indicus*, sheep *Ovis aries*, goat *Capra hircus*, human *Homo sapiens*, Pigeon *Columba livia*, pig *Sus scrofa*, Baboon *Papio hamadryas*, Bonnet monkey *Macaca radiata*, Langur *Presbytis entellus*, Rhesus monkey *Macaca mulatta*, Lion *Panthera leo*, Tiger *Tigris tigris *following standard protocols [38,40]. Lion and Tiger blood samples were procured with due approval of the competent authorities of the States and Union Government of India. Hybridization of genomic DNA from different sources using recombinant cloned probes was conducted following standard procedures [38,39].

### RNA slot blot analysis, Northern blot, RT-PCR and Southern Blotting

For RNA slot blot analysis, approximately 2 μg of total RNA from different tissues of buffalo in 100 μl of 2 × SSC was slot blotted onto a nylon membrane (Minifold Apparatus, Schleicher & Schuell, Germany) and UV fixed. For positive control, 5 ng of recombinant plasmid, each, was included in the blot(s). For Northern blot analyses, 5–10 μg total RNA was separated on 1% agarose gel containing 4% formaldehyde and transferred to nylon membrane (Amersham Biosciences). Hybridizations were performed under high stringent conditions using standard procedure [38,39]. Individual probes for each fragment was labeled with [^32^P] α-dCTP using rediprime™ II kit (Amersham Pharmacia biotech, USA). In order to confirm the Northern results, internal primers were designed from each fragment (Additional file 4) and RT-PCR was conducted using cDNA from different tissues on their standard thermal profile. The products were transferred to nylon membrane followed by hybridization with [^32^P] α-dCTP labeled respective recombinant clones corresponding to each uncovered fragment using standard procedures [38,40]. Bubaline derived β-actin gene probe and bacterial genomic DNA were used as positive and negative controls, respectively.

### Relative expressional studies using Real Time PCR

For relative expression of MASA uncovered genes/fragments, SYBR green assays were conducted using Real Time PCR (Sequence Detection System, 7000, ABI) for individual fragments using equal amount of cDNA from all the tissues and spermatozoa. Primers for calculating copy number and relative expression for each of the transcripts were designed by "Primer Express Software" (ABI, USA) and have been given in Additional file 4. The cyclic conditions comprise 10 minutes of polymerase activation at 95°C followed by 40 cycles, each at 95°C for 15 seconds and 60°C for 1 minute. Each experiment was repeated three times at different concentration to ensure consistency of the results. The expression level of the genes was calculated using the formula: expression status = (1+E)^-ΔCt^, where E is the efficiency of the PCR and ΔCt is the difference between cycle threshold of the test sample(s) and endogenous control [38,39].

### Metaphase chromosome preparation and Fluorescent in situ hybridization

Approximately, 400 μl of whole blood from normal buffaloes was cultured for chromosome preparation following standard protocols [43,44]. Probes were labeled using Nick Translation Kit from Vysis, (IL, USA), biotin-16-dUTP and detected by FITC-avidin and biotinylated anti-avidin antibody. Two rounds of signal amplification were performed to obtain for the defined signals using standard procedures [43,44]. Chromosome identification and band numbering was done through G-banding following the International System for Chromosome Nomenclature of Domestic Bovids [ISCNDB, 2000].

## Authors' contributions

JS Took the lead, performed the experiments and in-silico analysis, analyzed and interpreted the data, and wrote the manuscript. SP performed the experiments and in-silico analysis, analyzed and interpreted the data. SKProvided the research samples for performing the experiments and intellectual inputs on the manuscript. SA Designed the concept, scrutinized the data analysis, finalized the manuscript and figures and provided overall supervision.

*All of the authors have checked the paper and agreed to submit the same for publication in **'BMC Genomics'***.

## Supplementary Material

Additional file 1 Distribution of the *Bkm *derived GACA/GATA repeats in the non-coding and coding genomes across the species. Chromosomes per haploid genome for respective species are also given in the table. Information on the presence of these repeats in genomes of *Ovis aries *and *Capra hircus *is not available due to their unfinished genomes. Click here for file

Additional file 2 Occurrence of GACA repeats in the mRNA transcripts across the species. Some species such as Archeas, *Arabidopsis thaliana, Zea mays, Dictyostelium discoideum*, *Ovis aries*, *Drosophila melanogaster *and *C. elegans *lacked this repeat. Click here for file

Additional file 3 Occurrence of GATA repeats in the mRNA transcripts across the species. Some species such as Archeas, *Sus scrofa*, *Ovis aries*, *C. familiaris*, *C. elegans *and *D. discoideum *were devoid of this repeat. Click here for file

Additional file 4 List of primers used for identification of the transcripts, RT-PCR, Copy number calculation and Relative expressional studies. The primer IDs alongwith their respective gene accession numbers are also given in the table. Click here for file

Additional file 5 Multiple sequence alignment of GACA-tagged 1.8 kb transcript from different somatic and gonadal tissues. Note the single nucleotide variations throughout the sequence. The variations shared by gonads and somatic tissues are highlighted in red, the ones common to somatic tissues in blue and gonad specific in pink. Note the exclusive major insertions of 36 and 5 bp in lung, highlighted in blue background, which were reconfirmed by sequencing this fragment from 5 different animals. Click here for file

Additional file 6 Multiple nucleotide sequence alignment of GACA-tagged 1.3 kb transcript originating from different tissues and spermatozoa. The sequence from spermatozoa is highlighted in yellow background. The single nucleotide variations spread along the sequence shared by sperm and other tissues are highlighted in pink, and the ones common to tissues in blue. Several variations detected in sperm or testis only is shown in red. Note the exclusive insertion of 10 bp detected in sperm, highlighted in bold red and grey background. Click here for file

Additional file 7 Multiple sequence alignment of GACA-tagged 850 bp transcript originating from different tissues and spermatozoa, homologous to HBGF-1. The sequence from the spermatozoa is highlighted in yellow background. The single nucleotide variations along the sequence but common across the tissues are highlighted in same color (pink or blue). Several variations detected in sperm or testis only are shown in red and the ones exclusive to ovary in blue background. Click here for file

Additional file 8 Multiple sequence alignment of GACA-tagged 635 bp transcript originating from spermatozoa and different tissues, representing WASF2 gene. The sequence from spermatozoa is highlighted in yellow background. Several variations detected in sperm or testis only are shown in red, and that in somatic tissues are in blue color. Note the single nucleotide variations/insertions/deletions along the sequences from different tissues with highest frequency in testis. Click here for file

Additional file 9 Multiple sequence alignment of GACA-tagged 523 bp transcript originating from testis, ovary and spermatozoa only, homologous to Ankyrin repeat domain-26. The sequence from spermatozoa is highlighted in yellow background. Note identical sequences in testis and spermatozoa (highlighted in red) in comparison to ovary (blue). Click here for file

Additional file 10 Multiple sequence alignment of GATA-tagged 800 bp novel transcript originating from different tissues and spermatozoa. Note the single nucleotide variations/INDELS spread throughout the sequence. The variations common to tissues are highlighted in blue color and that shared by sperm in red. Note the exclusive and major insertions of 14 bp in spleen, highlighted in blue background. Click here for file

Additional file 11 Multiple sequence alignment of GATA-tagged 425 bp novel transcript originating from different tissues and spermatozoa. The sequence from spermatozoa is highlighted in yellow background. The variations common to few tissues are highlighted in same color (blue or red). Click here for file

Additional file 12 Cross-hybridization of genomic DNA from different species with the recombinant clones containing GACA (A) and GATA (B) uncovered genes/gene fragments. The names of the species are given on the top, and the autoradiograms for the respective gene/gene fragments on the left. Note the conservation of all the GATA and ~75% GACA uncovered genes across the species whereas remaining GACA-tagged transcripts were specific to buffalo/Bovids. Click here for file
