# Supplementary material for: Organization and differential expression of the GACA/GATA tagged somatic and spermatozoal transcriptomes in Buffalo Bubalus bubalis
Source: BMC Genomics. 2008 Mar 20;9:132. doi: 10.1186/1471-2164-9-132 (PMC2346481; doi:10.1186/1471-2164-9-132)
Supplement: Additional file 1 — Distribution of the Bkm derived GACA/GATA repeats in the non-coding and coding genomes across the species. Chromosomes per haploid genome for respective species are also given in the table. Information on the presence of these repeats in genomes of Ovis aries and Capra hircus is not available due to their unfinished genomes. [file 1471-2164-9-132-S1.pdf]

Additional file 1: Distribution of *Bkm* derived GACA/GATA repeats across the species

S.No.	Species	Chromosome number (Per haploid genome)	(GACA)4		(GATA)4	
			Genome	Transcriptome	Genome	Transcriptome
1.	Archeas	-	-	-	-	-
2.	*Dictyostelium discoideum*	3	+	-	-	-
3.	*C. elegans*	6	+	-	+	-
4.	*Arabidopsis thaliana*	5	+	-	+	+
5.	*Saccharomyces cerevisae*	16	-	-	+	-
6.	*Gallus gallus*	33	+	+	+	+
7.	*Canis familiaris*	39	+	+	+	-
8.	*Drosophila melanogaster*	5	+	-	+	+
9.	*Danio rerio*	25	+	+	+	+
10.	*Sus scrofa*	19	+	+	+	-
11.	*Ovis aries*	27	+	NA	+	NA
12.	*Capra hircus*	29	+	NA	+	NA
13.	*Zea mays*	10	+	-	+	+
14.	*Bos taurus*	30	+	+	+	+
15.	*Bubalus bubalis*	25	+	+	+	+
16.	*Mus musculus*	20	+	+	+	+
17.	*Rattus norvegicus*	21	+	+	+	+
18.	*Homo sapiens*	23	+	+	+	+
