# Supplementary material for: Organization and differential expression of the GACA/GATA tagged somatic and spermatozoal transcriptomes in Buffalo Bubalus bubalis
Source: BMC Genomics. 2008 Mar 20;9:132. doi: 10.1186/1471-2164-9-132 (PMC2346481; doi:10.1186/1471-2164-9-132)
Supplement: Additional file 2 — Occurrence of GACA repeats in the mRNA transcripts across the species. Some species such as Archeas, Arabidopsis thaliana, Zea mays, Dictyostelium discoideum, Ovis aries, Drosophila melanogaster and C. elegans lacked this repeat. [file 1471-2164-9-132-S2.pdf]

Additional file 2: Occurrences of GACA repeats in the transcriptomes across the species

S.N.	Encoding genes/Transcripts	Species	Accession no.	Length	Repeat position
1.	Vascular endothelial growth factor (VEGF)	*Homo sapiens*	NM_001033756	3476	498-513
2.	Oxidised low density lipoprotein (lectin-like) receptor 1 (OLR1)	*Homo sapiens*	NM_002543	2476	1434-1449
3.	Triggering receptor expressed on myeloid cells-like 2 (TREML2)	*Homo sapiens*	NM_024807	3071	2682-2697
4.	Hyaluronan and proteoglycan link protein 2 (HAPLN2)	*Homo sapiens*	NM_021817	1795	111-126
5.	Glycoprotein VI (platelet) (GP6)	*Homo sapiens*	NM_016363	2263	1216-1231
6.	Protein tyrosine phosphatase, non-receptor type	*Homo sapiens*	NM_002832	2805	27-42
7.	Selenoprotein I (SELI)	*Homo sapiens*	NM_033505	8091	2222-2237
8.	Cardiomyopathy associated 1 (CMYA1)	*Homo sapiens*	NM_194293	6451	2188-2203
9.	cAMP responsive element binding protein 3-like 3	*Homo sapiens*	NM_032607	2586	1885-1900<br>1973-1988<br>2007-2022<br>2041-2056<br>2075-2090
10.	UDP-N-acetyl-alpha-D-galacto-samine:polypeptide N-acetylgalactosa- minyl transferase 10	*Mus musculus*	NM_134189	4725	3319-3334
11.	Nuclear receptor subfamily 2, group E, member 3 (Nr2E3)	*Mus musculus*	NM_013708	2018	100-115
12.	Solute carrier family 25 (mitochondrial carnitine/ acylcarnitine translocase), member 20 (Slc25a20)	*Mus musculus*	NM_020520	1784	105-120
13.	Neuropeptide Y receptor Y1 (Npy1r)	*Mus musculus*	NM_010934	2157	1579-1594
14.	Exocyst complex component 3 (Exoc3)	*Mus musculus*	NM_177333	4597	3735-3750
15.	Adrenergic receptor, beta 2 (Adrb2)	*Mus musculus*	NM_007420	2173	1741-1756
16.	Ankyrin repeat domain 43 (Ankrd43)	*Mus musculus*	NM_183173	3504	2707-2722
17.	Sulfotransferase family 1A, phenol-preferring, member 1 (Sult1a1)	*Mus musculus*	NM_133670	1444	940-955
18.	Potassium voltage gated channel, Shaw-related subfamily, member 1 (Kcnc1)	*Mus musculus*	NM_008421	12065	4416-4435
19.	Poly(A)-specific ribonuclease (deade-nylation nuclease)	*Mus musculus*	NM_028761	2903	2698-2713
20.	Leucine rich repeat containing 39 (Lrrc39), transcript variant 1	*Mus musculus*	NM_027321	2775	1069-1084
21.	Mannoside acetylglucosaminyl trans- ferase 3 (Mgat3)	*Mus musculus*	NM_010795	4665	3581-3596
22.	Bone morphogenetic protein 3 (Bmp3)	*Mus musculus*	NM_173404	2282	217-232
23.	Ovo-like 2 (Drosophila) (Ovol2)	*Mus musculus*	NM_026924	1513	1279-1294
24.	Per-pentamer repeat gene (Ppnr)	*Mus musculus*	NM_012022	2857	738-753<br>754-769
25.	Eukaryotic translation initiation factor 2-alpha kinase 2 (Eif2ak2)	*Mus musculus*	NM_011163	4314	2109-2124<br>2129-2144
26.	Ankyrin repeat and SOCS box-containing protein 1	*Mus musculus*	NM_001039126	5971	3384-3399
27	Zinc fingers and homeoboxes protein 2 (Zhx2)	*Mus musculus*	NM_199449	4214	50-65
28.	Nuclear factor of activated T-cells 5 (Nfat5	*Mus musculus*	NM_133957	13040	12461-12476
29.	Adaptor-related protein complex 3, sigma 2	*Mus musculus*	NM_009682	5781	2006-2021
30	Bromodomain containing 1 (Brd1)	*Mus musculus*	NM_001033274	4702	3560-3575<br>3576-3591
31.	Oxysterol binding protein-like 6 (Osbpl6)	*Mus musculus*	NM_145525	8055	7383-7398<br>7399-7414
32.	Forkhead box K1 (Foxk1)	*Mus musculus*	NM_199068	7462	6044-6059
33.	Serine-arginine repressor protein (Srrp)	*Mus musculus*	NM_177774	2940	2128-2143

34.	Coatomer protein complex, subunit gamma 2 (Copg2)	*Mus musculus*	NM_017478	3200	2535-2550
35.	Nudix (nucleoside diphosphate linked moiety X)-type motif 7 (Nudt7)	*Mus musculus*	NM_024446	2795	1348-1363
36.	ATPase, H+ transporting, lysosomal V0 subunit D2	*Mus musculus*	NM_175406	2523	1678-1693
37.	Lymphocyte-activation gene 3 (Lag3)	*Mus musculus*	NM_008479	2003	269-284
38.	Thioredoxin domain containing 9 (Txndc9)	*Mus musculus*	NM_172054	3267	827-842
39.	Interphotoreceptor matrix proteoglycan 1 (Impg1)	*Mus musculus*	NM_022016	3675	3635-3650
40.	Coiled-coil domain containing 65 (Ccdc65)	*Mus musculus*	NM_153518	1880	1764-1779
41.	Phosphoglucomutase 5 (Pgm5)	*Mus musculus*	XM_989276	3082	2002-2017
42.	Mitogen activated protein kinase 14 (Mapk14)	*Rattus norvegicus*	NM_031020	3452	1533-1548
43.	amyloid beta (A4) precursor protein binding, family B, member 2	*Rattus norvegicus*	XM_001077874	5647	5500-5515
44.	Neurofilament triplet H protein (200 kDa neurofilament protein) Neuro-filament heavy polypeptide (NF-H), transcript variant 2	*Rattus norvegicus*	XM_001065919	3985	2923-2954
45.	Kinesin-Like Protein family member (klp-6)	*Rattus norvegicus*	XR_007915	2817	795-825
46.	Eph receptor A6	*Rattus norvegicus*	XM_221595	3704	2590-2605
47.	RNA-binding protein Musashi2-S	*Rattus norvegicus*	XM_001081205	6780	5811-5826
48.	Cardiomyopathy associated 1	*Rattus norvegicus*	XM_001077697	6093	1971-1988
49.	SWI/SNF-related matrix-associated actin-dependent regulator of chromatin subfamily C member 1	*Rattus norvegicus*	XM_001077020	3960	3735-3750
50.	Spastin	*Rattus norvegicus*	XM_001065741	3928	3710-3725
51.	tyrosine phosphatase, non-receptor type 3, transcript variant 1 (Ptpn3)	*Rattus norvegicus*	XM_001055737	4556	3914-3941
52.	WD repeat domain 40A	*Rattus norvegicus*	XM_001059812	3588	2992-3007
53.	Syntaphilin	*Rattus norvegicus*	XM_001060677	4595	2319-2334
54.	ATPase, H transporting, lysosomal V1 subunit B1	*Rattus norvegicus*	XM_001073086	2514	2273-2284
55.	Exocyst complex component 4 (Exo4)	*Rattus norvegicus*	XM_001063828	3269	1005-1044
56.	G protein-coupled receptor 21	*Rattus norvegicus*	XM_001054010	4576	4138-4160
57.	Sterile alpha motif domain containing 10	*Rattus norvegicus*	XM_575305	2489	1903-1923
58.	Actin-binding LIM protein 1	*Rattus norvegicus*	XM_001072903	5482	2422-2445
59.	Heat shock 70kDa protein 12A	*Rattus norvegicus*	XM_001065021	5763	4017-4051
60.	PDZ domain containing 1 (Pdzk10)	*Rattus norvegicus*	NM_031712	2005	1744-1759
61.	Neuropeptide Y receptor Y1 (Npy1r)	*Rattus norvegicus*	NM_001013032	1801	724-746
62.	cAMP responsive element binding protein 3-like 3 (Creb3l3)	*Rattus norvegicus*	NM_001012115	1956	1634-1651
63.	Flotillin 2 (Flot20	*Rattus norvegicus*	NM_031830	2629	1697-1739
64.	Transforming, acidic coiled-coil containing protein 3 (Tacc3)	*Rattus norvegicus*	NM_001004424	2042	1922-1937
65.	Transforming acidic coiled-coil-containing protein 3 (ERIC-1)	*Bos taurus*	XM_582609	2649	2545-2560
66.	TNNI3 interacting kinase	*Bos taurus*	XM_616055	1890	181-196
67.	Cathepsin D (lysosomal aspartyl protease)	*Bos taurus*	XM_863994	1907	1445-1460
68.	Beta-1,3-N-acetylglucosaminyl transferase lunatic fringe	*Bos taurus*	XM_594357	2741	1807-1875

69.	Nuclear protein 1 (Protein p8)	*Bos taurus*	XM_867457	864	289-304
70.	Keratin 13	*Bos taurus*	XM_584070	1615	1490-1505
71.	Raptor	*Bos taurus*	XM_583606	4464	4021-4036
72.	Carbohydrate (keratan sulfate Gal-6) sulfotransferase 1	*Bos taurus*	XM_872521	5309	1911-1926
73.	Transducin-like enhancer protein 3 (ESG3)	*Bos taurus*	XM_872521	5309	4266-4281
74.	Cholecystokinin type A receptor	*Bos taurus*	XM_582625	2784	2619-2634
75.	Heat-shock protein beta-7 (HspB7)	*Bos taurus*	XM_589727	1505	856-871
76.	Vascular endothelial growth factor (VEGF)	*Bos taurus*	NM_174216	1536	12-27
77.	2',3'-cyclic nucleotide 3' phosphodie-sterase (CNP)	*Gallus gallus*	NM_205050	2762	1746-1761
78.	Solute carrier family 29	*Canis familiaris*	NM_001003367	2010	1733-1748
79.	Uroplakin 3B isoform b	*Canis familiaris*	XM_844944	1062	90-105
80.	Apolipoprotein A-IV (APOA4)	*Sus scrofa*	NM_214388	1388	1285-1300
81.	RNA binding motif protein 5 (RBM5)	*Gallus gallus*	NM_001012780	3317	3166-3180
82.	Ras GTPase-activating protein SynGAP (Synaptic-Ras-GTPase-activating protein 1)	*Danio rerio*	XM_685791	1800	508-523
83.	Poly(rC)-binding protein 3	*Danio rerio*	XM_703071	1177	981-996
84.	Striatin, calmodulin binding protein 3	*Danio rerio*	XM_686488	3470	1710-1725
85.	Nicotinamide nucleotide adenylyltrans - ferase 2 (nmnat2)	*Danio rerio*	NM_200004	1620	1047-1062
86.	NMDA receptor-regulated gene 1b (narg1b)	*Danio rerio*	NM_203321	3206	2924-2939
87.	Inhibitor of kappa light polypeptide gene enhancer in B-cells, kinase beta	*Xenopus tropicalis*	NM_001005651	2992	237-252
88.	CASP2 and RIPK1 domain containing adaptor with death domain (cradd)	*Xenopus tropicalis*	NM_001006910	1711	1032-1047<br>1044-1056
