# Supplementary material for: Organization and differential expression of the GACA/GATA tagged somatic and spermatozoal transcriptomes in Buffalo Bubalus bubalis
Source: BMC Genomics. 2008 Mar 20;9:132. doi: 10.1186/1471-2164-9-132 (PMC2346481; doi:10.1186/1471-2164-9-132)
Supplement: Additional file 3 — Occurrence of GATA repeats in the mRNA transcripts across the species. Some species such as Archeas, Sus scrofa, Ovis aries, C. familiaris, C. elegans and D. discoideum were devoid of this repeat. [file 1471-2164-9-132-S3.pdf]

Additional file 3: Occurrences of GATA repeats in the transcriptomes across the species

S.N.	Encoding genes/Transcripts	Species	Accession no.	Length	Repeat position
1.	Sorting nexin 1 (SNX1)	*Homo sapiens*	NM_152826	8077	4827-4869
2.	Ras association (RalGDS/AF-6) and pleckstrin homology domains 1 (RAPH1)	*Homo sapiens*	NM_213589	9615	9356-9374
3.	Ankyrin repeat domain 5 (ANKRD5)	*Homo sapiens*	NM_198798	3775	3463-3536
4.	Peripheral myelin protein 2 (PMP2)	*Homo sapiens*	NM_002677	3579	2330-2372
5.	NK2 transcription factor related, locus 3	*Homo sapiens*	NM_145285	2083	1883-1903
6.	Potassium channel, subfamily K, member 2 (KCNK2)	*Homo sapiens*	NM_001017425	3212	2168-2217
7.	Coiled-coil and C2 domain containing 1B (CC2D1B)	*Homo sapiens*	NM_032449	5639	2913-2933
8.	Origin recognition complex, subunit 6 like (yeast) (ORC6L)	*Homo sapiens*	NM_014321	1647	1395-1449
9.	Glycerophosphodiester phosphordie-sterase domain containing 4 (GDPD4)	*Homo sapiens*	NM_182833	2545	1806-1824
10.	TBP-associated factor 9L	*Homo sapiens*	XM_925841	6660	3192-3232
11.	Kynurenine 3-monooxygenase (kynurenine 3-hydroxylase)	*Homo sapiens*	NM_003679	4992	2086-2152
12.	Solute carrier family 11 (proton-coupled divalent metal ion transporters), member 1 (SLC11A1)	*Homo sapiens*	NM_000578	3865	2402-2422
13.	Regulator of G-protein signalling 5 (RGS5)	*Homo sapiens*	NM_003617	5848	3144-3158
14.	WD repeat domain 72 (WDR72)	*Homo sapiens*	NM_182758	5900	4373-4388
15.	Zinc finger and BTB domain containing 4 (ZBTB4)	*Homo sapiens*	NM_020899	5888	5245-5259
16.	Sialic acid binding Ig-like lectin 1, sialoadhesin (Siglec1)	*Mus musculus*	NM_011426	6875	6818-6872
17.	Simple repeat sequence-containing transcript (Srst)	*Mus musculus*	NM_009276	1263	1080-1132
18.	Extracellular matrix protein 2	*Mus musculus*	NM_001012324	3627	3243-3275
19.	SH3-domain GRB2-like 2 (Sh3gl2)	*Mus musculus*	NM_019535	2817	1724-1784<br>1833-1861<br>1972-2008
20.	Bone morphogenetic protein 8b (Bmp8b)	*Mus musculus*	NM_007559	2942	1940-2008
21.	Choline kinase alpha (Chka)	*Mus musculus*	NM_001025566	3644	1414-1446
22.	Melanocortin 3 receptor (Mc3r)	*Mus musculus*	NM_008561	2624	205-237
23.	Neurotrophic tyrosine kinase, receptor, type 2 (Ntrk2)	*Mus musculus*	NM_008745	7022	2533-2585<br>2662-2688
24.	6-phosphofructo-2-kinase/fructose-2,6-biphosphatase 3 (Pfkfb3)	*Mus musculus*	NM_133232	4991	3515-3565
25.	N-acylsphingosine amidohydrolase (acid ceramidase)-like (Asahl)	*Mus musculus*	NM_025972	1827	1525-1568
26.	Prokineticin receptor 2 (Prokr2)	*Mus musculus*	NM_144944	3655	1767-1786
27	NACHT, LRR and PYD containing protein 12, transcript variant 1 (Nalp12)	*Mus musculus*	XM_973733	3169	2796-2828
28.	Protein phosphatase 1, regulatory (inhibitor) subunit 3E (Ppp1r3e)	*Mus musculus*	XM_989516	3996	350-400
29.	Similar to 60S ribosomal protein L29	*Mus musculus*	XM_975825	1904	33-79
30	RAB11 family interacting protein 1	*Mus musculus*	XM_922410	2326	1980-2006
31.	Myosin VC, transcript variant 9 (Myo5c)	*Mus musculus*	XM_925601	2253	1498-1554
32.	Sorbin and SH3 domain containing 2, transcript variant 1 (Sorbs2)	*Mus musculus*	XM_989416	892	634-698
33.	Nidogen 1 (Nid1)	*Rattus norvegicus*	XM_213954	5220	5180-5220
	Solute carrier family 10, member 2 (Slc10a2)	*Rattus norvegicus*	NM_017222	4269	1725-1779
34.	Myotubularin related protein 2	*Rattus norvegicus*	XM_001068538	3668	3449-3515
35.	Neurotrophic tyrosine kinase, receptor, type 2 (Ntrk2)	*Rattus norvegicus*	NM_012731	4797	3404-3422
36.	Solute carrier organic anion transporter family, member 1b2 (Slco1b2)	*Rattus norvegicus*	NM_031650	3212	2778-2918<br>2951-3005

37.	Cytochrome P450, subfamily 11B, polypeptide 1 (Cyp11b1)	*Rattus norvegicus*	NM_012537	2696	1944-1984
38.	C-reactive protein, pentraxin-related (Crp)	*Rattus norvegicus*	NM_017096	1678	1216-1310
39.	Neurotrophic tyrosine kinase, receptor, type 3 isoform b	*Bos taurus*	XM_585006	1689	1533-1547
40.	LIM and senescent cell antigen-like domains 1	*Gallus gallus*	XM_423884	4725	259-274
41.	Ran-binding protein 2	*Gallus gallus*	XM_423497	1165	2-17
42.	Calpain inhibitor (Calpastatin)	*Gallus gallus*	XM_424713	3551	348-365
43.	Synuclein, beta (SNCB)	*Gallus gallus*	NM_204671	1253	998-1018
44.	Iduronidase, alpha-L- (IDUA)	*Gallus gallus*	NM_001031433	4906	522-537
45.	Poly(rC)-binding protein 3 (Alpha-CP3), transcript variant 2	*Danio rerio*	XM_703071	1177	1053-1161
46.	Monoamine oxidase (mao)	*Danio rerio*	NM_212827	4456	2604-2780
47.	Forkhead box O5 (foxo5)	*Danio rerio*	NM_131085	4547	72-88
48.	Protocadherin 1 gamma b 2 (pcdh1gb2)	*Danio rerio*	NM_001012658	4332	4129-4147
49.	Tumor protein D52-like 2 (tpd52l2	*Danio rerio*	NM_199582	2259	1064-1078
50.	ADP-ribosylation factor 3a (arf3a)	*Danio rerio*	NM_001003441	1475	798-814
51.	CASP2 and RIPK1 domain containing adaptor with death domain (cradd)	*Xenopus tropicalis*	NM_001006910	1711	1000-1032
52.	Phosphodiesterase 6 CG8279-RA (Pde6),	*Drosophila melanogaster*	NM_142112	5136	4076-4099
53.	CXIP1 (CAX INTERACTING PROTEIN 1)	*Arabidopsis thaliana*	NM_115347	806	589-604
54.	NADK1; NAD+ kinase (NADK1)	*Arabidopsis thaliana*	NM_113001	2323	1754-1768
55.	ATP binding / kinase/ protein kinase/ protein serine/threonine kinase/protein-tyrosine kinase (AT4G31170)	*Arabidopsis thaliana*	NM_001036681	1735	1543-1557
56.	Knotted1-like homeodomain protein liguleless3 (lg3)	*Zea Mays*	AF457124	912	713-729
57.	Glutamine synthetase (gs1-2)	*Zea Mays*	AF359511	1505	1345-1361
58.	Ramosa 2 (ra2)	*Zea Mays*	DQ327701	6032	3742-3762
59.	Cinnamoyl-CoA reductase (ccr2)	*Zea Mays*	AY227034	1239	1181-1198
