# Supplementary material for: Organization and differential expression of the GACA/GATA tagged somatic and spermatozoal transcriptomes in Buffalo Bubalus bubalis
Source: BMC Genomics. 2008 Mar 20;9:132. doi: 10.1186/1471-2164-9-132 (PMC2346481; doi:10.1186/1471-2164-9-132)
Supplement: Additional file 4 — List of primers used for identification of the transcripts, RT-PCR, Copy number calculation and Relative expressional studies. The primer IDs alongwith their respective gene accession numbers are also given in the table. [file 1471-2164-9-132-S4.pdf]

Additional file 4: Details of the Oligos used for MASA, RT-PCR, Copy number calculation and Relative expressional studies

S.N.	OLIGO ID	GENE ID	SEQUENCE (5'-3')	ANNEALING TEMP (IN ^{O}C)
**Repeat sequence motifs based on GACA/GATA for identification of tagged transcripts**				
1.	OAT15.2	NA	ACAGACAGACAGACA	54.5
2.	OAT18.2	NA	ACAGACAGACAGACAGACA	56.8
3.	OAT24.2	NA	ACAGACAGACAGACAGACAGACAGACA	60
4.	OAT15.1	NA	ATAGATAGATAGATA	53.6
5.	OAT18.1	NA	ATAGATAGATAGATAGATA	54.9
6.	OAT24.1	NA	ATAGATAGATAGATAGATAGATAGATA	59.5
**Internal Primers Designed for the Identified Genes/Gene Fragments**				
**A. For GACA tagged transcripts**				
1.	JSO466	DQ304116	F ACT GTT TGG TGT TTC TAC TCC TGA C	60
2.	JSO467		R GAA GAC ACA GAT AGG GTG GTT GAT	
3.	JSO468	DQ534905	F CTG TTG CCT TGT AGG GTT TAT ACT G	61
4.	JSO469		R AGA AAC CAG TCA TCA CTG TCA CAA A	
5.	JSO478	DQ289479	F TAA GGC TTC AAA AAC CAG ACC A	64
6.	JSO479		R ACC AAG ACA GAG TTC CCT CCT C	
7.	JSO480	DQ494483	F ACC ACA CAT CAA CTG GAA AAC C	64.5
8.	JSO481		R CCC TGG GAT TAA GAC TCC AAA C	
9.	JSO663	DQ534902	F TCT TTC AGA GGC TAA TTG GGA TT	62
10.	JSO664		R GTT TGA GGG TTA AAT GGG AGA AT	
11.	JSO665	DQ534907	F GGT TTC AGT TCT GAC TCT GCT GT	64
12.	JSO666		R AAT GTG AGC CCC TAA ATA CAC AA	
13.	JSO669	DQ534908	F ATC GAC ACA CTG GCA CAG AC	62
14.	JSO670		R GAC CTT TGA GAC CTC GGA TG	
15.	JSO673	DQ534909	F AGC AAC ACA GGG CAA CAT TT	59
16.	JSO674		R AGG ACA ACA CGT GCA ACA GT	
17.	JSO675	DQ534910	F TAC CAT TGT CCA AGT TCA AA	62
18.	JSO676		R AGT GGC ACG TTC TGG AAT TT	
19.	JSO679	DQ534906	F GAC CTT TGA GAC CTC GGA TG	58
20.	JSO680		R ATC GAC ACA CTG GCA CAG AC	
21.	JSO683	DQ534904	F TGG CAT GTA CAT CAG GTT GC	58
22.	JSO684		R AGG CTA GGG GTA CTC CGT GT	
23.	JSO687	DQ834345	F GCA GGA GAT GTG GGT TCA AT	60
24.	JSO688		R CCC AAA ATC CCA TAT GGT TG	
25.	JSO691	DQ494485	F ACA AAA TCA CGG GTC TCG TC	64
26.	JSO692		R CAG GAG GTG ACT ACC CCA GA	
27.	JSO1221	DQ834344	F CCTTCTTCAGGGGATCTTCC	58
28.	JSO1222		R CATCGCCTTGTGTCAGAGTG	

29.	JSO1223	DQ789047	F ACGGCTGTCCATCTTGTTTC	56.5
30.	JSO1224		R AATGGCAACCCACTCCAATA	
31.	JSO1225	DQ789048	F ACTCACCATCCGTCCTGTTCTA	58
32.	JSO1226		R CCTAGGGTCCAAAACTGTGCTT	
33.	JSO1227	DQ789049	F GGCAGTACTGGTTCTGGATGAC	59
34.	JSO1228		R TTATGAGGACTCAAGCTCAGCA	
35.	JSO1322	DQ834346	F ACACGCAAAACCTGACTTGTGA	58
36.	JSO1323		R TGCCTATGGATGGTTGTTGCTA	
37.	JSO1324	DQ845142	F TATTGATCAGTCCTGGGGGTTC	58.5
38.	JSO1325		R GCTGCTACAGCCTTAGGAATCAA	
39.	JSO1326	DQ845143	F GAAGCATTCCTCGGATGTCA	56.5
40.	JSO1327		R AGACACGTTGAGGGGGAGTT	
41.	JSO1328	DQ845145	F ACACTCTGTGCTTAGGGCTCTG	58
42.	JSO1329		R TCAGAGCCAAGTCTCTCTGACC	
43.	JSO1330	DQ845146	F ATCCTCTTGCCCTTTTTCAGAG	59.5
44.	JSO1331		R ATCTGACCTGCAATCAGAGGAG	
45.	JSO1360	DQ904037	F AAGAAAGTGGTGGACAAGCTGA	58
46.	JSO1361		R CAGACACACAAAGAAGCAAGCA	
47.	JSO1362	DQ904038	F CAGGAGCTCAGCTGGTAAAGAA	56
48.	JSO1363		R CACGAGTGTGGTCTTTGGAAAT	
49.	JSO1364	DQ904039	F TGCATTTGCTGTGATTTAGGTG	58
50.	JSO1365		R TCTTCGGGAGTCAAACAGGATA	
51.	JSO1366	DQ913640	F CAGACAACTGCAACATCACCAT	60
52.	JSO1367		R TTCTGTTCCTGAGTCCCACTTC	
53.	JSO1368	DQ913641	F GAAACAGGGAGAAAGACAAGCA	58
54.	JSO1369		R CAGCCACACACAGAGGTACTGA	
55.	JSO1370	DQ913642	F GCAACCCACTCCAGTGTTCTTA	57
56.	JSO1371		R GAGTGGGAGGTGTTGTGTCAGT	
57.	JSO1372	DQ913644	F CCGTGAGTTACTGATGGACAGG	59
58.	JSO1373		R GACAGACATCAAAAGGCTGACC	
59.	JSO1374	DQ913645	F CAGACAGACACTTGGGGCTA	57
60.	JSO1375		R TGGTGGCTTGTGTCTCACTC	
61.	JSO1376	DQ913646	F AACCCCCACCTCAAGGAGTA	60
62.	JSO1377		R TATGGCCCTTTCTGTTCCTG	
63.	JSO1070	BETA-	F CAG ATC ATG TTC GAG AC TTC AA	59
64.	JSO1071	ACTIN	R GAT GAT CTT CAT TGT GCT	

B. For GATA tagged transcripts

1.	JSO1378	EF050082	F AATCACCACTTTGCAACCACACT	58
2.	JSO1379		R CTTCATACCCAGATGCAGACGAT	
3.	JSO1385	EF050084	F TAGCAGCAGAAATGGACTCAACC	56
4.	JSO1386		R ATACCCAGGGATTGGCACATAGT	
5.	JSO1387	EF051516	F TTTTGTGTCCTGCAGTTCTCTGA	56
6.	JSO1388		R CCTTTGTTCTGGGTACGGTGTAA	
7.	JSO1389	EF051517	F ATCTTGCTTTCTGGAAGACCATC	55
8.	JSO1390		R CAGTGGAAAATCTGTGTGCAATG	
9.	JSO1391	EF051518	F TTAAGCAAGACCCATTCTGTTGC	57
10.	JSO1392		R CAGGAAACATGTCTTTGGTTTTACA	
11.	JSO1393	EF051519	F TTAAGCAAGACCCATTCTGTTGC	55

12.	JSO1394		R CAGGAAACATGTCTTTGGTTTTACA	
13.	JSO1395	EF592582	F GCTACACCACCTTCATGGTCAAC	57
14.	JSO1396		R CATGTGTGTGTGTGTGTGTGTGT	
15.	JSO1397	EF592583	F GATAGATATGGGCTTCCCTGGTG	58
16.	JSO1398		R GCAAGAATACTGGAGTGCGTTTC	

Real Time Primers for Copy Number Calculation and Relative Expressional Studies

A. For GACA identified transcripts

1.	JSR472	DQ304116	F TGA GTG GGA GGA GGA GAA ATA CTT	60
2.	JSR473		R CTA CCC CAG CCC GGG TTA	
3.	JSR474	DQ534905	F GTG AGT TCG TCT CTG GAA ACC AT	60
4.	JSR475		R CAG ACA ATC TTC CCT GCT TTC TG	
5.	JSR482	DQ494483	F GTG GCC CTG TTT GAA AAA CC	60
6.	JSR483		R TGC GGG AGA GTC TTC CAG AT	
7.	JSR484	DQ289479	F CAC CCC TGC AGC TGA TGA A	60
8.	JSR485		R GGA CTG TCC ACT TGC CTT CCT	
9.	JSR605	DQ534907	F TGG GTC ACT CTT CTG CTT CTA ACA	60
10.	JSR606		R CTG CAG AGT CTT TGA GAA TTT TGG	
11.	JSR607	DQ534902	F AGC GCG GTA CTG CGT GTA A	60
12.	JSR608		R TTG CCC TGT GTC AAC CGA TA	
13.	JSR611	DQ534910	F AAG GGT AGG ATG CCC ATT GAG	60
14.	JSR612		R GCA CGT TCT GGA ATT TCC TAA AG	
15.	JSR615	DQ534906	F TCC CTT AGG GAA GCT GCT TCT	60
16.	JSR616		R CGC TCC GAG ATC GAC ACA CAC T	
17.	JSR697	DQ834344	F TGC CAG GCT CCT CTG TCA CT	60
18.	JSR698		R AAG ATC CCC TGA AGA AGG AAA TG	
19.	JSR699	DQ494485	F TGT AAC CGG CCA GTG ACT CA	60
20.	JSR700		R CTC CCT GTG ATG CCA GCT TT	
21.	JSR701	DQ534904	F CTG AGC ATG CAG CCT GTA GGT	60
22.	JSR702		R GTG CAG GCA GGT GTC TAA AGG	
23.	JSR703	DQ534909	F TTA AAA AAC ACA CCT GAG TTG AAA AGT G	60
24.	JSR704		R ATG TGA AGT GCA AGC CTA TTT TAG G	
25.	JSR705	DQ534908	F CAT CCG CTC CCA GAT CGA	60
26.	JSR706		R CCT CCC TTA GGG AAG CTG CTT	
27.	JSR707	DQ834345	F TGG CAA CCC ACT CCC GTA T	60
28.	JSR602		R CAT GTC CGA CTT TTT GTG ACC TT	
29.	JSR619	DQ913644	F TGA GCG ACT GAA CTG AGG TGA A	60
30.	JSR620		R TGA CCG TGA GCC CCA GAA	
31.	JSR625	DQ913645	F TGT GCC TGA GCA GAC ATG TGT	60
32.	JSR626		R TGT TTG CTT TTC TTT GCC TAC AAT	
33.	JSR631	DQ904039	F TGA TTT AGG TGG GAG CCT GAG A	60
34.	JSR632		R GTG CCG TCA CCC CTA GGA	
35.	JSR635	DQ904036	F GTG AGT TCG TCT CTG GAA ACC AT	60
36.	JSR636		R CAG ACA ATC TTC CCT GCT TTC TG	
37.	JSR639	DQ789048	F ACG AGG AGC CTT GTT TGT CTA C	60
38.	JSR640		R TGC CCA CGT GAC TCT TTC TG	
39.	JSR643	DQ789047	F GCA CAT TCA AGG GCT CAT TCATCA	60

40.	JSR644		R GGC TGC ATA GTG TCC CTG TCT	
41.	JSR818	DQ789046	F CGA GGA GCC TTG TTT GTC TAC A	60
42.	JSR819		R TGC CCA CGT GAC TCT TTC TG	
43.	JSR649	DQ845146	F TCC TCT TGC CCT TTT TCA GAG T	60
44.	JSR650		R ACA CCA GTC ATC AGG TAT GAC ATT G	
45.	JSR653	DQ845144	F TCC CTT AGG GAA GCT GCT TCT	60
46.	JSR654		R GAG ATC GAC ACA CTG GCA CAG A	
47.	JSR661	DQ916743	F CAC CCC CCG TGA CTT CCT	60
48.	JSR662		R GCC TGT TGG GCT CCC TTT	
49.	JSR712	DQ904038	F GTA TTC TCG CCT GGA AAT GC	60
50.	JSR711		R CCA ACT CTT ATA GGA CCC ATG GA	
51.	JSR714	DQ845143	F GAA GGA ACC AGA GCT ACT CAC AGT CTA	60
52.	JSR715		R GGG CAC CGA GGA GTC ATG	
53.	JSR716	DQ845142	F GCA TAA GTT TGA GCA AGC TTT GG	60
54.	JSR717		R TAC GAC GTC AGG TAC CCC A	
55.	JSR718	DQ789049	F TCG GCA TAA GCT GGG AGT AAT	60
56.	JSR719		R CAC CTG ATC GCC ATT TCC A	
57.	JSR720	DQ834346	F CAT GGT ACA CGT GAA AGA ATG ACT TC	60
58.	JSR721		R TTC CAA TCT TTG CTT TGC ATT AAA TC	
59.	JSR724	DQ834347	F CAT CCG CTC CGA GAT CGA	60
60.	JSR725		R CCT CCC TTA GGG AAG CTG CTT	
61.	JSR726	DQ913641	F CTG GCC TGG TGT TTA TGT ACG A	60
62.	JSR727		R CAG AAA AGG ACT AGA TAC ACG GAA G	
63.	JSR728	DQ913640	F GAC AAC TGC AAC ATC ACC ATT C	60
64.	JSR729		R AAA GTG CAG CTT TCC AGT CTT GTA	
66.	JSR730	DQ845141	F CCT GCG CAC AGC AGA CAT A	60
	JSR731		R CAG AGT AAC ACA GCA GAG TCA GAA CTG	
67.	JSR732	DQ904037	F TGC CAT GAA GGT TCT AAG AAA GTG	60
68.	JSR733		R TGG TTC TGG GTC TCT CTC TAA CTG TAC A	
69.	JSR734	DQ789045	F TGC AAC AAG TCA CCT GAA AAG ATT	60
70.	JSR735		R GGC CCC GCC GTC TCT	
71.	JSR814	DQ913646	F CAA GAC TCT GAG GTG CAA ATG G	60
72.	JSR815		R CTA AGC CCT CAG GCC CCT AT	
73.	JSR627	DQ913642	F AGT CAG CTG GAG AAG GAA AAG GAA ATG G	60
74.	JSR628		R TGT GAC CCC GTG GAC TGT AG	
75.	JSR1399	DQ845145	F AGG GAA GGC TTC TCA GAG GAA	60
76.	JSR1400		R TGA GAG AGT GGG AAG GGT CTT TC	

B. For GATA uncovered transcripts

1.	JSR876	EF050082	F ATC ACC ACT TTG CAA CCA CAC T	60
2.	JSR877		R TTG CCC GCT AGT TTC ACA TTC	
3.	JSR822	EF050084	F ACT ATG TGC CAA TCC CTG GGT AT	60
4.	JSR823		R TTC CTG AGC TCG TCA CTC CAT	
5.	JSR878	EF051516	F GCA CTT TCC TTT TGT TAT AGT AGC TTG TAG TAT TC	60
6.	JSR879		R TGT TCT GGG TAT GCT GTA AAC CA	
7.	JSR826	EF051517	F AGC AGC AGA AAT AAC TAT AGG GTA TCC T	60
8.	JSR827		R TGT ATG CTG TAT CAC AGT GGA AAA TC	

9.	JSR883	EF051518	F TGC ATC TGA CCT TTA AGA ACA GAA TT	60
10.	JSR884		R AAG ACT CCA CCT ACA ATG TAC TTT CAC T	
11.	JSR1084	EF592582	F TGG CAG AGG CAG TCT GTA TCC	60
12.	JSR1085		R GTG TAG CTG CTT GGC TTG CA	
13.	JSR1087	EF592585	F CCT ACA ATG TAC TTT CAC TTT CAA TCA TC	60
14.	JSR1088		R GCA TCT GAC CTT TAA GAA CAG AAT TTA TG	
15.	JSR1089	EF592583	F AAT ATG GGA GAT CTG GGT TCG A	60
16.	JSR1090		R TGG AGT GCG TTT CCA TGC T	
19.	JSR522	BETA-ACTIN	F TCA CGG AGC GTG GCT ACA G	60
20.	JSR523		R TGG ATG TCA CGG ACG ATT TCC	
