# Supplementary material for: Organization and differential expression of the GACA/GATA tagged somatic and spermatozoal transcriptomes in Buffalo Bubalus bubalis
Source: BMC Genomics. 2008 Mar 20;9:132. doi: 10.1186/1471-2164-9-132 (PMC2346481; doi:10.1186/1471-2164-9-132)
Supplement: Additional file 5 — Multiple sequence alignment of GACA-tagged 1.8 kb transcript from different somatic and gonadal tissues. Note the single nucleotide variations throughout the sequence. The variations shared by gonads and somatic tissues are highlighted in red, the ones common to somatic tissues in blue and gonad specific in pink. Note the exclusive major insertions of 36 and 5 bp in lung, highlighted in blue background, which were reconfirmed by sequencing this fragment from 5 different animals. [file 1471-2164-9-132-S5.pdf]

Additional file 5: Multiple sequence alignment of GACA-tagged 1.8 kb transcript originating from different tissues of buffalo

```
Testis       ----ACAGACAGACAGACAGTGGCAGGGTCTACCCTGAAATCAGTCCAGTTCAGTCACTC 56
Ovary        ----ACAGACAGACAGACAGTGGCAGGGTCTACCCTGAAATCAGTCCAGTTCAGTCACTC 56
Spleen       ACAGACAGACAGACAGACAGTGGCAGGGTCTACCCTGAAATCAGTCCAGTTCAGTCACTC 60
Liver        ----ACAGACAGACAGACAGTGGCAGGGTCTACCCTGAAATCAGTCCAGTTCAGTCACTC 56
Lung         ----ACAGACAGACAGACAGCGGCAGGATCTACCTTGAAATCAGTTCAGTTCAGTCACTC 56
Heart        ----ACAGACAGACAGACAGTGGCAGGGTCTACCCTGAAATCAGTCCAGTTCAGTCACTC 56
Brain        ----ACAGACAGACAGACAGTGGCAGGGTCTACCCTGAGATCAGTCCAGTTCAGTCACTC 56
                 **************** ****** ****** *** ****** **************

Testis       AGTCTTATCCGACTCTTTGCGACCCTATGGACTGCAGCACACCAGGCTTCCCTGTCCATC 116
Ovary        AGTCTTATCCGACTCTTTGCGACCCCATGGACTGCAGCACACCAGGCTTCCCTGTCCATC 116
Spleen       AGTCTTATCCGACTCTTTGCGACCCCATGGACTGCAGCACACCAGGCTTCCCTGTCCATC 120
Liver        AGTCTTATCCGACTCTTTGCGACCCCATGGACTGCAGCACACCAGGCTTCCCTGTCCATC 116
Lung         AGTCGTATCCGACTCTTTGTGACCCCATGGACTGCAGCACGCCAGGCTTTCCTGTCCATC 116
Heart        AGTCTTATCCGACTCTTTGTGACCCCATGGACTGCAGCACGCCAGGCTTCCCTGTCCATC 116
Brain        AGTCTTATCCGACTCTTTGCGACCCCATGGACTGCAGCACGCCAGGCTTCCCTGTCCATC 116
             **** ************** ***** ************** ******** **********

Testis       ------------------------------------GAATCCGTGATGCCATCCAACCAC 140
Ovary        ------------------------------------GAATCCGTGATGCCATCCAACCAC 140
Spleen       ------------------------------------GAATCCGTGATGCCATCCAACCAC 144
Liver        ------------------------------------GAATCCGTGATGCCATCCAACCAC 140
Lung         ACCAACTCCCAGAGTTTATTCAAACTCATGTCCATCGAATCCGTGATGCCATCCAACCAC 176
Heart        ------------------------------------GAATCCGTGATGCCATCCAACCAC 140
Brain        ------------------------------------GAATCCGTGATGCCATCCAACCAC 140
                                                 ************************

Testis       CTCCTCCTCTGTCATCCCCTTCTCCTCCTGCCTTTAATCTTTCCCAGCATCAGGGTATTT 200
Ovary        CTCCTCCTCTGTCATCCCCTTCTCCTCCTGCCTTTAATCTTTCCCAGCATCAGGGTATTT 200
Spleen       CTCCTCCTCTGTCATCCCCTTCTCCTCCTGCCTTTAATCTTTCCCAGCATCAGGGTATTT 204
Liver        CTCCTCCTCTGTCATCCCCTTCTCCTCCTGCCTTTAATCTTTCCCAGCATCAGGGTATTT 200
Lung         TTCCTCCTCTGTCATCCCCTTCTCTTCCTGCCTTTAATCTTTCCCAGCATCAGGGTATTT 236
Heart        CTCCTCCTCTGTCATCCCCTTCTCCTCCTGCCTTTAATCTTTCCCAGCATCAGGGTATTT 200
Brain        CTCCTCCTCTGTCATCCCCTTCTCCTCCTGCCTTTAATCTTTCCCAGCATCAGGCTATTT 200
              *********************** ***************************** *****

Testis       TCAAATGAGTCAGTTCTTCGCATCAGGTGGCCAAAGGATTGGAGTTTCAGCTTCAACACC 260
Ovary        TCAAATGAGTCAGTTCCTCGCATCAGGTGGCCAAAGGATTGGAGTTTCAGCTTCAACACC 260
Spleen       TCAAATGAGTCAGTTCTTCGCATCAGGTGGCCAAAGGATTGGAGTTTCAGCTTCAACACC 264
Liver        TCAAATGAGTCAGTTCTTCGCATCAGGTGGCCAAAGGATTGGAGTTTCAGCTTCAACACC 260
Lung         TCAAATAAGTCAGTTCTTCACATCAGATGGCCAAATGATTGGAGTTTCAGCTTCAGCATC 296
Heart        TCAAATGAGTCAGTTCTTCGCATCAGGTGGCCAAAGGATTGGAGTTTCAGCTTCAACACC 260
Brain        TCAAATGAGTCAGTTCTTCGCATCAGGTGGCCAAAGGATTGGAGTTTCAGCTTCGACACC 260
             ****** ********* ** ****** ******** ******************  ** *

Testis       AGTCCTTCCAATGAATATTCAGGACTTATTTCCTTTAGGATGGACTGGTTAGATCTCCTT 320
Ovary        AGTCCTTCCAATGAATATTCAGGACTTATTTCCTTTAGGATGGACTGGTTAGATCTCCTT 320
Spleen       AGTCCTTCCAATGAATATTCAGGACTTATTTCCTTTAGGATGGACTGGTTAGATCTCCTT 324
Liver        AGTCCTTCCAATGAATATTCAGGACTTATTTCCTTTAGGATGGACTGGTTAGATCTCCTT 320
Lung         AGTTCTTCCAATGAATATTCAGGATTGATTTCGTTTAGGATGGACCGGTTAGATCTCCTT 356
Heart        AGTCCTTCCAATGAATATTCAGGACTTATTTCCTTTAGGATGGACTGGTTAGATCTCCTT 320
Brain        AGTCCTTCCAATGAATATTCAGGACTTATTTCCTTTAGGATGGACTGGTTAGATCTCCTT 320
             *** ******************** * ***** ************ **************

Testis       GCTGTCCAAGGGACTCTCAAGAGTCTTCTCCAACACCACACAACAGTTCAAAAGCACCAA 380
Ovary        GCTGTCCAAGGGACTCTCAAGAGTCTTCTCCAACACCACACAACAGTTCAAAAGCACCAA 380
Spleen       GCTGTCCAAGGGACTCTCAAGAGTCTTCTCCAACACCACACAACAGTTCAAAAGCACCAA 384
Liver        GCTGTCCAAGGGACTCTCAAGAGTCTTCTCCAACACCACACAACAGTTCAAAAGCACCAA 380
Lung         GCTGTCCAAGGGACTCTCAAGAGTCTTCTCCAACACCACACGACAGTTCAAAAGCGCCAG 416
```

```
Heart        GCTGTCCAAGGGACTCTCAAGAGTCTTCTCCAACACCACACGACAGTTCAAAAGCACCAA 380
Brain        GCTGTCCAAGGGACTCTCAAGAGTCTTCTCCAACACCACACAACAGTTCAAAAGCACCAA 380
             ***************************************** ************* ***

Testis       TTCTTCAGTGCTCAGA-ATTCTATTTTTT-GAAATGGGCGTCCCCTATTTCAAAGTGAGA 438
Ovary        TTCTTCAGTGCTCAGA-ATTCTATTTTTT-GAAATGGGCGTCCCCTATTTCAAAGTGAGA 438
Spleen       TTCTTCAGTGCTCAGA-ATTCTATTTTTTTGAAATGGGCGTCCCCTATTTCAAAGTGAGA 443
Liver        TTCTTCAGTGCTCAGA-ATTCTATTTTTTTGAAATGGGCGTCCCCTATTTCAAAGTGAGA 439
Lung         TTCTTCGGTGCTCAGCTATTTTATTTTTTTGAAGTGGGCATCCCCTATTTCAAAGTGAGA 476
Heart        TTCTTCAGTGCTCAGA-ATTTTATTTTTTTGAAATGGGCGTCCCCTATTTCAAAGTGAGA 439
Brain        TTCTTCAGTGCTCAGA-ATTTTATTTTTTTGAAATGGGCGTCCCCTATTTCAAAGTGAGA 439
             ****** ********  *** ******** *** ***** ********************

Testis       CCAGGGCCCAAGGGCTGAGAGGATCCTCCACCTACCTCTAAGGCTTCAAAAACCAGACCA 498
Ovary        CCAGGGCCCAAGGGCTGAGAGGATCCTCCACCTACCTCTAAGGCTTCAAAAACCAGACCA 498
Spleen       CCAGGGCCCAAGGGCTGAGAGGATCCTCCACCTACCTCTAAGGCTTCAAAAACCAGACCA 503
Liver        CCAGGGCCCAAGGGCTGAGAGGATCCTCCACCTACCTCTAAGGCTTCAAAAACCAGACCA 499
Lung         CCAGGGCCCAAGGGCTGAGGGGATCCTTTGCCTAGCTCTAGGGCTTCAAAAACCAGACAA 536
Heart        CCAGGGCCCAAGGGCCGAGAGGATCCTCCACCTACCTCTAAGGCTTCAAAAACCAGACCA 499
Brain        CCAGGGCCCAAGGGCCGAGAGGATCCTCCACCTACCTCTAAGGCTTCAAAAACCAGACCA 499
             *************** *** *******   **** ***** ***************** *

Testis       GAGAAATAGGATCCCAGAGAGCAGGAATTCATTCACCCCTGCAGCTGATGAAATTAGAGC 558
Ovary        GAGAAATAGGATCCCAGAGAGCAGGAATTCATTCACCCCTGCAGCTGATGAAATTAGAGC 558
Spleen       GAGAAATAGGATCCCAGAGAGCAGGAATTCATTCACCCCTGCAGCTGATGAAATTAGAGC 563
Liver        GAGAAATAGGATCCCAGAGAGCAGGAATTCATTCACCCCTGCAGCTGATGAAATTAGAGC 559
Lung         GAGAAAGAGGATCCCAGAGAGCAGGAATTCATTCACCCCTGCAGCTGATGAAATTAGAGC 596
Heart        GAGAAATAGGATCCCAGAGAGCAGGAATTCACTCACCCCTGCAGCTGATGAAATTAGAGC 559
Brain        GAGAAATAGGATCCCAGAGAGCAGGAATTCACTCACCCCTGCAGCTGATGAAATTAGAGC 559
             ****** ************************ ****************************

Testis       CTGCATGTGGATGTCAATCCAGGAAGGCAAGTGGAGAGTCCCATCCTGGGGACTCCCTTC 618
Ovary        CTGCATGTGGATGTCAATCCAGGAAGGCAAGTGGAGAGTCCCATCCTGGGGACTCCCTTC 618
Spleen       CTGCATGTGGATGTCAATCCAGGAAGGCAAGTGGAGAGTCCCATCCTGGGGACTCCCTTC 623
Liver        CTGCATGTGGATGTCAATCCAGGAAGGCAAGTGGAGAGTCCCATCCTGGGGACTCCCTTC 619
Lung         CTGCGTGTGGATGTCAATCCAGGAAGGCAAGTGGAGAGTCCCATCCTGGGGACTCCCTTC 656
Heart        CTGCATGTGGATGTCAATCCAGGAAGGCAAGTGGAGAGTCCCATCCTGGGGACTCCCTTC 619
Brain        CTGCATGTGGATGTCAATCCAGGAAGGCAAGTGGAGAGTCCCATCCTGGGGACTCCCTTC 619
             **** *******************************************************

Testis       GGTTTCTTAGCAAGGTCCAAGGGAGGGAACTACCAAAGGTGGGCTTAGGAGAGGAGCCCA 678
Ovary        GGTTTCTTAGCAAGGTCCAAGGGAGGGAACTACCAAAGGTGGGCTTAGGAGAGGAGCCCA 678
Spleen       GGTTTCTTAGCAAGGTCCAAGGGAGGGAACTACCAAAGGTGGGCTTAGGAGAGGAGCCCA 683
Liver        GGTTTCTTAGCAAGGTCCAAGGGAGGGAACTACCAAAGGTGGGCTTAGGAGAGGAGCCCA 679
Lung         AGTTTCTTAGCAAGGTCCAAGGGAGGGAGCTACCAAAGGTGGGCTCAGGAGAGGAGCCCA 716
Heart        GGTTTCTTAGCAAGGTCCAAGGGAGGGAACTACCAAAGGTGGGCTCAGGATAGGAGCCCA 679
Brain        GGTTTCTTAGCAAGGTCCAAGGGAGGGAACTACCAAAGGTGGGCTTAGGAGAGGAGCCCA 679
              *************************** **************** **** *********

Testis       CCTGGCCACAGTGGGTCTTCCCTCCCATGTTTCAGTACCAGAAACGTAAGGCAAGCGCGA 738
Ovary        CCTGGCCACAGTGGGTCTTCCCTCCCATGTTTCAGTACCAGAAACGTAAGGCAAGCGCGA 738
Spleen       CCTGGCCACAGTGGGTCTTCCCTCCCATGTTTCAGTACCAGAAACGTAAGGCAAGCGCGA 743
Liver        CCTGGCCACAGTGGGTCTTCCCTCCCATGTTTCAGTACCAGAAACGTAAGGCAAGCGCGA 739
Lung         CCTGGCCGCAGTTGGTCTTCCCTCCCATGTTTCAGTACCAGAAACGTAAGGCAAGCGCGA 776
Heart        CCTGGCCACAGTGGGCCTTCCCTCCCATGTTTTAGTACCAGAAACGTAAGGCAAGCGCGA 739
Brain        CCTGGCCACAGTGGGTCTTCCCTCCCATGTTTCAGTACCAGAAACGTAAGGCAAGCGCGA 739
             ******* **** ** **************** ***************************

Testis       TGAAAAAGAGAGTGAGCCAGGCATTCAAGCAAAGAAAGATTTATTATAGGAAGAAAGAAA 798
Ovary        TGAAAAAGAGAGTGAGCCAGGCATTCAAGCAGAGAAAGATTTATTATAGGAAGAAAGAAA 798
Spleen       TGAAAAAGAGAGTGAGCCAGGCATTCAAGCAGAGAAAGATTTATTATAGGAAGAAAGAAA 803
Liver        TGAAAAAGAGAGTGAGCCAGGCATTCAAGCAGAGAAAGATTTATTATAGGAAGAAAGAAA 799
Lung         TGAAAAAGAGAGTGAGCCAGGCATTCAAGCAGAGAAAGATTTATTATAGGAAGAAAGAAA 836
```

```
Heart           TGAAAAAGAGAGTGAGCCAGGCATTCAAGCAGAGAAAGATTTATTATAGGAAGAAAGAAA 799
Brain           TGAAAAAGAGAGTGAGCCAGGCATTCAAGCAGAGAAAGATTTATTATAGGAAGAAAGAAA 799
                ******************************* ****************************

Testis          GAAA----TTGACTATAGAGAGAAACCAGTGCTCTATTTTTACAGCCAACCCTTCTTATA 854
Ovary           GAAA----TTGGCTATAGAGAGAAACCAGTGCTCTATTTTTACAGCCAACCCTTCTTATA 854
Spleen          GAAA----TTGGCTATAGAGAGAAACCAGTGCTCTATTTTTACAGCCAACCCTTCTTATA 859
Liver           GAAA----TTGGCTATAGAGAGAAACCAGTGCTCTATTTTTACAGCCAACCCTTCTTATA 855
Lung            GAAAGTGACTGGCTATTGAGAGAAACCAGTGCTCTCTTTTTACAGCCAACCCTTCTTATA 896
Heart           GAAA----TTGGCTATAGAGAGAAACCAGTGCTCTATTTTTACAGCCAACCCTTCTTATA 855
Brain           GAAA----TTGGCTATAGAGAGAAACCAGTGCTCTATTTTTACAGCCAACCCTTCTTATA 855
                ****     ** **** ****************** ************************

Testis          CCCTATCGATGGGAAATTGTGCCCTGGAGGAAGGGGGCTTCAGTTTATCTTTAGCCCACA 914
Ovary           CCCTATCGATGGGAAATTGTGCCCTGGAGGAAGGGGGCTTCAGTTTATCTTTAGCCCACA 914
Spleen          CCCTATCGATGGGAAATTGTGCCCTGGAGGAAGGGGGCTTCAGTTTATCTTTAGCCCACA 919
Liver           CCCTATCGATGGGAAATTGTGCCCTGGAGGAAGGGGGCTTCAGTTTATCTTTAGCCCACA 915
Lung            CCCTATCGATGGGAAATTGTGCCCTGGAGGAAGGGGGCCTCAGTTTATCTTTAGCCTGCA 956
Heart           CCCTATCGATGGGAAATTGTGCCCTGGAGGAAGGGGGCTTCAGTTTATCTTTAGCCCTCA 915
Brain           CCCTATCGATGGGAAATTGTGCCCTGGAGGAAGGGGGCTTCAGTTTATCTTTAGCCCACA 915
                ************************************* ******************  **

Testis          GCTGGGGTCTTATGGTCACTTAGTCGAAGGGGTCTCACAGTCGGGTGGTCACATAGTCTT 974
Ovary           GCTGGGGTCTTATGGTCACTTAGTCGAAGGGGTCTCACAGTCGGGTGGTCACATAGTCTT 974
Spleen          GCTGGGGTCTTATGGTCACTTAGTCGAAGGGGTCTCACAGTCGGGTGGTCACATAGTCTT 979
Liver           GCTGGGGTCTTATGGTCACTTAGTCGAAGGGGTCTCACAGTCGGGTGGTCACATAGTCTT 975
Lung            GCTGGGGTCTTATGGTCACTTAGTCAAAGGGGCCTCACAGTCGGGTGGTCACGTAGTCTT 1016
Heart           GCTGGGGTCTTATGGTCACTTAGTTGAAGGGGTCTCACAGTCGGGTGGTCACATAGTCTT 975
Brain           GCTGGGGTCTTATGGTCACTTAGTCGAAGGGGTCTCACAGTCGGGTGGTCACATAGTCTT 975
                ************************  ****** ******************* *******

Testis          GAGAAACTGGCACCAGCAGG-AAAAACAGGATATCGCCTTAAGGAAGGTACAGTCTGCCC 1033
Ovary           GAGAAACTGGCACCAGCAGG-AAAAACAGGATATCGCCTTAAGGAAGGTACAGTCTGCCC 1033
Spleen          GAGAAACTGGCACCAGCAGG-AAAAACAGGATATCGCCTTAAGGAAGGTACAGTCTGCCC 1038
Liver           GAGAAGCTGGCACCAGCAGG-AAAAACAGGATATCGCCTTAAGGAAGGTACAGTCTGCCC 1034
Lung            GAGAAACTGGCACCAGCAGGGAAAAACAGGATATCGCCTTAAGGTAGGTACAGTCTGCCC 1076
Heart           GAGAAACTGACACCAGCAGG-AAAAACAGGATATCACCTTAAGGAAGGTACAGTCTGCCC 1034
Brain           GAGAAACTGGCACCAGCAGG-AAAAACAGGATATCGCCTTAAGGAAGGTACAGTCTGCCC 1034
                ***** *** ********** ************** ******** ***************

Testis          ACCAGGGCAATGTGGCCACTGTGACTTCATCCCTTGTTTGTCAGGT-ACCACAATGGGTC 1092
Ovary           ACCAGGGCAATGTGGCCACTGTGACTTCATCCCTTGTTTGTCAGGT-ACCACAATGGGTC 1092
Spleen          ACCAGGGCAATGTGGCCACTGTGACTTCATCCCTTGTTTGTCAGGT-ACCACAATGGGTC 1097
Liver           ACCAGGGCAATGTGGCCACTGTGACCTCATCCCTTGTTTGTCAGGT-ACCACAATGGGTC 1093
Lung            ATCAGGGCAATGTGGCCACTGTGACTTCATCCCTTGTTTGTCAGGTCACCACAGTGGGCC 1136
Heart           ACCAGGGCAATGTGGCCACTGTGACTTCATCCCTTGTTTGTCAGGT-ACCACAATGGGCC 1093
Brain           ACCAGGGCAATGTGGCCACTGTGACTTCATCCCTTGTTTGTCAGGT-ACCACAATGGGTC 1093
                * *********************** ******************** ****** **** *

Testis          ATATTTTAACAATACGCTATGAGCCCCTTGAGAACTGTAGCATGTGCAATGTTTTTCTAA 1152
Ovary           ATATTTTAACAATACGCTATGAGCCCCTTGAGAACTGTAGCATGTGCAATGTTTTTCTAA 1152
Spleen          ATATTTTAACAATACGCTATGAGCCCCTTGAGAACTGTAGCATGTGCAATGTTTTTCTAA 1157
Liver           ATATTTTAACAATACGCTATGAGCCCCTTGAGAACTGTAGCATGTGCAATGTTTTTCTAA 1153
Lung            ATATTTTAACAATACGCTATGAGCCCCTTGAGAACTGTGGCATGTGCAATGTTTTTCTAA 1196
Heart           ATATTTTAACAATACACTATGAGCCCCTTGAGAACTGTAGCATGTGCAATGTTTTTCTAA 1153
Brain           ATATTTTAACAATACGCTATGAGCCCCTTGAGAACTGTAGCATGTGCAATGTTTTTCTAA 1153
                *************** ********************** *********************

Testis          GTTGGATCCCATCACAGGGACCTATACATACATGTTCACACTTATTATGGGGGGG-CCTC 1211
Ovary           GTTGGATCCCATCACAGGGACCTATACATACATGTTCACACTTATTATGGGGGGG-CCTC 1211
Spleen          GTTGGATCCCATCACAGAGACCTATACATACATGTTCACACTTATTATGGGGGGG-CCTC 1216
Liver           GTTGGATCCCATCACAGAGACCTATACATACATGTTCACACTTATTATGGGGGGG-CCTC 1212
Lung            GTTGGATCCCATTGCAGAGGCCTATAGGTACATGTTCACACTTATTATGGGGGGG-CCTC 1255
```

```
Heart       GTTGGATCCCATCGCAGAGGACTATACATACATGTTCACACTTATTATGGGGGGG-CCTC 1212
Brain       GTTGGATCCCATCACAGAGACCTATACATACATGTTCACACTTATTATGGGGGGGGCCTC 1213
            ************  *** *  *****  *************************** ****

Testis      CCTTTTTGACCTCCAAGAAGCCTTCCTGCACATGTGCACACAGGGAAGTCTTCCTTAACC 1271
Ovary       CCTTTTTGACCTCCAAGAAGCCTTCCTGCACATGTGCACACAGGGAAGTCTTCCTTAACC 1271
Spleen      CCTTTTTGACCTCCAAGAAGCCTTCCTGCACATGTGCACACAGGGAAGTCTTCCTTAACC 1276
Liver       CCTTTTTGACCTCCAAGAAGCCTTCCTGCACATGTGCACACAGGGAAGTCTTCCTTAACC 1272
Lung        CCTTTTTGACCTCCAAGAAGCCTTCCTGAACATGTGCACACAGGGAAGTCTTCCTTAACC 1315
Heart       CCTTTTTGACCTCCAAGAAGCCTTCCTGCACATGTGCACACAGGGAAGTCTTCCTTAACC 1272
Brain       CCTTTTTGACCTCCAAGAAGCCTTCCTGCACATGTGCACATAGGGAAGTCTTCCTTAACC 1273
            **************************** *********** *******************

Testis      TCAGGAGTGGGCACCTTATCTCTGCTTCAGCAGAGCTCAACTTTTGCCACTAACTTTGTC 1331
Ovary       TCAGGAGTGGGCACCTTATCTCTGCTTCAGCAGAGCTCAACTTTTGCCACTAACTTTGTC 1331
Spleen      TCAGGAGTGGGCACCTTATCTCTGCTTCAGCAGAGCTCAACTTTTGCCACTAACTTTGTC 1336
Liver       TCAGGAGTGGGCACCTTATCTCTGCTTCAGCAGAGCTCAACTTTTGCCACTAACTTTGTC 1332
Lung        TCAGGAGTGGGCACCTTATCTCTGCTTCAGCAGAGCTCAACTTTTGCCACTAACTTTGTC 1375
Heart       CCAGGAGTGGGCACCTTATCTCTGCTTCAGCAGAGCTCAACTTTTGCCACTAACTTTGTC 1332
Brain       TCAGGAGTGGGCACCTTATCTCTGCTTCAGCAGAGCTCAACTTTTGCCACTAACTTTGTC 1333
             ***********************************************************

Testis      CTTGGAATGAGAACAAAGCTTGAATTTTATTCCACCTGACAAATGCCACGTGTCCAGCCC 1391
Ovary       CTTGGAATGAGAACAAAGCTTGAATTTTATTCCACCTGACAAATGCCACGTGTCCAGCCC 1391
Spleen      CTTGGAATGAGAACAAAGCTTGAATTTTATTCCACCTGACAAATGCCACGTGTCCAGCCC 1396
Liver       CTTGGAATGAGAACAAAGCTTGAATTTTATTCCACCTGACAAATGCCACGTGTCCAGCCC 1392
Lung        CTTCGAGTGAGAACAAAGCTTGAATTTTATTCCACCTGACAAATACCAGGTGTCCAGCCC 1435
Heart       CTTGGAATGAGAACAAAGCTTGAATTTTATTCCACCTGACAAATGCCACGTGTCCAGCCC 1392
Brain       CTTGGAATGAGAACAAAGCTTGAATTTTATTCCACCTGACAAATGCCACGTGTCCAGCCC 1393
            *** ** ************************************* *** ***********

Testis      AGAGGCCCACTGTTGCCTAC-TCACCAGAACCCAGGTCGGTACCACTTCTGGGGACCCAC 1450
Ovary       AGAGGCCCACTGTTGCCTAC-TCACCAGAACCCAGGTCGGTACCACTTCTGGGGACCCAC 1450
Spleen      AGAGGCCCACTGTTGCCTAC-TCACCAGAACCCAGGTCGGTACCACTTCTGGGGACCCAC 1455
Liver       AGAGGCCCACTGTTGCCTAC-TCACCAGAACCCAGGTCGGTACCACTTCTGGGGACCCAC 1451
Lung        AGAGGCCCACTGTTGCCTACCTCACTAGAATCCGGGTCGGTACCACTTCTGGGGACCCAC 1495
Heart       AGAGGCCCACTGTTGCCTAC-TCACCAGAACCCGGGTCGGTACCACTTCTGGGGACCCAC 1451
Brain       AGAGGCCCACTGTTGCCTAC-TCACCAGAACCCAGGTCGGTACCACTTCTGGGGACCCAC 1452
            ******************** **** **** ** **************************

Testis      CCCTAACAGAAGCACAGGACCTGGAAAGGCAGGAGGGGCTGCAGCTTCAGGCCTGACCTC 1510
Ovary       CCCTAACAGAAGCACAGGACCTGGAAAGGCAGGAGGGGCTGCAGCTTCAGGCCTGACCTC 1510
Spleen      CCCTAACAGAAGCACAGGACCTGGAAAGGCAGGAGGGGCTGCAGCTTCAGGCCTGACCTC 1515
Liver       CCCTAACAGAAGCACAGGACCTGGAAAGGCAGGAGGGGCTGCAGCTTCAGGCCTGACCTC 1511
Lung        CCCTAACAGAAGCACAGGACCTGGAAAGGCAGGAGAGGCTGCAGCTTCAGGCCTGGCCTC 1555
Heart       CCCTAACAGAAGCACAGGACCTGGAAAGGCAGGAGGGGCTGCAGCTTCAGGCCTGACCTC 1511
Brain       CCCTAACAGAAGCACAGGACCTGGAAAGGCAGGAGGGGCTGCAGCTTCAGGCCTGACCTC 1512
            *********************************** ******************* ****

Testis      CCATCCCTGTGGCCTTAGGTGAAGCTGAGGCTCCTGCCACTGCCTTGTCTGTGGAGCACG 1570
Ovary       CCATCCCTGTGGCCTTAGGTGAAGCTGAGGCTCCTGCCACTGCCTTGTCTGTGGAGCACG 1570
Spleen      CCATCCCTGTGGCCTTAGGTGAAGCTGAGGCTCCTGCCACTGCCTTGTCTGTGGAGCACG 1575
Liver       CCATCCCTGTGGCCTTAGGTGAAGCTGAGGCTCCTGCCACTGCCTTGTCTGTGGAGCACG 1571
Lung        CCATCCCTGTGGCCTTAGGTGAAGCTGAGGCTCTTGCCACTGCCTTGTCTGTGGAGCACG 1615
Heart       CCATCCCTGTGGCCTTAGGTGAAGCTGAGGCTCCTGCCACTGCCTTGTCTGTGGAGCACA 1571
Brain       CCATCCCTGTGGCCTTAGGTGAAGCTGAGGCTCCTGCCACTGCCTTGTCTGTGGAGCACG 1572
            ********************************* *************************

Testis      GCTGCACAATGCCTTTTCTCTCTCCTGGAACTGGTCATCGTTCTGCAGAGCTGGGTCCCC 1630
Ovary       GCTGCACAATGCCTTTTCTCTCTCCTGGAACTGGTCATCGTTCTGCAGAGCTGGGTCCCC 1630
Spleen      GCTGCACAATGCCTTTTCTCTCTCCTGGAACTGGTCATCGTTCTGCAGAGCTGGGTCCCC 1635
Liver       GCTGCACAATGCCTTTTCTCTCTCCTGGAACTGGTCATCGTTCTGCAGAGCTGGGTCCCC 1631
Lung        GCTGTATGACGCCTTTTCTCTCTCCTGGAATTGGTCATCGTTCTGCCCAGCTGGGTCCCC 1675
```

```
Heart       GCTGCACAATGCCTTTTCTCTCTCCTGGAACTGGTCATCGTTCTGCAGAGCTGGGTCCCC 1631
Brain       GCTGCACAATGCCTTTTCTCTCTCCTGGAACTGGTCATCGTTCTGCAGAGCTGGGTCCCC 1632
            **** *  * ******************** ***************  ************

Testis      TCAGCCAAGGAGGAGGGAACTCTGTCTTGGTGGAGGAAGAAGATGAGTCTTTCTCATCCT 1690
Ovary       TCAGCCAAGGAGGAGGGAACTCTGTCTTGGTGGAGGAAGAAGATGAGTCTTTCTCATCCT 1690
Spleen      TCAGCCAAGGAGGAGGGAACTCTGTCTTGGTGGAGGAAGAAGATGAGTCTTTCTCATCCT 1695
Liver       TCAGCCAAGGAGGAGGGAACTCTGTCTTGGTGGAGGAAGAAGATGAGTCTTTCTCATCCT 1691
Lung        TCGGCCAAGGAGGAGGGAACCCTGTCTTGGTGAAGGAAGAAGATGAGTCTTTCTCATCCT 1735
Heart       TCAGCCAAGGAGGAGGGAACTCTGTCTTGGTGGAGGAAGAAGATGAGTCTTTCTCATCCT 1691
Brain       TCAGTCAAGGAGGAGGGAACTCTGTCTTGGTGGAGGAAGAAGATGAGTCTTTCTCATCCT 1692
            ** * *************** *********** ***************************

Testis      TCCCTCAATCTTCTGAGCCAGGTGCTTGGGGCTTTGCCTGTTTCTTTCTTCAGATGTGCG 1750
Ovary       TCCCTCAATCTTCTGAGCCAGGTGCTTGGGGCTTTGCCTGTTTCTTTCTTCAGATGTGCG 1750
Spleen      TCCCTCAATCTTCTGAGCCAGGTGCTTGGGGCTTTGCCTGTTTCTTTCTTCAGATGTGCG 1755
Liver       TCCCTCAATCTTCTGAGCCAGGTGCTTGGGGCTTTGCCTGTTTCTTTCTTCAGATGTGCG 1751
Lung        TCCCTCAATCTTCTGAGCCAGGTGCTTGGGGCTTTGCCTGTTTCTTTCTTCAGATGTGCG 1795
Heart       TCCCTCCATCTTCTGAGCCAGGTGCTTGGGGCTTTGCCTGTTTCTTTCTTCAGATGTGCG 1751
Brain       TCCCTCAATCTTCTGAGCCAGGTGCTTGGGGCTTTGCCTGTTTCTTTCTTCAGATGTGCG 1752
            ****** *****************************************************

Testis      TGTGTCTGTCTGTCTGT 1767
Ovary       TGTGTCTGTCTGTCTGT 1767
Spleen      TGTGTCTGTCTGTCTGT 1772
Liver       TGTGTCTGTCTGTCTGT 1768
Lung        TGTGTCTGTCTGTCTGT 1812
Heart       TGTGTCTGTCTGTCTGT 1768
Brain       TGTGTCTGTCTGTCTGT 1769
            *****************
```
