# Supplementary material for: Organization and differential expression of the GACA/GATA tagged somatic and spermatozoal transcriptomes in Buffalo Bubalus bubalis
Source: BMC Genomics. 2008 Mar 20;9:132. doi: 10.1186/1471-2164-9-132 (PMC2346481; doi:10.1186/1471-2164-9-132)
Supplement: Additional file 6 — Multiple nucleotide sequence alignment of GACA-tagged 1.3 kb transcript originating from different tissues and spermatozoa. The sequence from spermatozoa is highlighted in yellow background. The single nucleotide variations spread along the sequence shared by sperm and other tissues are highlighted in pink, and the ones common to tissues in blue. Several variations detected in sperm or testis only is shown in red. Note the exclusive insertion of 10 bp detected in sperm, highlighted in bold red and grey background. [file 1471-2164-9-132-S6.pdf]

Additional file 6: Multiple nucleotide sequence alignment of GACA-tagged 1.3 kb transcript originating from different tissues and spermatozoa of buffalo

```
SPERM           ACAGACAGACAGACAGCAGTGTTCCCTGTCCAGGCCGACCTGGAGGACCCACCTGGGGTG 60
Testis          ACAGACAGACAGACAGCAGTGTTCCCTGTCCAGGCCGACCTGGAGGACCCACCTGGGGTG 60
Ovary           ACAGACAGACAGACAGCAGTGTTCCCTGTCCAGGCCGACCTGGAGGACCCACCTGGGGTG 60
Spleen          ACAGACAGACAGACAGCAGTGTTCCCTGTCCAGGCCGACCTGGAGGACCCACCTGGGGTG 60
Liver           ACAGACAGACAGACAGCAGTGTTCCCTGTCCAGGCCAACCTGGAGGACCCACCTGGGGTG 60
Kidney          ACAGACAGACAGACAGCAGTGTTCCCTGTCCAGGCCAACCTGGAGGACCCACCTGGGGTG 60
                ************************************ ***********************

SPERM           AATTTCCTTCACTTAGTCTTTGCTGCCGCGGCTCGACTACCCGTCAGCCACTGTTGCTAG 120
Testis          AATTTCCTTCACTTAGTCTTTGCTGCCGCGGCTCGACTACCCATCAGCCACTGTTGCTAG 120
Ovary           AATTTCCTTCACTTAGTCTTTGCTGCCGCGGCTCGACTACCCGTCAGCCACTGTTGCTAG 120
Spleen          AATTTCCTTCACTTAGTCTTTGCTGCCGCGGCTCGACTACCCGTCAGCCACTGTTGCTAG 120
Liver           AATTTCCTTCACTTAGTCTTTGCTGCCGCGGCTCGACTACCCGTCAGCCACTGTTGCTAG 120
Kidney          AATTTCCTTCACTTAGTCTTTGCTGCCGCGGCTCGACTACCCGTCAGCCACTGTTGCTAG 120
                ****************************************** *****************

SPERM           TGAAAGTAAACACCCATACGGGCTACACCAAGGGCACCCTGTTGATTCAGAGCTCGTAAA 180
Testis          TGAAAGTAAACACCCATACGGGCTACACCAAGGGCACCCTGTTGATTCAGAGCTCGTAAA 180
Ovary           TGAAAGTAAACACCCATACGGGCTACACCAAGGGCACCCTGTTGATTCAGAGCTCGTAAA 180
Spleen          TGAAAGTAAACACCCATACGGGCTACACCAAGGGCACCCTGTTGATTCAGAGCTCGTAAA 180
Liver           TGAAAGTAAACACCCATACGGACTACACCAAGGGCACCCTGTTGATTCAGAGCTCGTAAA 180
Kidney          TGAAAGTAAACACCCATACGGGCTACACCAAGGGCACCCTGTTGATTCAGAGCTCGTAAA 180
                ********************* **************************************

SPERM           ACCCAGGAGCCCCAAGCCACTTTATGACGTGCCAGAATCCAGGGGCAGGAGCAAGGCCTG 240
Testis          ACCCAGGAGCCCCAAGCCACTTTATGACGTGCCAGAATCCAGGGGCAGGAGCAAAGCCTG 240
Ovary           ACCCAGGAGCCCCAAGCCACTTTATGACGTGCCGGAGTCCAGGGGCAGGAGCAAAGCCTG 240
Spleen          GCCCAGGAGCCCCAAGCCACTTTATGACGTGCCAGAATCCAGGGGCAGGAGCAAAGCCTG 240
Liver           ACCCAGGAGCCCCAAGCCACTTTATGACGTGCCAGAATCCAGGGGCAGGAGCAAAGCCTG 240
Kidney          ACCCAGGAGCCCCAAGCCACTTTATGACGTGCCAGAATCCAGGGGCAGGAGCAAAGCCTG 240
                 ******************************** ** ***************** *****

SPERM           GGGAAATCTCCCGGCTTCGAGGGCCACCGCGATGCTTCAGCAGGAGCAGAGGGGCTGGCC 300
Testis          GGGAAATCTCCCGGCTTCGAGGGCCACCGCGATGCTTCAGCAGGAGCAGAGGGGCTGGCC 300
Ovary           GGGAAATCTCCCGGCTTCGAGGGCCACCGCGATGCTTCAGCAGGAGCAGAGGGGCTGGCC 300
Spleen          GGGAAATCTCCCGGCTTCGAGGGCCACCGCGATGCTTCAGCAGGAGCAGAGGGGCTGGCC 300
Liver           GGGAAATCTCCCGGCTTCGAGGGCCACCGCGATGCTTCAGCAGGAGCAGAGGGGCTGGCC 300
Kidney          GGGAAATCTCCCGGCTTCGAGGGCCACCGCGATGCTTCAGCAGGAGCAGAGGGGCTGGCC 300
                ************************************************************

SPERM           TCCGCCCACAGGTGAGATGTGGGCCGTGGGCTTAGCTTTAAGCTGCTGACCCGCAGCGGC 360
Testis          TCCGCCCACAGGTGAGATGTGGGCCGTGGGCTTAGCTTTAAGCTGCTGACCCGCAGCGGC 360
Ovary           TCCGCCCACAGGTGAGATGTAGGCCGTGGGCTTAGCTTTAAGCTGCTGACCCGCAGCGGC 360
Spleen          TCCGCCCACAGGTGAGATGTAGGCCGTGGGCTTAGCTTTAAGCTGCTGACCCGCAGCGGC 360
Liver           TCCGCCCACAGGTGAGATGTGGGCCGTGGGCTTAGCTTTAAGCTGCTGACCCGCAGCGGC 360
Kidney          TCCGCCCACAGGTGAGATGTGGGCCGTGGGCTTAGCTTTAAGCTGCTGACCCGCAGCGGC 360
                ******************** ***************************************

SPERM           TTGCTTAGACCACTCTGGGGCTTGGGGGCTTGGGGGGCTGGCCCCGCTGATGGTTGAAGA 420
Testis          TTGCTTAGACCACTCTGGGGCTTGGGGGCTTGGGGGGCTGGCCCCGCTGATGGTTGAAGA 420
Ovary           TTGCTTAGACCACTCTGGGGCTTGGGGGCTTGGGGGGCTGGCCCCGCTGATGGTTGAAGA 420
Spleen          TTGCTTAGACCACTCTGGGGCTTGGGGGCTTGGGGGGCTGGCCCCGCTGATGGTTGAAGA 420
Liver           TTGCTTAGACCACTCTGGGGCTTGGGGGCTTGGGGGGCTGGCCCCGCTGATGGTTGAAGA 420
Kidney          TTGCTTAGACCACTCTGGGGCTTGGGGGCTTGGGGGGCTGGCCCCGCTGATGGTTGAAGA 420
                ************************************************************

SPERM           GCCAAACGGGACTTGGTGGGGGCCGTCGGGGGTGGTGGTGCAGGCCTGAGAGGCCCAGTG 480
Testis          GCCAAACGGGACTTGGTGGGGGCCGTCGGGGGTGGTGGTGCAGGCCTGAGAGGCCCAGTG 480
Ovary           GCCAAACGGGACTTGGTGGGGGCCGTCGGGGGTGGTGGTGCAGGCCTGAGAGGCCCAGTG 480
Spleen          GCCAAACGGGACTTGGTGGGGGCCGTCGGGGGTGGTGGTGCAGGCCTGAGAGGCCCAGTG 480
Liver           GCCAAACGGGACTTGGTGGGGGCCGTCGGGGGTGGTGGTGCAGGCCTGAGAGGCCCAGTG 480
Kidney          GCCAAACGGGACTTGGTGGGGGCCGTCGGGGGTGGTGGTGCAGGCCTGAGAGGCCCAGTG 480
                ************************************************************
```

```
SPERM           GGCTTGCGGGGAGCCCTGCCTCTGTGACGGTGTCACCCCAGTGAGCAGCCCTCAGAGAGG 540
Testis          GGCTTGCGGGGAGCCCTGCCTCTGTGACGGTGTCACCCCAGTGAGTAGCCCTCAGAGAGG 540
Ovary           GGCTTGCGGGGAGCCCTGCCTCTGTGACGGTGTCACCCCAGTGAGTAGCCCTCAGAGAGG 540
Spleen          GGCTTGCGGGGAGCCCTGCCTCTGTGACGGTGTCACCCCAGTGAGTAGCCCTCAGAGAGG 540
Liver           GGCTTGCGGGGAGCCCTGCCTCTGTGACGGTGTCACCCCAGTGAGTAGCCCTCAGAGAGG 540
Kidney          GGCTTGCGGGGAGCCCTGCCTCTGTGACGGTGTCACCCCAGTGAGTAGCCCTCAGAGAGG 540
                ********************************************* **************

SPERM           GAGAAGGAAGTGGACCCCGGGAGGGCACTTTTGGTTGCTGTTTGCCTCTCCTCAATTTCC 600
Testis          GAGAAGGAAGTGGACCCCGGGAGGGCACTTTTGGTTGCTGTTTGCCTCTCCTCAATTTCC 600
Ovary           GAGAAGGAAGTGGACCCCGGGAGGGCACTTTTGGTTGCTGTTTGCCTCTCCTCAATTTCC 600
Spleen          GAGAAGGAAGTGGACCCCGGGAGGGCACTTTTGGTTGCTGTTTGCCTCTCCTCAATTTCC 600
Liver           GAGAAGGAAGTGGACCCCGGGAGGGCACTTTTGGTTGCTGTTTGCCTCTCCTCAATTTCC 600
Kidney          GAGAAGGAAGTGGACCCCGGGAGGGCACTTTTGGTTGCTGTTTGCCTCTCCTCAATTTCC 600
                ************************************************************

SPERM           TCTTTCAGAGGCTAATTGGGATTCGTTTGCTTTGCTTCTGGGCTCTCTGAGGGTGCAGGG 660
Testis          TCTTTCAGAGGCTAATTGGGATTCGTTTGCTTTGCTTCTGGGCTCTCTGGGGGTGCAGGG 660
Ovary           TCTTTCAGAGGCTAATTGGGATTCGTTTGCTTTGCTTCTGGGCTCTCTGAGGGTGCAGGG 660
Spleen          TCTTTCAGAGGCTAATTGGGATTCGTTTGCTTTGCTTCTGGGCTCTCTGAGGGTGCAGGG 660
Liver           TCTTTCAGAGGCTAATTGGGATTCGTTTGCTTTGCTTCTGGGCTCTCTGAGGGTGCAGGG 660
Kidney          TCTTTCAGAGGCTAATTGGGATTCGTTTGCTTTGCTTCTGGGCTCTCTGAGGGTGCAGGG 660
                ************************************************* **********

SPERM           CCTCCCCTGGAGGAGGTCCCCTGGTCCTCAGGTGGCCTTTTAAGCGTGGGTAAGCGTGGG 720
Testis          CCTCCCCTGGAGGAGGTCCCCTGGTCCTCAGGTGGCCTTTTAAGTGT----------GGG 710
Ovary           CCTCCCCTGGAGGAGGTCCCCTGGTCCTCAGGTGGCCTTTTAAGTGT----------GGG 710
Spleen          CCTCCCCTGGAGGAGGTCCCCTGGTCCTCAGGTGGCCTTTTAAGTGT----------GGG 710
Liver           CCTCCCCTGGAGGAGGTCCCCTGGTCCTCAGGTGGCCTTTTAAGTGT----------GGG 710
Kidney          CCTCCCCTGGAGGAGGTCCCCTGGTCCTCAGGTGGCCTTTTAAGTGT----------GGG 710
                ******************************************** **          ***

SPERM           GCTTGGCTCTCAAGCAGAGCATGTGCAACAAGTCACCTGAAAAGATTGTAAGAATGCAAA 780
Testis          GCTTGGCTCTCAAGCAGAGCATGTGCAACAAGTCACCTGAAAAGATTGTAAGAATGCAAA 770
Ovary           GCTTGGCTCTCAAGCAGAGCATGTGCAACAAGTCACCTGAAAAGATTGTAAGAATGCAAA 770
Spleen          GCTTGGCTCTCAAGCAGAGCATGTGCAACAAGTCACCTGAAAAGATTGTAAGAATGCAAA 770
Liver           GCTTGGCTCTCAAGCAGAGCATGTGCAACAAGTCACCTGAAAAGATTGTAAGAATGCAAA 770
Kidney          GCTTGGCTCTCAAGCAGAGCATGTGCAACAAGTCACCTGAAAAGATTGTAAGAATGCAAA 770
                ************************************************************

SPERM           TGTCCAGCCTCCTCCAAGAGACGGCGGGGCCCAGGCTTAGGGGCGTGGCCTGGGTGGCTG 840
Testis          TGTCCAGCCTCCTCCAAGAGACGGCGGGGCCCAGGGTTAGGGGCGTGGCCTGGGTGGCTG 830
Ovary           TGTCCAGCCTCCTCCAAGAGACGGCGGGGCCCAGGGTTAGGGGCGTGGCCTGGGTGGCTG 830
Spleen          TGTCCAGCCTCCTCCAAGAGACGGCGGGGCCCAGGGTTAGGGGCGTGGCCTGGGTGGCTG 830
Liver           TGTCCAGCCTCCTCCAAGAGACGGCGGGGCCCAGGCTTAGGGGCGTGGCCTGGGTGGCTG 830
Kidney          TGTCCAGCCTCCTCCAAGAGACGGCGGGGCCCAGGGTTAGGGGCGTGGCCTGGGTGGCTG 830
                *********************************** ************************

SPERM           CATTTTTATTAAGCGCCTCCCCCAACCAGGTGACCTGGGAACCCCACTTTGAAAAATGCT 900
Testis          CATTTTTATTAAGCGCCTCCCCCAACCAGGTGACCTGGGAACCCCACTTTGAAAAATGCT 890
Ovary           CATTTTTATTAAGCGCCTCCCCCAACCAGGTGACCTGGGAACCCCACTTTGAAAAATGCT 890
Spleen          CATTTTTATTGAGCGCCTCCCCCAACCAGGTGACCTGGGAACCCCACTTTGAAAAATGCT 890
Liver           CATTTTTATTAAGCGCCTCCCCCAACCAGGTGACCTGGGAACCCCACTTTGAAAAATGCT 890
Kidney          CATTTTTATTAAGCGCCTCCCCCAACCAGGTGACCTGGGAACCCCACTTTGAAAAATGCT 890
                ********** *************************************************

SPERM           GGGGCTCAGGGCCAAGACACCTGGGTTTGGGGTCGGGATCCAGCAGGAACCGCGCCGATG 960
Testis          GGGGCTCAGGGCCAAGACACCTGGGTTTGGGGTCGGGATCCAGCAGGAACCGCGCCGATG 950
Ovary           GGGGCTCAGGGCCAAGACACCTGGGTTTGGGGTCGGGATCCAGCAGGAACCGCGCCGATG 950
Spleen          GGGGCTCAGGGCCAAGACACCTGGGTTTGGGGTCGGGATCCAGCAGGAACCGCGCCGATG 950
Liver           GGGGCTCAGGGCCAAGACACCTGGGTTTGGGGTCGGGATCCAGCAGGAACCACGCCGATG 950
Kidney          GGGGCTCAGGGCCAAGACACCTGGGTTTGGGGTCGGGATCCAGCAGGAACCGCGCCGATG 950
                *************************************************** ********

SPERM           AGCGCGGTACTGCGTGTAAACACGCGGACACAGTCGCAGGTATCGGTTGACACAGGGCAA 1020
Testis          AGCGCGGTACTGCGTGTAAACACGCGGGCACAGTCGCAGGTATCGGTTGACACAGGGCAA 1010
Ovary           AGCGCGGTACTGCGTGTAAACACGCGGGCACAGTCGCAGGTATCGGTTGACACAGGGCAA 1010
Spleen          AGCGCGGTACTGCGTGTAAACACGCGGGCACAGTCGCAGGTATCGGTTGACACAGGGCAA 1010
Liver           AGCGCGGTACTGCGTGTAAACACACGGGCACAGTCGCAGGTATCGGTTGACACAGGGCAA 1010
Kidney          AGCGCGGTACTGCGTGTAAACACGCGGACACAGTCGCAGGTATCGGTTGACACAGGGCAA 1010
                *********************** *** ********************************
```

```
SPERM           CCGCCCCAGGAACTGCAGCGCATCCTGTTGTTTGCTTTCCCAGCCGGGATTTATTAAATC 1080
Testis          CCGCCCCAGGAACTGCAGCGCATCCTGTTGTTTGCTTTCCCAGCCGGGATTTATTAAATC 1070
Ovary           CCGCCCCAGGAACTGCAGCGCATCCTGTTGTTTGCTTTCCCAGCCGGGATTTATTAAATC 1070
Spleen          CCGCCCCAGGAACTGCAGCGCATCCTGTTGTTTGCTTTCCCAGCCGGGATTTATTAAATC 1070
Liver           CCGCCCCAGGAACTGCAGCGCATCCTGTTGTTTGCTTTCCCAGCCGGGATTTATTAAATC 1070
Kidney          CCGCCCCAGGAACTGCAGCGCATCCTGTTGTTTGCTTTCCCAGCCGGGATTTATTAAATC 1070
                ************************************************************

SPERM           AAATATGCTTTCTGTGAATTCTCCCATTTAACCCTCAAACAGCCTGTGGCATGCGTCTGC 1140
Testis          AAATATGCTTTCTGTGAATTCTCCCATTTAACCCTCAAACAGCCTGTGGCATGCGTCTGC 1130
Ovary           AAATATGCTTTCTGTGAATTCTCCCATTTAACCCTCAAACAGCCTGTGGCATGCGTCTGC 1130
Spleen          AAATATGCTTTCTGTGAATTCTCCCATTTAACCCTCAAACAGCCTGTGGCATGCGTCTGC 1130
Liver           AAATATGCTTTCTGTGAATTCTCCCATTTAACCCTCAAACAGCCTGTGGCATGCGTCTGC 1130
Kidney          AAATATGCTTTCTGTGAATTCTCCCATTTAACCCTCAAACAGCCTGTGGCATGCGTCTGC 1130
                ************************************************************

SPERM           CGATGGCCCCGTTTTTCCGATTTGGCAAAGTGAGGTTTCGAGTCGGGAAGCCCCCTAAAG 1200
Testis          CGATGGCCCCGTTTTTCCGATTTGGCAAAGTGAGGTTTCGAGTCGGGAAGCCCCCTAAAG 1190
Ovary           CGATGGCCCCGTTTTTCCGATTTGGCAAAGTGAGGTTTCGAGTCGGGAAGCCCCCTAAAG 1190
Spleen          CGATGGCCCCGTTTTTCCGATTTGGCAAAGTGAGGTTTCGAGTCGGGAAGCCCCCTAAAG 1190
Liver           CGATGGCCCCGTTTTTCCGATTTGGCAAAGTGAGGTTTCGAGTCGGGAAGCCCCCTAAAG 1190
Kidney          CGATGGCCCCGTTTTTCCGATTTGGCAAAGTGAGGTTTCGAGTCGGGAAGCCCCCTAAAG 1190
                ************************************************************

SPERM           GGTCACCTGGTGGGGACTTGAGCTGAGACCGAGGACGGTCCCACGCGGCAGCCTCACCCG 1260
Testis          GGTCACCTGGTGGGGACTTGAGCTGAGACCGAGGACGGTCCCACGCGGCAGCCTCACCCG 1250
Ovary           GGTCACCTGGTGGGGACTTGAGCTGAGACCGAGGACGGTCCCACGCGGCAGCCTCACCCG 1250
Spleen          GGTCACCTGGTGGGGACTTGAGCTGAGACCGAGGACGGTCCCACGCGGCAGCCTCACCCG 1250
Liver           GGTCACCTGGTGGGGACTTGAGTTGAGACCGAGGACGGTCCCACGCGGCAGCCTCACCCG 1250
Kidney          GGTCACCTGGTGGGGACTTGAGCTGAGACCGAGGACGGTCCCACGCGGCAGCCTCACCCG 1250
                ********************** *************************************

SPERM           GGCAGGGGCTGCCCCTCCATGGAGCCGAGTTCTGAGCATGTCTGTCTGTCTGT 1313
Testis          GGCAGGGGCTGCCCCTCCATGGAGCCGAGTTCTGAGCATGTCTGTCTGTCTGT 1303
Ovary           GGCAGGGGCTGCCCCTCCATGGAGCCGAGTTCTGAGCATGTCTGTCTGTCTGT 1303
Spleen          GGCAGGGGCTGCCCCTCCATGGAGCCGAGTTCTGAGCATGTCTGTCTGTCTGT 1303
Liver           GGCAGGGGCTGCCCCTCCATGGAGCCGAGTTCTGAGCATGTCTGTCTGTCTGT 1303
Kidney          GGCAGGGGCTGCCCCTCCATGGAGCCGAGTTCTGAGCATGTCTGTCTGTCTGT 1303
                *****************************************************
```
