# Supplementary material for: Organization and differential expression of the GACA/GATA tagged somatic and spermatozoal transcriptomes in Buffalo Bubalus bubalis
Source: BMC Genomics. 2008 Mar 20;9:132. doi: 10.1186/1471-2164-9-132 (PMC2346481; doi:10.1186/1471-2164-9-132)
Supplement: Additional file 7 — Multiple sequence alignment of GACA-tagged 850 bp transcript originating from different tissues and spermatozoa, homologous to HBGF-1. The sequence from the spermatozoa is highlighted in yellow background. The single nucleotide variations along the sequence but common across the tissues are highlighted in same color (pink or blue). Several variations detected in sperm or testis only are shown in red and the ones exclusive to ovary in blue background. [file 1471-2164-9-132-S7.pdf]

Additional file 7: Multiple alignment of GACA-tagged 850 bp transcript (Homologous to HBGF-1) originated from different tissues and spermatozoa of buffalo

```
SPERM            ACAGACAGACAGACAGACAGACA--CACACACACACACACTGTCTCTCTCTCTCTCTCTC 58
Testis           --------ACAGACAGACAGACAG--ACACACACACACTGTCTCTCTCTCTCTCTCTCTC 50
Ovary            --------ACAGACAGACAGACA--CACACACACGCACTGTCTCTCTCTCTCTCTCTCTC 50
Spleen           --------ACAGACAGACAGACAGACACACACACACACTGTCTCTCTCTCTCTCTCTCTC 52
Liver            --------ACAGACAGACAGACA--CACACACACACACTGTCTCTCTCTCTCTCTCTCTC 50
Lung             ACAGACAGACAGACAGACAGACAGACACACACACACACACTGTCTCTCTCTCTCTCTCTC 60
Kidney           --------ACAGACAGACAGACA--CACACACACACACTGTCTCTCTCTCTCTCTCTCTC 50
Heart            --------ACAGACAGACAGACA--CACACACACACACTGTCTCTCTCTCTCTCTCTCTC 50
                         ***************   ******** ***  * ******************

SPERM           TCTCTCTCCCTCTCTGCCTCAGCACTGGATGTATATAACCTGCTTTCTGCTCCCGAGAGT 118
Testis          TCTCTCTCTCTCTCTGCCTCAGCACTGGATGTATATAACCTGCTTTCTGCTCCTGAGAGT 110
Ovary           TCTCTCTCTCTCTCTGCCTCAGCACTGGATGTATATAACCTGCTTTCTGCTCCCGAGAGT 110
Spleen          TCTCTCTCTCTCTCTGCCTCAGCACTGGATGTATATAACCTGCTTTCTGCTCCCGAGAGT 112
Liver           TCTCTCTCTCTCTCTGCCTCAGCACTGGATGTATATAACCTGCTTTCTGCTCCTGAGAGT 110
Lung            TCTCTCTCTCTCTCTGCCTCAGCACTGGATGTATATAACCTGCTTTCTGCTCCCGAGAGT 120
Kidney          CCTCTCTCTCTCTCTGCCTCAGCACTGGATGTATATAACCTGCTTTCTGCTCCTGAGAGT 110
Heart           TCTCTCTCTCTCTCTGCCTCAGCACTGGATGTATATAACCTGTTTTCTGCTCCTGAGAGT 110
                 ******* ********************************* ********** ******

SPERM           AGAAACCCTGATTCCTACCTGAACAGCTTGCAGGCTGAGGGCTTCTCCTCTCCAGTCGAA 178
Testis          AGAAACCCTGATTCCTACCTGAACAGCTTGCAGGCTGAGGGCTCCTCCTCTCCAGTCGAA 170
Ovary           AGAAACCCTGATTCCTACCTGAACAGCTTGCAGGCTGAGGGCTTCTCCTCTCCAGTCGAA 170
Spleen          AGAAACCCTGATTCCTACCTGAACAGCTTGCAGGCTGAGGGCTTCTCCTCTCCAGTCGAA 172
Liver           AGAAACCCTGATTCCTACCTGAACAGCTTGCAGGCTGAGGGCTTCTCCTCTCCAGTCGAA 170
Lung            AGAAACCCTGATTCCTACCTGAACAGCTTGCAGGCTGAGGGCTTCTCCTCTCCAGTCGAA 180
Kidney          AGAAACCCTGATTCCTACCTGAACAGCTTGCAGGCTGAGGGCTTCTCCTCTCCAGTCGAA 170
Heart           AGAAACCCTGATTCCTACCTGAACAGCTTGCAGGCTGAGGGCTTCTCCTCTCCAGTCGAA 170
                ******************************************* ****************

SPERM           GGGAAAGAGGATCCTCTCAGTCTCAGCCGCCCTGCGTTGTCACTGTCAGTGGGCTGCGAC 238
Testis          GGGAAAGAGGATCCTCTCAGTCTCAGCCGCCCTGCGTTGTCACTGTCAGTGGGCTGCGAC 230
Ovary           GGGAAAGAGGATCCTCTCAGTCTCAGCTGCCCTGCGTTGTCACTGTCAGTGGGCTGCGAC 230
Spleen          GGGAAAGAGGATCCTCTCAGTCTCAGCCGCCCTGCGTTGTCACTGTCAGTGGGCTGCGAC 232
Liver           GGGAAAGAGGATCCTCTCAGTCTCAGCCGCCCTGCGTTGTCACTGTCAGTGGGCTGCGAC 230
Lung            GGGAAAGAGGATCCTCTCAGTCTCAGCCGCCCTGCGTTGTCACTGTCAGTGGGCTGCGAC 240
Kidney          GGGAAAGAGGATCCTCTCAGTCTCAGCCGCCCTGCGTTGTCACTGTCAGTGGGCTGCGAC 230
Heart           GGGAAAGAGGATCCTCTCAGTCTCAGCCGCCCTGCGTTGTCACTGTCAGTGGGCTGCGAC 230
                *************************** ********************************

SPERM           TGCTTCTCCAGCAGCCCCAAGCTGGCTCTCTGCACCTCCCGGTGACTGCGGCTCTGTGTG 298
Testis          TGCTTCTCCAGCAGCCCCAAGCTGGCTCTCTGCACCTCCCGGTGACTGCGGCTCTGTGTG 290
Ovary           TGCTTCTCCAGCAGCCCCAAGCTGGCTCTCTGCACCTCCCGGTGACTGCGGCTCTGTGTG 290
Spleen          TGCTTCTCCAGCAGCCCCAAGCTGGCTCTCTGCACCTCCCGGTGACTGCGGCTCTGTGTG 292
Liver           TGCTTCTCCAGCAGCCCCAAGCTGGCTCTCTGCACCTCCCGGTGACTGCGGCTCTGTGTG 290
Lung            TGCTTCTCCAGCAGCCCCAAGCTGGCCCTCTGCACCTCCCGGTGACTGCGGCTCTGTGTG 300
Kidney          TGCTTCTCCAGCAGCCCCAAGCTGGCTCTCTGCACCTCCCGGTGACTGCGGCTCTGTGTG 290
Heart           TGCTTCTCCAGCAGCCCCAAGCTGGCTCTCTGCACCTCCCGGTGACTGCGGCTCTGTGTG 290
                ************************** *********************************

SPERM           GGGAGAGGGGACGGGCCGTGGCATGTACATCAGGTTGCTGGTCCGCCTCCAAACACCGTA 358
Lung            GGGAGAGGGGACGGGCCGTGGCATGTACATCAGGTTGCTGGTCCGCCTCCAAACACCGTA 360
Spleen          GGGAGAGGGGACGGGCCGTGGCATGTACATCAGGTTGCTGGTCCGCCTCCAAACACCGTA 352
Ovary           GGGAGAGGGGACGGGCCGTGGCATGTACATCAGGTTGCTGGTCCGCCTCCAAACACCGTA 350
Testis          GGGAGAGGGGACGGGCCGTGGCATGTACATCAGGTTGCTGGTCCGCCTCCAAACACCGTA 350
Kidney          GGGAGAGGGGACGGGCCGTGGCATGTACATCAGGTTGCTGGTCCGCCTCCAAACACCGTA 350
Liver           GGGAGAGGGGACGGGCCGTGGCATGTACATCAGGTCGCTGGTCCGCCTCCAAACACCGTA 350
```

```
Heart          GGGAGAGGGGACGGGCCGTGGCATGTACGTCAGGTTGCTGGTCCGCCTCCAAACACCGTA 350
               **************************** ****** ************************

SPERM          CTCCAGAGCCGGCTCCCAGTCCTGCAGGGCTGTGTGGGAAGCAGGAAGGCAGGCAGGGGG 418
Testis         CTCCAGAGCCGGCTCCCAGTCCTGCAGGGCTGTGTGGGAAGCAGGAAGGCAGGCAGGGGG 410
Ovary          CTCCAGAGCCGGCTCCCAGTCCTGCAGGGCTGTGTGGGAAGCAGGAAGGCAGGCAGGGGG 410
Spleen         CTCCAGAGCCGGCTCCCAGTCCTGCAGGGCTGTGTGGGAAGCAGGAAGGCAGGCAGGGGG 412
Liver          CTCCAGAGCCGGCTCCCAGTCCTGCAGGGCTGTGTGGGAAGCAGGAAGGCAGGCAGGGGG 410
Lung           CTCCAGAGCCGGCTCCCAGTCCTGCAGGGCTGTGTGGGAAGCAGGAAGGCAGGCAGGGGG 420
Kidney         CTCCAGAGCCGGCTCCCAGTCCTGCAGGGCTGTGTGGGAAGCAGGAAGGCAGGCAGGGGG 410
HEART          CTCCAGAGCCGGCTCCCAGTCCTGCAGGACTGTGTGGGAAGCAGGAAGACAGGCAGGGGG 410
               **************************** ******************* ***********

SPERM          CTGAGCATGCAGCCTGTAGGTCTCTCTCCAGCCCCGTTTTGATCGGCAGAGGGGACCTGC 478
Testis         CTGGGCATGCAGCCTGTAGGTCTCTCTCCAGCCCCGTTTTGATCGGCAGAGGGGACCTGC 470
Ovary          CTGAGCATGCAGCCTGTAGGTCTCTCTCCAGCCCCGTTTTGATCGGCAGAGGGGACCTGC 470
Spleen         CTGAGCATGCAGCCTGTAGGTCTCTCTCCAGCCCCGTTTTGATCGGCAGAGGGGACCTGC 472
Liver          CTGAGCATGCAGCCTGTAGGTCTCTCTCCAGCCCCGTTTTGATCGGCAGAGGGGACCTGC 470
Lung           CTGAGCATGCAGCCTGTAGGCCTCTCTCCAGCCCCGTTTTGATCGGCAGAGGGGACCTGC 480
Kidney         CTGAGCATGCAGCCTGTAGGTCTCTCTCCAGCCCCGTTTTGATCGGCAGAGGGGACCTGC 470
Heart          CTGAGCATGCAGCCTGTAGGTCTCTCTCCAGCCCCGTTTTGACCGGCAGAGGGGACCTGC 470
               *** **************** ********************* *****************

SPERM          CTTTAGACAGCTGCCTGCACTGACCCTGCCCACAGGGGTGCACACGGAGTACCCCTAGCC 538
Testis         CTTTAGACAGCTGCCTGCACCGACCCTGCCCACAGGGGTGCACACGGAGTACCCCTAGCC 530
Ovary          CTTTAAACAGCTGCCTGCACCGACCCTGCCCACAGGGGTGCACACGGAGTACCCCTAGCC 530
Spleen         CTTTAGACAGCTGCCTGCACTGACCCTGCCCACAGGGGTGCACACGGAGTACCCCTAGCC 532
Liver          CTTTAGACAGCTGCCTGCACCGACCCTGCCCACAGGGGTGCACACGGAGTACCCCTAGCC 530
Lung           CTTTAGACAGCTGCCTGCACTGACCCTGCCCACAGGGGTGCACACGGAGTACCCCTAGCC 540
Kidney         CTTTAGACAGCTGCCTGCACCGACCCTGCCCACAGGGGTGCACACGGAGTACCCCTAGCC 530
Heart          CTTTAGACAGCTGCCTGCACCGACCCTGCCCACAGGGGTGCACACGGAGTACCCCTAGCC 530
               ***** ************** ***************************************

SPERM          TTGGGCAACCAACAGCAGGCCCAGGGACACAGAGCTCATGGCCAGCGGGGAAGGGAGAAA 598
Testis         TTGGGCAACCAACAGCAGGCCCAGGGACACAGAGCTCATGGCCAGCGGGGAAGGGAGAAA 590
Ovary          TTGGGCAACCAACAGCAGGCCCAGGGACACAGAGCTCATGGCCAGCGGGGAAGGGAGAAA 590
Spleen         TTGGGCAACCAACAGCAGGCCCAGGGACACAGAGCTCATGGCCAGCGGGGAAGGGAGAAA 592
Liver          TTGGGCAACCAACAGCAGGCCCAGGGACACAGAGCTCATGGCCAGCGGGGAAGGGAGAAA 590
Lung           TTGGGCAACCAACAGCAGGCCCAGGGACACAGAGCTCATGGCCAGCGGGGAAGGGAGAAA 600
Kidney         TTGGGCAACCAACAGCAGGCCCAGGGACACAGAGCTCATGGCCAGCGGGGAAGGGAGAAA 590
Heart          TTGGGCAACCAACGGCAGGCCCAGGGACACAGAGCTCACGGCCAGCGGGGAAGGGAGAAA 590
               ************* ************************ *********************

SPERM          GAGACAGCAGACAGCGGCGGCAGCTCTCCTGGGTGTTATTTTAACGTGGTTTGTCTTGGG 658
Testis         GAGACAGCAGACAGCGGCGGCAGCTCTCCTGGGTGTTATTTTAACGTGGTTTGTCTTGGG 650
Ovary          GAGACGGCAGACAGCGGCGGCAGCTCTCCTGGGTGTTATTTTAACGTGGTTTGTCTTGGG 650
Spleen         GAGACAGCAGACAGCGGCGGCAGCTCTCCTGGGTGTTATTTTAACGTGGTTTGTCTTGGG 652
Liver          GAGACAGCAGACAGCGGCGGCAGCTCTCCTGGGTGTTATTTTAACGTGGTTTGTCTTGGG 650
Lung           GAGACAGCAGACAGCGGCGGCAGCTCTCCTGGGTGTTATTTTAACGTGGTTTGTCTTGGG 660
Kidney         GAGACAGCAGACAGCGGCGGCAGCTCTCCTGGGTGTTATTTTAACGTGGTTTGTCTTGGG 650
Heart          GAGACAGCAGACAGCGGCGGCAGCTCTCCTGGGTGTTATTTTAACGTGGTTTGTCTTGGG 650
               ***** ******************************************************

SPERM          GCAAATGGCTGCACTGAATGGACTACTAGCCCGTCTGGGCAGATTCCTGTTTATTTTGAG 718
Testis         GCAAATGGCTGCACTGAATGGACTACTAGCCCGTCTGGGCAGATTCCTGTTTATTTTGAG 710
Ovary          GCAAATGGCTGCACTGAATGGACTACTAGCCCGTCTGGGCAGATTCCTGTTTATTTTGAG 710
Spleen         GCAAATGGCTGCACTGAATGGACTACTAGCCCGTCTGGGCAGATTCCTGTTTATTTTGAG 712
Liver          GCAAATGGCTGCACTGAATGGACTACTAGCCCGTCTGGGCAGATTCCTGCTTATTTTGAG 710
Lung           GCAAATGGCTGCACTGAATGGACTACTAGCCCGTCTGGGCAGATTCCTGTTTATTTTGAG 720
Kidney         GCAAATGGCTGCACTGAATGGACTACTAGCCCGTCTGGGCAGATTCCTGTTTATTTTGAG 710
Heart          GCAAATGGCTGCACTGAATGGACTACTAGCCCGTCTGGGCAGATTCCTGTTTATTTTGAG 710
               ************************************************* **********
SPERM          GCCTCAGTTGGCAGGGAAACAGCAGGTCATGGCTCAGAGACACTCCTCTGCAATCAGAAG 778
```

```
Testis          GCCTCAGTTGGCAGGGAAACAGCAGGTCATGGCTCAGAGACACTCCTCTGCAATCAGAAG 770
Ovary           GCCTCAGTTGGCAGGGAAACAGCAGGTCATGGCTCAGAGACACTCCTCTGCAATCAGAAG 770
Spleen          GCCTCAGTTGGCAGGGAAACAGCAGGTCATGGCTCAGAGACACTCCTCTGCAATCAGAAG 772
Liver           GCCTCAGTTGGCAGGGAAACAGCAGGTCATGGCTCAGAGACACTCCTCTGCAATCAGAAG 770
Lung            GCCTCAGTTGGCAGGGAAACAGCAGGTCATGGCTCAGAGACACTCCTCTGCAATCAGAAG 780
Kidney          GCCTCAGTTGGCAGGGAAACAGCAGGTCATGGCTCAGAGACACTCCTCTGCAATCAGAAG 770
Heart           GCCTCAGTTGGCAGGGAAACAGCAGGTCATGGCTCAGAGACACTCCTCTGCAATCAGAAG 770
                ************************************************************

SPERM           ACTGCTGGATGAAAAGGAATCCTTTTCTGTTCTGACTTCTGGCATCTCTGCACAGGGTGT 838
Testis          ACTGCTGGATGAAAAGGAATCCTTTTCTGTTCTGACTTCTGGCATCTCTGCACAGGGTGT 830
Ovary           ACTGCTGGATGAAAAGGAATCCTTTTCTGTTCTGACTTCTGGCATCTCTGCACAGGGTGT 830
Spleen          ACTGCTGGATGAAAAGGAATCCTTTTCTGTTCTGACTTCTGGCATCTCTGCACAGGGTGT 832
Liver           ACTGCTGGATGAAAAGGAATCCTTTTCTGTTCTGACTTCTGGCATCTCTGCACAGGGTGT 830
Lung            ACTGCTGGATGAAAAGGAATCCTTTTCTGTTCTGACTTCTGGCATCTCTGCACAGGGTGT 840
Kidney          ACTGCTGGATGAAAAGGAATCCTTTTCTGTTCTGACTTCTGGCATCTCTGCACAGGGTGT 830
Heart           ACTACTGGATGAAAAGGAATCCTTTTCTGTTCTGACTTCTGGCATCTCTGCACAGGGTGT 830
                *** ********************************************************

SPERM           AGATGTCTGTCTGTCTGT 857
Testis          AGATGTCTGTCTGTCTGT 848
Ovary           AGATGTCTGTCTGTCTGT 848
Spleen          AGATGTCTGTCTGTCTGT 850
Liver           AGATGTCTGTCTGTCTGT 848
Lung            AGATGTCTGTCTGTCTGT 858
Kidney          AGATGTCTGTCTGTCTGT 848
Heart           AGATGTCTGTCTGTCTGT 848
                ******************
```
