# Supplementary material for: Organization and differential expression of the GACA/GATA tagged somatic and spermatozoal transcriptomes in Buffalo Bubalus bubalis
Source: BMC Genomics. 2008 Mar 20;9:132. doi: 10.1186/1471-2164-9-132 (PMC2346481; doi:10.1186/1471-2164-9-132)
Supplement: Additional file 8 — Multiple sequence alignment of GACA-tagged 635 bp transcript originating from spermatozoa and different tissues, representing WASF2 gene. The sequence from spermatozoa is highlighted in yellow background. Several variations detected in sperm or testis only are shown in red, and that in somatic tissues are in blue color. Note the single nucleotide variations/insertions/deletions along the sequences from different tissues with highest frequency in testis. [file 1471-2164-9-132-S8.pdf]

## Additional file 8: Multiple alignment of GACA-tagged 635 bp transcript originating from spermatozoa and different tissues of buffalo

```
SPERM           ACAGACAGACAGACACATCACTTGGGA-CAAAAAAACCAAAGCAAAAGCCAGAGGTCATT 59
Testis          ACAGACAGACAGACACATCACTTGGGGGCAAAAAAACCAAAGCAAAAGCCCAGAGTCATT 60
Ovary           ACAGACAGACAGACACATCACTTGGGA-CAAAAAAACCAAAGCAAAAGCCAGAGGTCATT 59
Spleen          ACAGACAGACAGACACATCACTTGGGA-CAAAAAAACCAAAGCAAAAGCCAGAGGTCATT 59
Liver           ACAGACAGACAGACACATCACTTGGGA-CAAAAAAACCAAAGCAAAAGCCAGAGGTCATT 59
Kidney          ACAGACAGACAGACACATCACTTGGGA-CAAAAAAACCAAAGCAAAAGCCAGAGGTCATT 59
Heart           ACAGACAGACAGACACATCACTTGGGA-CAAAAAAACCAAAGCAAAAGCCAGAGGTCATT 59
                **************************  **********************    ******

SPERM          A-TCGT-ATCCTGAAGAA-CTAGGAGGGAGAAACAGCTCAGTGGG-TCACGGGAAGGCTA 115
Testis         AATCGTTATCCTGAAAAAACTAGGAGGGAGAA-CAGCTCAGTGGGGTCACGGGAAGGCTA 119
Ovary          A-TCGT-ATCCTGAAGAA-CTAGGAGGGAGAAACAGCTCAGTGGG-TCACGGGAAGGCTA 115
Spleen         A-TCGT-ATCCTGAAGAA-CTAGGAGGGAGAAACAGCTCAGTGGG-TCACGGGAAGGCTA 115
Liver          A-TCGT-ATCCTGAAGAA-CTAGGAGGGAGAAACAGCTCAGTGGG-TCACGGGAAGGCTA 115
Kidney         A-TCGT-ATCCTGAAGAA-CTAGGAGGGAGAAACAGCTCAGTGGG-TCACGGGAAGGCTA 115
Heart          A-TCGT-ATCCTGAAGAA-CTAGGAGGGAGAAACAGCTCAGTGGG-TCACGGGAAGGCTA 115
               * **** ******** ** ************* ************ **************

SPERM          GGATAGGATGGACTGGCCCCTGTGCGCCCCTGCGCACAGCAGACATACTAGGCAGAGCGG 175
Testis         GGATAGGATG-ACTGGCCC-TGTGC-CCCCTGCGCACAGCAGACATACTAAGCAGAGCGC 176
Ovary          GGATAGGATGGACTGGCCCCTGTGCGCCCCTGCGCACAGCAGACATACTAGGCAGAGCGG 175
Spleen         GGATAGGATGGACTGGCCCCTGTGCGCCCCTGCGCACAGCAGACATACTAGGCAGAGCGG 175
Liver          GGATAGGATGGACTGGCCCCTGTGCGCCCCTGCGCACAGCAGACATACTAGGCAGAGCGG 175
Kidney         GGATAGGATGGACTGGCCCCTGTGCGCCCCTGCGCACAGCAGACATACTAGGCAGAGCGG 175
Heart          GGATAGGATGGACTGGCCCCTGTGCGCCCCTGCGCACAGCAGACATACTAGGCAGAGCGG 175
               ********** ******** ***** ************************ ********

SPERM          CAGCCCTGGGTTTCAGTTCTGACTCTGCTGTGTTACTCTGG-AGCAGTTGCTGCCCCTCC 234
Testis         AGCCCCTGGGTTTCAGTTCTGACTCTGCTGTGTTACCCTGGGAGCAGTTGCTGCCCCTCC 236
Ovary          CAGCCCTGGGTTTCAGTTCTGACTCTGCTGTGTTACCCTGG-AGCAGTTGCTGCCCCTCC 234
Spleen         CAGCCCTGGGTTTCAGTTCTGACTCTGCTGTGTTACCCTGG-AGCAGTTGCTGCCCCTCC 234
Liver          CAGCCCTGGGTTTCAGTTCTGACTCTGCTGTGTTACCCTGG-AGCAGTTGCTGCCCCTCC 234
Kidney         CAGCCCTGGGTTTCAGTTCTGACTCTGCTGTGTTACTCTGG-AGCAGTTGCTGCCCCTCC 234
Heart          CAGCCCTGGGTTTCAGTTCTGACTCTGCTGTGTTACTCTGG-AGCAGTTGCTGCCCCTCC 234
                  ********************************* **** ******************

SPERM          CTGGGTCACTCTTCTGCTTCTAACAAAG-AGAGAGCTGCAGTTGGTGATTTCTGAGGCAA 293
Testis         TGGGGTCACCCTCCTGCTTCCTACAAAGGAGAGAGCTGCAGTGGGTGATTTCTGAGGCAA 296
Ovary          CTGGGTCACTCTTCTGCTTCTAACAAAG-AGAGAGCTGCAGTTGGTGATTTCTGAGGCAA 293
Spleen         CTGGGCCACTCTTCTGCTTCTAACAAAG-AGAGAGCTGCAGTTGGTGATTTCTGAGGCAA 293
Liver          CTGGGTCACTCTTCTGCTTCTAACAAAG-AGAGAGCTGCAGTTGGTGATTTCTGAGGCAA 293
Kidney         CTGGGTCACTCTTCTGCTTCTAACAAAG-AGAGAGCTGCAGTTGGTGATTTCTGAGGCAA 293
Heart          CTGGGTCACTCTTCTGCTTCTAACAAAG-AGAGAGCTGCAGTTGGTGATTTCTGAGGCAA 293
                 *** *** ** *******  ****** ************* *****************

SPERM          CC-AAAATTCTCAAAGACTCTGCAGCAGTTGTAT-AACATAACAGGGAGGGGGCCCGGGA 351
Testis         CCCAAAATTCTCAAAGACTCTGCAGCAGTTGTAT-TACATAACAGGGAGGGGGCCCGGGA 355
Ovary          CC-AAAATTCTCAAAGACTCTGCAGCAGTTGTAT-AACATAACAGGGAGGGGGCCCGGGA 351
Spleen         CC-AAAATTCTCAAAGACTCTGCAGCAGTTGAAT-AACATAACAGGGAGGGGGCCCGGGA 351
Liver          CC-AAAATTCTCAAAGACTCTGCAGCAGTTGTAT-AACATAACAGGGAGGGGGCCCGGGA 351
Kidney         CC-AAAATTCTCAAAGACTCTGCAGCAGTTGTATTAACATAACAGGGAGGGGGCCCGGGA 352
Heart          CC-AAAATTCTCAAAGACTCTGCAGCAGTTGTAT-AACATAACAGGGAGGGGGCCCGGGA 351
               ** **************************** **   ************************

SPERM          GCCTTGGGAGTCCGATTAGAGTAAT-AAGAGGCCCCTCCTCAGGGGCTGCCCCACCCCTG 410
Testis         GCTTGGGGAGTCGGATTAGAGTCATTAGGAGGCCCCTCCTCAGGGGCGGCCCCACCCCTG 415
Ovary          GCCTTGGGAGTCCGATTAGAGTAAT-AAGAGGCCCCTCCTCAGGGGCTGCCCCACCCCTG 410
Spleen         GCCTTGGGAGTCCGATTAGAGTAAT-AAGAGGCCCCTCCTCAGGGGCTGCCCCACCCCTG 410
Liver          GCCTTGGGAGTCCGATTAGAGTAAT-AAGAGGCCCCTCCTCAGGGGCTGCCCCACCCCTG 410
```

```
Kidney         GCCTTGGGAGTCCGATTAGAGTAAT-AAGAGGCCCCTCCTCAGGGGCTGCCCCACCCCTG 411
Heart          GCCTTGGGAGTCCGATTAGAGTAAT-AAGAGGCCCCTCCTCAGGGGCTGCCCCACCCCTG 410
               ** * ******* ********* ** * ******************* ************

SPERM          CAGCCTCCTCCCCCTTCTAGGCTGGGCTCTCCCCTGGGTTGAAGCACCTGAAGCCAACAG 470
Testis         CAGCTTCCTCCCCCTTCTAGGCTGGGCCTCCCCCTGGGTTGAAGCACCTGGAGCCAACAG 475
Ovary          CAGCCTCCTCCCCCTTCTAGGCTGGGCTCTCCCCTGGGTTGAAGCACCTGAAGCCAACAG 470
Spleen         CAGCCTCCTCCCTCTTCTAGGCTGGGCTCTCCCCTGGGTTGAAGCACCTGAAGCCAACAG 470
Liver          CAGCCTCCTCCCCCTTCTAGGCTGGGCTCTCCCCTGGGTTGAAGCACCTGAAGCCAACAG 470
Kidney         CAGCCTCCTCCCCCTTCTAGGCTGGGCTCTCCCCTGGGTTGAAGCACCTGAAGCCAACAG 471
Heart          CAGCCTCCTCCCCCTTCTAGGCTGGGCTCTCCCCTGGGTTGAAGCACCTGAAGCCAACAG 470
               **** ******* **************   ******************** *********

SPERM          CACCCCCTGCCCCCTAGTACCTATAAACACATGTGCTCCTCTAAGCCTGAGCCATGTG-- 528
Testis         CACCCCCTGCCCCCTAGCACCTATAAACACATGTGCTCCTCTAAGC-TGAGCCATGTGGT 534
Ovary          CACCCCCTGCCCCCTAGTACCTATAAACACATGTGCTCCTCTAAGCCTGAGCCATGTG-- 528
Spleen         CACCCCCTGCCCCCTAGTACCTATAAACACATGTGCTCCTCTAAGCCTGAGCCATGTG-- 528
Liver          CACCCCCTGCCCCCTAGTACCTATAAACACATGTGCTCCTCTAAGCCTGAGCCATGTG-- 528
Kidney         CACCCCCTGCCCCCTAGTACCTATAAACACATGTGCTCCTCTAAACCTGAGCCATGTG-- 529
Heart          CACCCCCTGCCCCCTAGTACCTATAAACACATGTGCTCCTCTAAGCCTGAGCCATGTG-- 528
               ***************** ************************** * ***********

SPERM          TTTCGCCCT--CCCTGGACCTTAGGCCTTCC-AGGTTTGTGTATTTAGGGGCTCACATTC 585
Testis         CTCCACCCCTCCTCTGGACCTTAGGCTTTCCCAGGTTTGTGAATTTAGGGGCTCACATTC 594
Ovary          TTTCACCCT--CTCTGGACCTTAGGCTTTCC-AGGTTTGTGTCTTTAGGGGCTCACATTC 585
Spleen         TTTCACCCT--CTCTGGACCTTAGGCTTTCC-AGGTTTGTGTATTTAGGGGCTCACATTC 585
Liver          TTTCACCCT--CTCTGGACCTTAGGCTTTCC-AGGTTTGTGTATTTAGGGGCTCACATTC 585
Kidney         TTTCGCCCT--CTCTGGAC-TTAGGCTTTCC-AGGTTTGTGTATTTAGGGGCTCACATTC 585
Heart          TTTCGCCCT--CTCTGGACCTTAGGCTTTCC-AGGTTTGTGTATTTAGGGGCTCACATTC 585
                * * ***   * ****** ****** **** *********  *****************

SPERM          -GGGCTC-TGTGCCGGTGAGCCAGTC-TATGTGTGCACTGTCTGTCTGTCTGT 635
Testis         CGGGCTCCTGTGCCGGTGAGCCAGTCCTATGTGTGCACTGTCTGTCTGTCTGT 647
Ovary          -GGGCTC-TGTGCCGGTGAGCCAGTC-TATGTGTGCACTGTCTGTCTGTCTGT 635
Spleen         -GGGCTC-TGTGCCGGTGAGCCAGTC-TATGTGTGCACTGTCTGTCTGTCTGT 635
Liver          -GGGCTC-TGTGCCGGTGAGCCAGTC-TATGTGTGCACTGTCTGTCTGTCTGT 635
Kidney         -GGGCTC-TGTGCCGGTGAGCCAGTC-TATGTGTGCACTGTCTGTCTGTCTGT 635
Heart          -GGGCTC-TGTGCCGGTGAGCCAGTC-TATGTGTGCACTGTCTGTCTGTCTGT 635
                ****** ****************** **************************
```
