# Supplementary material for: Organization and differential expression of the GACA/GATA tagged somatic and spermatozoal transcriptomes in Buffalo Bubalus bubalis
Source: BMC Genomics. 2008 Mar 20;9:132. doi: 10.1186/1471-2164-9-132 (PMC2346481; doi:10.1186/1471-2164-9-132)
Supplement: Additional file 9 — Multiple sequence alignment of GACA-tagged 523 bp transcript originating from testis, ovary and spermatozoa only, homologous to Ankyrin repeat domain-26. The sequence from spermatozoa is highlighted in yellow background. Note identical sequences in testis and spermatozoa (highlighted in red) in comparison to ovary (blue). [file 1471-2164-9-132-S9.pdf]

Additional file 9: Multiple sequence alignment of GACA-tagged 523 bp transcript (Homologous to ankyrin repeat domain 26)

```
SPERM                ACAGACAGACAGACAGCAAGAGGACCACACAGACTTCCAAAGCCCCGGCCGCCACACCCT 60
Testis               ACAGACAGACAGACAGCAAGAGGACCACACAGACTTCCAAAGCCCCGGCCGCCACACCCT 60
Ovary                ACAGACAGACAGACAGCAAGAGGACCACACAGACTTCCAAAGCCCCGGCCGCCACACCCT 60
                     ************************************************************

SPERM                GCCGGCTCCATTCTGGGTTCCCCCCGGCCCCCAGTCGACCAGACCCCCACCGTCGCCCAC 120
Testis               GCCGGCTCCATTCTGGGTTCCCCCCGGCCCCCAGTCGACCAGACCCCCACCGTCGCCCAC 120
Ovary                GCCCCCACCATTCTGGGTTCCCCCCGGCCCCCAGTCGACCAGACCCCCACCGTCGCCCAC 120
                     ***  * *****************************************************

SPERM                CGACACACTCACACACACGCTCTCACGCGCTCAGCCTCTGGCCTGGGGGCGGGGGCTGGG 180
Testis               CGACACACTCACACACACGCTCTCACGCGCTCAGCCTCTGGCCTGGGGGCGGGGGCTGGG 180
Ovary                CGACACACTCACACACACGCTCTCACGCGCTCAGCCTCTGGCCTGGGGGCGGGGGCTGGG 180
                     ************************************************************

SPERM                TCTCGCCTTAGGTGGCCAAGCCGAAGGGGACCTTTGAGACCTCGGATGCGAGGAAGTCTT 240
Testis               TCTCGCCTTAGGTGGCCAAGCCGAAGGGGACCTTTGAGACCTCGGATGCGAGGAAGTCTT 240
Ovary                TCTCGCCTTAGGTGGCCAAGCCGAAGGGGACCTTTGAGACCTCGGATGCGAGGGAGTCTT 240
                     ***************************************************** ******

SPERM                GGGGTCCCCACGGGTGATTGCCAGCCCCAGGGCACTGCGCCCAGCCGGGGCCCGCCGGCC 300
Testis               GGGGTCCCCACGGGTGATTGCCAGCCCCAGGGCACTGCGCCCAGCCGGGGCCCGCCGGCC 300
Ovary                GGGGTCCCCACGGGTGATTGCCAGCCCCAGGGCACTGCGCCCAGCCGGGGCCCGCCGGCC 300
                     ************************************************************

SPERM                CAGGGCTGACGCCCCCTCCCTTAGGGAAGCTGCTTCTTCCGCACGGCCTCTCTCTGAGGT 360
Testis               CAGGGCTGACGCCCCCTCCCTTAGGGAAGCTGCTTCTTCCGCACGGCCTCTCTCTGAGGT 360
Ovary                CACGGCTGACGCCCCCTCCCTTAGGGAAGCTGCTTCTTCCGCACGGCCTCTCTCTGAGGT 360
                     ** *********************************************************

SPERM                GTGCCACGGTCTGTGCCAGTGTGTCGATCTCGGAGCGGATGCCATCCCCTGTCCCCTGTC 420
Testis               GTGCCACGGTCTGTGCCAGTGTGTCGATCTCGGAGCGGATGCCATCCCCTGTCCCCTGTC 420
Ovary                GTGCCACGGTCTGTGCCAGTGTGTCGATCTCGGAGCGGATGCCATCCCCTGTCCCCTGTC 420
                     ************************************************************

SPERM                CTTCCTTCTCCCGTTCTCCGGCTCTCTGTCAGTCGCTCTGTGTCTCCCTGTCTCTGTCTC 480
Testis               CTTCCTTCTCCCGTTCTCCGGCTCTCTGTCAGTCGCTCTGTGTCTCCCTGTCTCTGTCTC 480
Ovary                CTTCCTTCTCCCGTTCTCCGGCTCTCTGTCAGTCGCTCTGTGTCTCCCTGTCTCTGTCTC 480
                     ************************************************************

SPERM                TCCCTCCCCGTGTGTGCCTGCGCGCTCGTGTCTGTCTGTCTGT 523
Testis               TCCCTCCCCGTGTGTGCCTGCGCGCTCGTGTCTGTCTGTCTGT 523
Ovary                TCCCTCCCCGTGTGTGCCTGCGCGCTCGTGTCTGTCTGTCTGT 523
                     *******************************************
```
