# Supplementary material for: Organization and differential expression of the GACA/GATA tagged somatic and spermatozoal transcriptomes in Buffalo Bubalus bubalis
Source: BMC Genomics. 2008 Mar 20;9:132. doi: 10.1186/1471-2164-9-132 (PMC2346481; doi:10.1186/1471-2164-9-132)
Supplement: Additional file 10 — Multiple sequence alignment of GATA-tagged 800 bp novel transcript originating from different tissues and spermatozoa. Note the single nucleotide variations/INDELS spread throughout the sequence. The variations common to tissues are highlighted in blue color and that shared by sperm in red. Note the exclusive and major insertions of 14 bp in spleen, highlighted in blue background. [file 1471-2164-9-132-S10.pdf]

Additional file 10: Multiple nucleotide sequence alignment of GATA-tagged novel transcript of 800 bp originating from different tissues and spermatozoa of buffalo

```
SPERM           ---GATAGATAGATAGATAGATAGATAGATACATATGTATATATCTATGTGTGTGTATGC 57
Testis          -------GATAGATAGATAGATAGATAGATACATATGTATATATCTATGTGTGTGTATGC 53
Spleen          -------GATAGATAGATAGATAGATAGATACATATGTATATATCTATGTGTGTGTATGC 53
Ovary           -------GATAGATAGATAGATAGATAGATACATATGTATATATCTATGTGTGTGTATGC 53
Liver           -------GATAGATAGATAGATAGATAGATACATATGTATATATCTATGTGTGTGTATGC 53
Kidney          -------GATAGATAGATAGATAGATAGATACATATGTATATATCTATGTGTGTGTATGC 53
                       *****************************************************

SPERM           AAACATACACACACACAAATGGATGTAATTTTTTTTT-AATCACCACTTTGCAACCACAC 116
Testis          AAACATACACACACACAAATGGATGTAATTTTTTTTT-AATCACCACTTTGCAACCACAC 112
Ovary           AAACATACACACACACAAATGGATGTAATTTTTTTTT-AATCACCACTTTGCAACCACAC 112
Spleen          AAACATACACACACACAAATGGATGTAATTTTTTTTT-AATCACCACTTTGCAACCACAC 112
Liver           AAACATACACACACACAAATGGATGTAATTTTTTTTT-AATCACCACTTTGCAACCACAC 112
Kidney          AAACATACACACACACAAATGGATGTAATTTTTTTTTTAATCACCACTTTGCAACCACAC 113
                ************************************* **********************

SPERM           TCGTAACAATTAACTGAGGTGGGATGCACCCATGAATGTGAAACTAGCGGGCAAACATTT 176
Testis          TCGTAACAATTAACTGAGGTGGGATGCACCCATGAATGTGAAACTAGCGGGCAAACATTT 172
Ovary           TCGTAACAATTAACTGAGGCGGGATGCACCCATGAATGTGAAACTAGCGGGCAAACATTT 172
Spleen          TCGTAACAATTAACTGAGGTGGGATGCACCCATGAATGTGAAACTAGCGGGCAAACATTT 172
Liver           TCGTAACAATTAACTGAGGTGGGATGCACCCATGAATGTGAAACTAGCGGGCAAACATTT 172
Kidney          TCGTAACAATTAACTGAGGTGGGATGCACCCATGAATGTGAAACTAGCGGGCAAACATTT 173
                ******************* ****************************************

SPERM           GATGAGGACAAACTATTGACAGAATCTCAAAAGATCTTGCCAGAAATGACTGACTAATTT 236
Testis          GATGAGGACAAACTATTGACAGAATCTCAAAAGATCTTGCCAGAAATGACTGACTAATTT 232
Ovary           GATGAGGACAAACTATTGACAGAATCTCAAAAGATCTTGCCAGAAATGACTGACTAATTT 232
Spleen          GATGAGGACAAACTATTGACAGAATCTCAAAAGATCTTGCCAGAAATGACTGACTAATTT 232
Liver           GATGAGGACAAACTATTGACAGAATCTCAAAAGATCTTGCCAGAAATGACTGACTAATTT 232
Kidney          GATGAGGACAAACTATTGACAGAATCTCAAAAGATCTTGCCAGAAGTGACTGACTAATTT 233
                ********************************************* **************

SPERM           CAATGAAAGATAACATTACAGTGGAAATCTTGGCAGATACAGCCTTAACCAAATGACGAC 296
Testis          CAATAAAAAATAACATTACAGTGGAAATCTTGGCAGATACAGCCTTAACCAAATGACAAC 292
Ovary           CAATAAAAAATAACATTACAGTGGAAATCTTGGCAGATACAGCCTTAACCAAATGACAAC 292
Spleen          CAATAAAAAATAACATTACAGTGGAAATCTTGGCAGATACAGCCTTAACCAAATGACAAC 292
Liver           CAATGAAAAATAACATTACAGTGGAAATCTTGGCAGATACAGCCTTAACCAAATGACAAC 292
Kidney          CAATGAAAAATAACATTACAGTGGAAATCTTGGCAGATACAGCCTTAACCAAATGACGAC 293
                **** *** ************************************************ **

SPERM           ACTAATAATGAGAAATACCAACATCAGGTGCCTCCCAGCATGAAAAAAACACGTCATGTA 356
Testis          ACTAATAATGAGAAATACCAACATCAGGTGCCTCCCAGCATGAAAAAAACACGTCATGTA 352
Ovary           ACTAATAATGAGAAATACCAACATCAGGTGCCTCCCAGCATGAAAAAAACACGTCATGTA 352
Spleen          ACTAATAATGAGAAATACCAACATCAGGTGCCTCCCAGCATGAAAAAAACACGTCATGTA 352
Liver           ACTAATAATGAGAAATACCAACATCAGGTGCCTCCCAGCATGAAAAAAACACGTCATGTA 352
Kidney          ACTAATAATGAGAAATACCAACATCAGGTGCCTCCCAGCATGAAAAAAACACGTCATGTA 353
                ************************************************************

SPERM           TGTGATGTTCTTGCCAAAAATTGCATCGTCTGCATCTGGGCATGAAGACACATCAGCTCC 416
Testis          TGTGGTGTTCTTGCCAAAAATTGCATCGTCTGCATCTGGGCATGAAGACACATCAGCTCC 412
Ovary           TGTGGTGTTCTTGCCAAAAATTGCATCGTCTGCATCTGGGCATGAAGACACATCAGCTCC 412
Spleen          TGTGGTGTTCTTGCCAAAAATTGCATCGTCTGCATCTGGGCATGAAGACACATCAGCTCC 412
Liver           TGTGGTGTTCTTGCCAAAAATTGCATCGTCTGCATCTGGGCATGAAGACACATCAGCTCC 412
Kidney          TGTGATGTTCTTGCCAAAAATTGCATCGTCTGCATCTGGGTATGAAGACACATCAGCTCC 413
                **** *********************************** *******************

SPERM           TCTAAACGAGTCAACACAAATGTCTACTCCTTGAA--------------ACAGAAAAATA 462
Testis          TCTAAACGAGTCAACACAAATGTCTACTCCTTGAA--------------ACAGAAAAATA 458
Ovary           TCTAAACGAGTCAACACAAATGTCTACTCCTTGAA--------------ACAGAAAAGTA 458
Spleen          TCTAAACGAGTCAACACAAATGTCTACTCCTTGAAACAGAAACCTTGAAACAGAAAAATA 472
```

```
Liver           TCTAAACGAGTCAACACAAATGTCTACTCCTTGAA--------------ACAGAAAAATA 458
Kidney          TCTAAACGAGTCAACACAAATGTCTACTCCTTGAA--------------ACAGAAAAATA 459
                ***********************************              ******** **

SPERM           AACTAAACAAACTAAAGAGATACAACAACTAAGTGCATTGTGTGATCCTGTACTGGCTCC 522
Testis          AACTAAACAAACTAAAGAGATACAACAACTAAGTGCATTGTGTGATCCTGTACTGGCTCC 518
Ovary           AACTAAACAAACTAAAGAGATACAACAACTAAGTGCATTGTGCGATCCTGTACTGGCTCC 518
Spleen          AACTAAACAAACTAAAGAGATACAACAACTAAGTGCATTATGTGATCCTGTACTGGCTCC 532
Liver           AACTAAACAAACTAAAGAGATACAACAACTAAGTGCATTGTGTGATCCTGTACTGGCTCC 518
Kidney          AACTAAACAAACTAAAGAGATACAACAACTAAGTGCATTGTGTGATCCTGTACTGGCTCC 519
                *************************************** ** *****************

SPERM           CTGAGTGGAAAAAAGTTATAAAGAACACTGTCGGGAAAGTTGGAAATTTTTGAATAATAA 582
Testis          CTGAGTGGAAAAAAGTTATAAAGAACACTGTCGGGAAAGTTGGAAATTTTTGAATAATAA 578
Ovary           CTGAGTGGAAAAAAGTTATAAAGAACACTGTCGGGAAAGTTGGAAATTTTTGAATAATAA 578
Spleen          CTGAGTGGAAAAAAGTTATAAAGAACACTGTCGGGAAAGTTGGAAATTTTTGAATAATAA 592
Liver           CTGAGTGGAAAAAAGTTATAAAGAACACTGTCGGGAAAGTTGGAAATTTTTGAATAATAA 578
Kidney          CTGAGTGGAAAAAAGTTATAAAGAACACTGTCGGGAAAGTTGGAAATCTTTGAATAATAA 579
                *********************************************** ************

SPERM           TAATGTATCAAATAATAATATTGTATCAAGATTAAATAGCCTAATATTTATAATCATATT 642
Testis          TAATGTATCAAATAATAATATTGTATCAAGATTAAATAGCCTAATATTTATAATCATATT 638
Ovary           TAATGTATCAAATAATAATATTGTATCAAGATTAAATAGCCTAATATTTATAATCATATT 638
Spleen          TAATGTATCAAATAATAATATTGTATCAAGATTAAATAGCCTAATATTTATAATCATATT 652
Liver           TAATGTATCAAATAATAATATTGTATCAAGATTAAATAGCCTAATATTTATAATCATATT 638
Kidney          TAATGTATCAAATAATAATATTGTATCAAGATTAAATAGCCTAATATTTATAATCATATT 639
                ************************************************************

SPERM           AGAAATATTGCATTCTTTGGAAATATGCACTAAAGTGTTGGGATAAAGAGCATGATATCT 702
Testis          AGAAATATTGCATTCTTTGGAAATATGCACTAAAGTGTTGGGATAAAGAGCATGATATCT 698
Ovary           AGAAATATTGCATTCTTTGGAAATATGCACTAAAGTGTTGGGATAAAGAGCATGATATCT 698
Spleen          AGAAATATTGCATTCTTTGGAAATATGCACTAAAGTGTTGGGATAAAGAGCATGATATCT 712
Liver           AGAAATATTGCATTCTTTGGAAATATGCACTAAAGTGTTGGGATAAAGAGCATGATATCT 698
Kidney          AGAAATATTGCATTCTTTGGAAATATGCACTAAAGTGTTGGGATAAAGAGCATGATATCT 699
                ******* ****************************************************

SPERM           GTAGCTTACTCCAAAAGCGTAGGAAAAATGTTAATTCGCAGTATGTCCCTTATTTGTCTA 762
Testis          GTAGCTTACTCCAAAAGCGTAGGAAAAATGTTAATTCGCAGTATGTCCCTTATTTGTCTA 758
Ovary           GTAGCTTACTCCAAAAGCGTAGGAAAAATGTTAATTCGCAGTATGTCCCTTATTTGTCTA 758
Spleen          GTAGCTTACTCCAAAAGCGTAGGAAAAATGTTAATTCGCAGTATGTCCCTTATTTGTCTA 772
Liver           GTAGCTTACTCCAAAAGCGTAGGAAAAATGTTAATTCGCAGTATGTCCCTTATTTGTCTA 758
Kidney          GTAGCTTACTCCAAAAGCGTAGGAAAAATGTTAATTCGCAGTATGTCCCTTATTTGTCTA 759
                ************************************************************

SPERM           TCATCTATCTATCTATGTATCTACCTATCTATCTATCTATCTATCTATC 811
Testis          TCATCTATCTATCTATGTATCTACCTATCTATCTATCTATCTATCTATC 807
Ovary           TCATCCATCTATCTATGTATCTACCTATCTATCTATCTATCTATCTATC 807
Liver           TCATCTATCTATCTATGTATCTACCTATCTATCTATCTATCTATCTATC 807
Kidney          TCATCTATCTATCTATGTATCTACCTATCTATCTATCTATCTATCTATC 808
Spleen          TCATCTATCTATCTATGTATCTACCTATCTATCTATCTATCTATCTATC 821
                ***** ******************************************
```
