# Supplementary material for: Organization and differential expression of the GACA/GATA tagged somatic and spermatozoal transcriptomes in Buffalo Bubalus bubalis
Source: BMC Genomics. 2008 Mar 20;9:132. doi: 10.1186/1471-2164-9-132 (PMC2346481; doi:10.1186/1471-2164-9-132)
Supplement: Additional file 11 — Multiple sequence alignment of GATA-tagged 425 bp novel transcript originating from different tissues and spermatozoa. The sequence from spermatozoa is highlighted in yellow background. The variations common to few tissues are highlighted in same color (blue or red). [file 1471-2164-9-132-S11.pdf]

Additional file 11: Multiple sequence alignment of GATA-tagged novel 425 bp transcript originating from different tissues and spermatozoa of buffalo

```
Sperm           GATAGATAGATAGATAGATAGATACTGATTGAATGGATGAAAGATACTTTGAAATATGTT 60
Testis          GATAGATAGATAGATAGATAGATACTGATTGAATGGATGAAAGATACTTTGAAATATGTT 60
Ovary           GATAGATAGATAGATAGATAGATACTGATTGAATGGATGAAAGATACTTTGAAATATGTT 60
Spleen          GATAGATAGATAGATAGATAGATACTGATTGAATGGATGAAAGATACTTTGAAATATGTT 60
Liver           GATAGATAGATAGATAGATAGATACTGATTGAATGGATGAAAGATACTTTGAAATATGTT 60
Kidney          GATAGATAGATAGATAGATAGATACTGATTGAATGGATGAAAGATACTTTGAAATATGTT 60
                ************************************************************

Sperm           ATTTTGAAGCTAACGTTACGAGATAAAACAGATTGGAAATTATGAATAGTGGTTTTTGTG 120
Testis          ATTTTGAAGCTAACGTTACGAGATAAAACAGATTGGAAATTATGAATAGTGGTTTTTGTG 120
Ovary           ATTTTGAAGCTAACGTTACGAGATAAAACAGATTGGAAATTATGAATAGTGGTTTTTGTG 120
Spleen          ATTTTGAAGCTAACGTTACGAGATAAAACAGATTGGAAATTATGAATAGTGGTTTTTGTG 120
Liver           ATTTTGAAGCTAACGTTACGAGATAAAACAGATTGGAAATTATGAATAGTGGTTTTTGTG 120
Kidney          ATTTTGAAGCTAACGTTACGAGATAAAACAGACTGGAAATTATGAATAGTGGTTTTTGTG 120
                ******************************** ***************************

Sperm           TCCTGCAGTTCTCTGAACTGGACTATGTTGTGAGAAAATAAATAAAATGTTTAAGATTAC 180
Testis          TCCTGCAGTTCTCTGAACTGGACTATGTTGTGAGAAAATAAATAAAATGTTTAAGATTAC 180
Ovary           TCCTGCAGTTCTCTGAACTGGACTATGTTGTGAGAAAATAAATAAAATGTTTAAGATTAC 180
Spleen          TCCTGCAGTTCTCTGAACTGGACTATGTTGTGAGAAAATAAATAAAATGTTTAAGATTAC 180
Liver           TCCTGCAGTTCTCTGAACTGGACTATGTTGTGAGAAAATAAATAAAATGTTTAAGATTAC 180
Kidney          TCCTGCAGTTCTCTGAACTGGACTATGTTGTGAGAAAATAAATAAAATGTTTAAGATTAC 180
                ************************************************************

Seprm           AGATTTAAAATTGGACAAACTCAGGTTTGATTCCTACTTCTGCTTGAACTTGAACAAATT 240
Testis          AGATTTAAAATTGGACAAACTCAGGTTTGATTCCTACTTCTGCTTGAACTTGAACAAATT 240
Ovary           AGATATAAAATTGGACAAACTCAGGTTTGATTCCTACTTCTGCTTGAACTTGAACAAATT 240
Spleen          AGATTTAAAATTGGACAAACTCAGGTTTGATTCCTACTTCTGCTTGAACTTGAACAAATT 240
Liver           AGATTTAAAATTGGACAAACTCAGGTTTGACTCCTACTTCTGCTTGAACTTGAACAAATT 240
Kidney          AGATTTAAAATTGGACAAACTCAGGTTTGATTCCTACTTCTGCTTGAACTTGAACAAATT 240
                **** ************************* *****************************

Sperm           ACTTAAATTTTCTAGGCACTTTCCTTTTGTTATAGTAGTTTGTAGTATTCATAATATAGC 300
Testis          ACTTAAATTTTCTAGGCACTTTCCTTTTGTTATAGTAGTTTGTAGTATTCATAATATAGC 300
Ovary           ACTTAAATTTTCTAGGCACTTTCCTTTTGTTATAGTAGTTTGTAGTATTCATAATATAGC 300
Spleen          ACTTAAATTTTCTAGGCACTTTCCTTTTGTTATAGTAGTTTGTAATATTCATAATACAGC 300
Liver           ACTTAAATTTTCTAGGCACTTTCCTTTTGTTATAGTAGTTTGTAATATTCATAATACAGC 300
Kidney          ACTTAAATTTTCTAGGCACTTTCCTTTTGTTATAGTAGTTTGTAATATTCATAATACAGC 300
                ******************************************** *********** ***

Sperm           ATAGGTGAAAGGATTAAATATGGAGGCATTCAAAACTATCTGGTTTACAGCATACCCAGA 360
Testis          ATAGGTGTAAGGATTAAATATGGAGGCATTCAAAACTATCTGGTTTACACCGTACCCAGA 360
Ovary           ATAGGTGTAAGGATTAAATATGGAGGCATTCAAAACTATCTGGTTTACACCGTACCCAGA 360
Spleen          ATAGGTGAAAGGATTAAATATGGAGGCATTCAAAACTATCTGGTTTACAGCATACCCAGA 360
Liver           ATAGGTGAAAGGATTAAATATGGAGGCATTCAAAACTATCTGGTTTACAGCATACCCAGA 360
Kidney          ATAGGTGAAAGGATTAAATATGGAGGCATTCAAAACTATCTGGTTTACAGCATACCCAGA 360
                ******* ***************************************** * ********

Sperm           ACAAAGGTAATGTTCTGTAATATCAGCTATTCATATATGTGTATCTATCTATCTATCTAT 420
Testis          ACAAAGGTAATGTTCTGTAATATCAGCTATTCATATATGTGTATCTATCTATCTATCTAT 420
Ovary           ACAAAGGTAATGTTCTGTAATATCAGCTATTCATATATGTGTATCTATCTATCTATCTAT 420
Spleen          ACAAAGGTAATGTTCTGTAATATCAGCTATTCATATATGTGTATCTATCTATCTATCTAT 420
Liver           ACAAAGGTAATGTTCTGTAATATCAGCTATTCATATATGTGTATCTATCTATCTATCTAT 420
Kidney          ACAAAGGTAATGTTCTGTAATATCAGCTATTCATATATGTGTATCTATCTATCTATCTAT 420
                ************************************************************

Sperm           CTATC 425
Testis          CTATC 425
Ovary           CTATC 425
Spleen          CTATC 425
Liver           CTATC 425
Kidney          CTATC 425
                *****
```
