# Supplementary figures and images for: Organization and differential expression of the GACA/GATA tagged somatic and spermatozoal transcriptomes in Buffalo Bubalus bubalis
Source: BMC Genomics. 2008 Mar 20;9:132. doi: 10.1186/1471-2164-9-132 (PMC2346481; doi:10.1186/1471-2164-9-132)

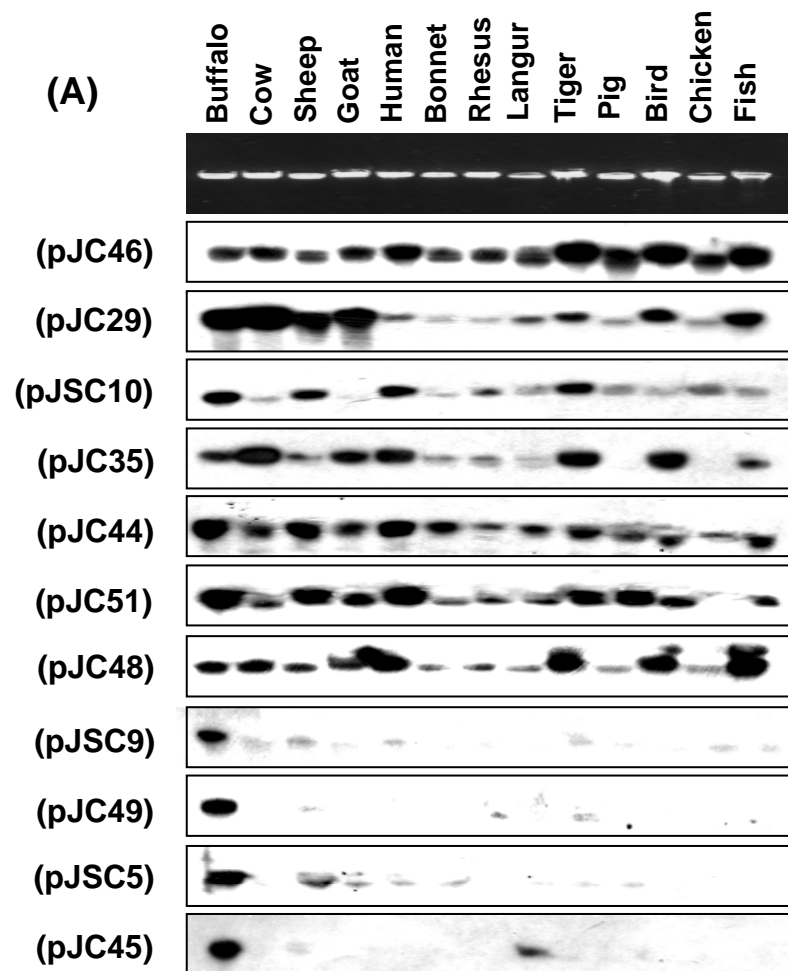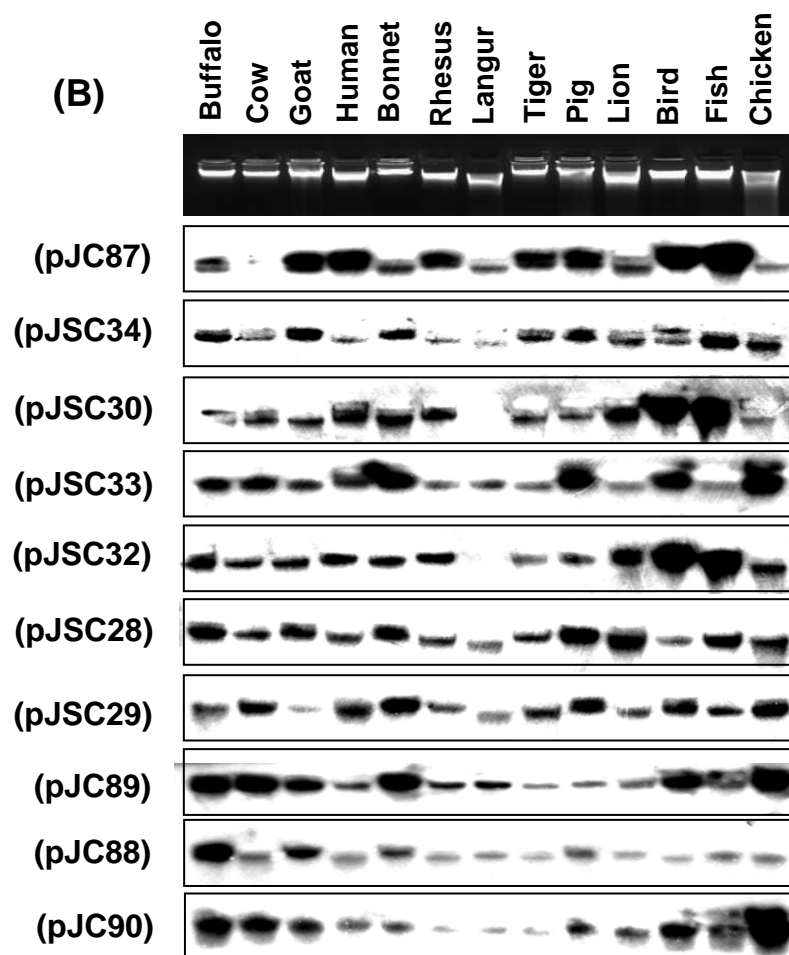

**Additional file 12: Evolutionary conservation of GACA/GATA tagged transcripts across species**

Supplement: Additional file 12 — Cross-hybridization of genomic DNA from different species with the recombinant clones containing GACA (A) and GATA (B) uncovered genes/gene fragments. The names of the species are given on the top, and the autoradiograms for the respective gene/gene fragments on the left. Note the conservation of all the GATA and ~75% GACA uncovered genes across the species whereas remaining GACA-tagged transcripts were specific to buffalo/Bovids. [file 1471-2164-9-132-S12.pdf]
